# Protein allosteric site identification using machine learning and per amino acid residue reported internal protein nanoenvironment descriptors

**DOI:** 10.1016/j.csbj.2024.10.036

**Published:** 2024-10-23

**Authors:** Folorunsho Bright Omage, José Augusto Salim, Ivan Mazoni, Inácio Henrique Yano, Luiz Borro, Jorge Enrique Hernández Gonzalez, Fabio Rogerio de Moraes, Poliana Fernanda Giachetto, Ljubica Tasic, Raghuvir Krishnaswamy Arni, Goran Neshich

**Affiliations:** a Computational Biology Research Group, Embrapa Digital Agriculture, Campinas, São Paulo, Brazil; b Biological Chemistry Laboratory, Department of Organic Chemistry, Institute of Chemistry, University of Campinas (UNICAMP), Campinas, São Paulo, Brazil; c Department of Plant Biology, Institute of Biology, University of Campinas (UNICAMP), Campinas, São Paulo, Brazil; d São Paulo State University (UNESP), Institute of Biosciences, Humanities and Exact Sciences, São José do Rio Preto

**Keywords:** Allosteric sites, Machine learning, Protein structure analysis, Computational drug design, Internal protein nanoenvironment, STING descriptors, Distance center center

## Abstract

Allosteric regulation plays a crucial role in modulating protein functions and represents a promising strategy in drug development, offering enhanced specificity and reduced toxicity compared to traditional active site inhibition. Existing computational methods for predicting allosteric sites on proteins often rely on static protein surface pocket features, normal mode analysis or extensive molecular dynamics simulations encompassing both the protein function modulator and the protein itself. In this study, we introduce an innovative methodology that employs a per amino acid residue classifier to distinguish allosteric site-forming residues (AFRs) from non-allosteric, or free residues (FRs). Our model, STINGAllo, exhibits robust performance, achieving Distance Center Center (DCC) success rate when all AFRs were predicted within pockets identified by FPocket, overall DCC, F1 score and a Matthews correlation coefficient (MCC) of 78 %, 60 %, 64 % and 64 % respectively. Furthermore, we identified key descriptors that characterize the internal protein nanoenvironment of AFRs, setting them apart from FRs. These descriptors include the sponge effect, distance to the protein centre of geometry (cg), hydrophobic interactions, electrostatic potentials, eccentricity, and graph bottleneck features.

## Graphical Abstract

**Figure:**
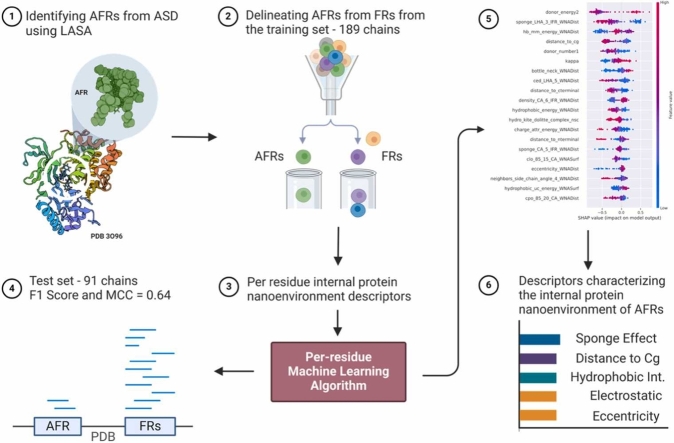

## 1 Introduction

Allosteric sites are crucial for the complex regulation of protein functions; they are not involved in the primary enzymatic reactions but play significant roles in modulating a protein’s activity through the binding of allosteric modulators. This modulation can alter the behaviour of the protein’s active site and its overall activity. Drug design has predominantly focused on targeting these orthosteric (active) sites of proteins to inhibit their enzyme activity. The considerable conservation of catalytic and active sites within protein families can result in low selectivity and an increased likelihood of off-target effects [1]. Allosteric modulation does come with several benefits. A representative example is the orthosteric site targets of protease from SARS-CoV-2, where the active site carries the activated cysteine (Cys-S-) residue and targeting it pose the risk of cross-reactivity with other enzymes featuring in their active sites an activated cysteine residue. This will be further complicated by the possible interactions with abundant proteins, including albumin, and small molecules containing free thiol groups, such as cysteine and glutathione, making the design of selective inhibitors for SARS-CoV-2 proteases an even more formidable task [2], [3], [4], [5], [6], [7]. The allosteric sites are generally less conserved among protein families, which makes the design of highly selective modulators with few unwanted side effects often easier to obtain. Besides this, such allosteric modulators also possess a unique ability to subtly modulate protein activities rather than simply turning on or being inhibited; this allows much finer control of biological processes. It is this plausible regulation that has value in therapeutic contexts, where the precise modulation of protein function is quite important. In recent years, much pharmaceutical research has focused on the identification and development of allosteric modulators capable of achieving specific tuning of protein functions at very high selectivity. Of the 188 pharmaceuticals approved by the Food and Drug Administration between 2018 and 2022, 22 were classified as being allosteric drugs [8].

Until now, traditional means of identifying allosteric sites through X-ray crystallography, nuclear magnetic resonance spectroscopy, and cryo-electron microscopy have given way to detailed structural insight and thus were instrumental in defining protein architectures while identifying potential binding sites. Complementing these techniques are molecular dynamics simulations and normal-mode analysis that enable similarly detailed descriptions of protein motion and conformational flexibility. Although MD simulations have provided an atomistic insight into protein dynamics, these usually require substantial resource and computational time. Simulation of microseconds scale dynamics may run for weeks, as a function of system size and resource availability. On the other hand, machine learning methods have benefit of generating high-throughput predictions after their usually extensive training phase, really cutting down the time of the analysis when compared to other methods. The training phase, for hyperparameter optimization, is also likely to be computationally expensive. Unlike Structure-Based Statistical Mechanical Models (SBSMMA)[9], which rely on detailed structural and energetic information as implemented in methods such as AlloSigMA [10] and AlloMAPS [11], machine learning algorithms can study much larger and more diverse datasets to capture complicated patterns inherent in protein dynamics, enabling machine learning to be more effective, especially in the scalable and flexible prediction of allostery sites across a wide array of protein families. Besides, machine learning models are able to reveal new knowledge hardly achievable or just impossible by traditional physics-based methods. A few pioneering works have defined the state-of-the-art for ML-based allosteric site predictions. For instance, the first model, Allosite introduced in 2013 [12], uses SVM with the physicochemical features obtained from the program FPocket [13] to predict allosteric pockets. Subsequent models, such as AlloPred [14] and AllositePro [15], further augmented those with NMA-derived metrics and logistic regression. Residue-based models, such as NACEN [16] and AR-Pred[17] have attained higher accuracy by giving more attention to residues in allosteric states with a random forest algorithm combined with network-oriented features. However, there are considerable challenges that face machine learning models, of which the most important regard the quality and heterogeneity of the training datasets. Most of these datasets, as representatives in ASD [18], [19], are narrow and biased toward well-studied proteins. More serious are ambiguities in amino acid residue numbering within regulatory sites, incomplete lists of AFRs, and intrinsic class imbalances complicating model training, which lead to a reduction in predictive accuracy [8], [20], [21]. These limitations decrease the capability of models to predict allosteric sites accurately. Here, we adopt a residue-based method whereby we generate, for each of the 20 types of amino acid residues present in the training set, a model that captures the unique internal nanoenvironment within the protein. We have identified, using the LASA - loss of accessible surface area, the residues lining the allosteric site of the proteins present in the ASBench, a reference or benchmarked dataset for ASD[22], which interact with the modulators. The purpose is not to miss any AFRs, not to commit Type II errors; thus, no potential AFR must be left behind. In this respect, we here use the concept of protein nanoenvironment - despite the fact that the term was coined by us two decades ago-describes functional regions of proteins with special physicochemical and structural features. These regions are defined by distinct STING descriptors [23], [24], [25] and were previously shown to dictate key functional properties of proteins, including for enzymatic activity at active sites and at protein interfaces, as well as for secondary structure elements, by descriptor characterization of amino acids belonging to corresponding internal protein nanoenvironment [23], [24], [25], [26], [27], [28], [29], [30], [31]. To explore the internal protein nanoenvironment of AFRs in proteins, we utilized various machine learning techniques to differentiate between AFRs and FRs. Our approach culminates in classifying AFRs as distinct entities from FRs, based on an extensive analysis of several physical, chemical, and structural descriptors of internal protein nanoenvironment available in the STING database[23], [24], [31], [32]. The STING database, developed based on extensive studies carried out in our laboratory, represents the largest repository of physical, chemical, and structural descriptors of proteins extracted from the Protein Data Bank. In this work we analyse general properties of AFRs with respect to their internal nanoenvironment, as defined by the STING descriptors calculated from static 3-D protein structures deposited in the PDB and STING database. Our approach does not explicitly take into account the protein dynamics. Yet, as we will show, it captures the key nanoenvironment features differentiating AFRs from FRs.

## 2 Materials and methods

### 2.1 Dataset preparation and characterization

A total of 235 X-ray crystallographic structures of allosteric proteins were retrieved from the ASBench database[22]. This dataset included experimentally verified active and allosteric site complexes. Initial data processing involved filtering out entries lacking complete active/allosteric site data or those with partial structural information. The refined dataset comprised 230 protein structures and 376 chains, with an overall average of approximately 203 residues per chain and a standard deviation of 61 residues.

AFRs are defined as specific residues within a protein that participate in allosteric regulation by interacting with allosteric modulators. The Loss of Accessible Surface Area (LASA) method is employed to identify AFRs by measuring the change in accessible surface area of a residue upon complex formation. LASA quantifies the difference in a residue's accessible surface area (ASA) between its unbound state (apo) and its state within a protein-ligand complex. The LASA calculation is defined by the equation:

LASA=ASAapo−ASAcomplex

where ASAapo represents the ASA of a residue in the protein’s unbound state, and ASAcomplex indicates the ASA within a protein-ligand complex, and both pivotal for identifying key residues at the allosteric interface.

The LASA result and AFR annotations are documented in Supplementary S1[33]. To calculate LASA, we set a threshold of 5 Å, commonly recognized as an optimum range for meaningful non-bonding interactions critical in maintaining the modulator binding to the allosteric site[34], [35]. The LASA calculation utilized both holo-structures (bound state) and apo-structures (unbound state) directly retrieved from the ASBench dataset. By comparing the accessible surface area of each residue in both the holo and apo states provided by ASBench, we were able to identify significant changes in surface accessibility that indicate potential allosteric site-forming residues. To maintain the structural and functional coherence of protein residues, our methodology adopts a chain-centric splitting strategy. This approach prevents data leakage by segregating the dataset based on unique protein chains. A composite identifier, formed by concatenating the PDB code and the chain name, serves as the basis for this segregation. This unique composite key guarantees that only one instance of a chain can occur in any given subset, training, validation, or testing, thereby preventing overlap across these sets. Moreover, the dataset we utilized, ASBench, is a benchmark derived from the Allosteric Database (ASD) and has been meticulously curated to ensure that the sequence similarity between proteins is less than 30 %[22]. This stringent threshold minimizes the risk of having structurally similar proteins in both the training and test sets, which could otherwise bias the feature extraction and model performance. Given that the majority of the dataset consists of single-chain proteins (79.8 %), with only 20.2 % comprising multi-chain proteins, the likelihood of similar chains appearing in both the training and test sets is further reduced. This approach guarantees the independence and consistency of the data distribution, crucial for preventing overestimation of the model's performance. The chain-centric split method yielded three distinct subsets:

• **Training Set:** This set comprises 189 chains, accounting for 50.3 % of the total dataset, with an overall average of approximately 192 residues per chain and a standard deviation of 70 residues.
• **Validation Set:** Consisting of 96 chains, the validation set makes up 25.5 % of the dataset, with an overall average of approximately 210 residues per chain and a standard deviation of 60 residues.
• **Testing Set:** The testing set includes 91 chains, which constitutes 24.2 % of the dataset, with an overall average of approximately 211 residues per chain and a standard deviation of 58 residues.

The validation set was used to tune the models. After hyperparameter tuning, the final evaluation of the model’s performance was conducted using the test set.

#### 2.1.1 Characterization of AFRs with STING descriptors and statistical analysis

The internal nanoenvironment of AFRs was quantitatively characterized using a comprehensive suite of STING descriptors[29], [31], [36]. These descriptors provide detailed insights into the biochemical and structural properties of protein residues within their three-dimensional (3D) context. Key features, such as solvent accessibility, hydrophobic interactions, and spatial relationships, are captured to differentiate the internal nanoenvironment of AFRs from that of FRs. The selected descriptors are described in detail in Supplementary S2[37]. At the core of our investigation is the hypothesis that the ensemble of internal protein nanoenvironment descriptors for AFRs is statistically distinct from those of FRs, reflecting differences in their biochemical and structural roles. To test this, we performed statistical analyses to evaluate the significance of differences in descriptor distributions between AFRs and FRs. The null hypothesis (H0) posits that the descriptor values for AFRs and FRs are drawn from the same distribution, while the alternative hypothesis (H1) suggests they differ. We applied the Kolmogorov-Smirnov (KS) test, a non-parametric method that compares the empirical distribution functions of two samples, to assess the maximum deviation between them, providing a robust measure of distributional divergence. Additionally, Q-Q plots were used to examine the normality of the descriptor distributions, offering a visual assessment of their conformity to normal distribution, a prerequisite for parametric tests like Student’s t-test. Details of this analysis are provided in Supplementary S3[38].

#### 2.1.2 Descriptor selection and exploratory data analysis (EDA)

Given the high dimensionality and collinearity among number of STING descriptors, we applied a correlation-based feature selection method[39] to refine our descriptor set. This method employs the Pearson correlation coefficient, denoted as *ρ*, to identify and exclude highly correlated features, thereby minimizing redundancy. From an initial database of over 1200 descriptors, we retrieved 733 that were relevant to our study, excluding those unrelated to our focus such as descriptors for DNA or other internal protein nanoenvironment not of interest. To balance the retention of informative descriptors with the reduction of collinearity, we applied a threshold of ρ ≤ 0.7, which reduced the set to 79 descriptors. Furthermore, based on the results of the Kolmogorov-Smirnov test, which identified significant distributional differences between AFRs and FRs, we retained 54 critical descriptors for machine learning application. The *ρ* parameter serves as a critical threshold in this process, ensuring the exclusion of descriptors with excessive inter-correlations that could obscure the true predictive signals in our model. Exploratory Data Analysis (EDA) was conducted to scrutinize the data and unearth underlying patterns, leveraging *pandas*, a widely used Python package for data manipulation and analysis[40].

### 2.2 Machine learning model development and evaluation

**Fig. 1:**
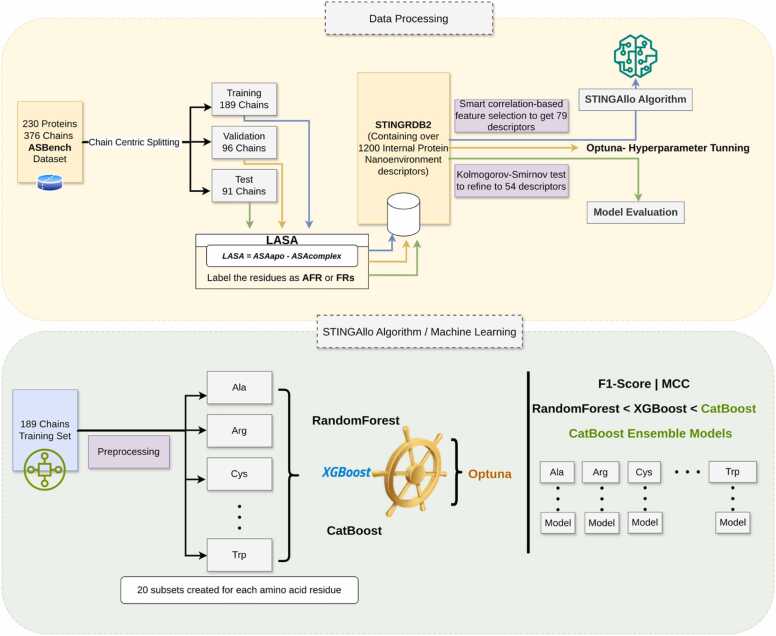
Workflow of STINGAllo, a per-amino-acid residue classifier. The dataset consists of 230 proteins (376 chains) from the ASBench database, split into training, validation, and test sets (189, 96, and 91 chains, respectively) using a chain-centric strategy. Residues are labelled as AFR or FR using the LASA method, where AFRs are defined as residues within 5 Å of the modulator molecule. Over 1200 internal protein nanoenvironment descriptors are extracted from the STING_RDB_2 database. A correlation-based feature selection process refines the descriptors to 79 orthogonal ones, further reduced to 54 critical descriptors via the Kolmogorov-Smirnov test. These descriptors are then used for model training on subsets based on individual amino acid types. Machine learning models (Random Forest, XGBoost, and CatBoost) are optimized through hyperparameter tuning using Optuna. CatBoost, which demonstrated the highest performance based on F1-Score and MCC, was selected as the core model for STINGAllo's allosteric site prediction.

Fig. 1 outlines the complete workflow, from dataset preparation to model evaluation. The STINGAllo model was trained on 20 subsets, each corresponding to a specific amino acid residue type (e.g., Ala, Arg, Cys). Several classifiers, including RandomForest, XGBoost, and CatBoost, were evaluated, with hyperparameter tuning performed using Optuna[41] CatBoost consistently achieved the highest performance, as measured by F1-Score and MCC, and was therefore selected as the final model for STINGAllo.

#### 2.2.1 CatBoost algorithm for per-residue modelling

The CatBoost algorithm minimizes a loss function *L*, typically a log-likelihood function in classification tasks, represented as:

$$L=-\sum_{i=1}^{N}y_{i}\log\left(\hat{y_{i}}\right)+\left(1-y_{i}\right)\log\left(1-\hat{y_{i}}\right)$$

where N denotes the sample size, $y_{i}$ the actual label, and $\hat{y_{i}}$ the predicted probability for the positive class of the $i^{\mathrm{th}}$ sample.

To accommodate class imbalance (1:21), the model was initialized with a *scale_pos_weight* parameter, adjusting the model’s focus to ensure a balanced treatment of both classes for each of 20 amino acid residue types. Model training entailed fitting the CatBoost Classifier to the training data for each residue type.

#### 2.2.2 Hyperparameter tuning

Hyperparameter optimization was performed using Optuna [41], utilizing 80 CPUs for 48 h to explore a wide range of parameter configurations for each model. The final search space for the key hyperparameters was as follows:

• CatBoost: Depth (3−10), learning rate (0.001–0.5), iterations (100–1000), l2_leaf_reg (1−10), border count (1−255)
• XGBoost: Max_depth (3−10), learning rate (0.001–0.5), n_estimators (100–1000), min_child_weight (1−10), colsample_bytree (0.1–1), subsample (0.1–1)
• Random Forest: n_estimators (100–1000), max_depth (3−20), min_samples_split (2−10), min_samples_leaf (1−10)

Despite the extensive tuning process, the default hyperparameters for CatBoost consistently yielded superior performance compared to the optimized versions of CatBoost, XGBoost, and Random Forest, achieving the highest F1 Scores and MCC. The final STINGAllo model utilized the default CatBoost settings, which not only ensured high predictive accuracy but also enabled rapid inference for predicting AFRs.

#### 2.2.3 STINGAllo evaluation

To evaluate the performance of our model in predicting AFRs, we calculated the Distance Center Center (DCC) [42] for four prediction sets: STINGAllo, PASSer Ensemble, PASSer Automl, and PASSer Rank. The DCC metric measures the Euclidean distance between the centroids of the predicted AFRs and the actual binding site residues. Lower DCC values represent a closer spatial match, with values below 4 Å considered indicative of a successful prediction. The DCC is given by the formula:

$$\mathrm{DCC}=\sqrt{{\left(x_{\mathrm{pred}}-x_{\mathrm{true}}\right)}^{2}+{\left(y_{\mathrm{pred}}-y_{\mathrm{true}}\right)}^{2}+{\left(z_{\mathrm{pred}}-z_{\mathrm{true}}\right)}^{2}}$$

Where ($x_{\mathrm{pred}}$, $y_{\mathrm{pred}}$, $z_{\mathrm{pred}}$) and ($x_{\mathrm{true}}$, $y_{\mathrm{true}}$, $z_{\mathrm{true}}$) are the coordinates of the predicted and actual AFRs site centroids, respectively.

The final step involved calculating the DCC for each protein in the four prediction sets. A DCC value below 4 Å was considered a successful prediction, signifying a strong alignment between the predicted and actual binding sites. The success rate for each set was then determined by the proportion of predictions with DCC ≤ 4 Å.

To further evaluate STINGAllo's performance in identifying residues, we employed additional standard metrics, each offering unique insights into different aspects of model accuracy and reliability. These metrics, derived from the fundamental elements of the confusion matrix, true positives (TP), true negatives (TN), false positives (FP), and false negatives (FN), include accuracy, F1 score, MCC, recall, precision, and ROC-AUC. Detailed information on these metrics is provided in Supplementary S4 [43].

## 3 Results

### 3.1 AFR locations relative to FPocket and nanoshaper-identified pockets

Out of the 11 models available in the literature designed to predict allosteric sites on proteins [Allosite[12], AlloPred[14], AllositePro[15], ALLO[44], PASSer[20], [45], PASSer2.0[46], PASSerRank[47], AlloReverse[48], TopoAlloSite[49], AR-Pred[17], and NACEN[16]] only two (AR-Pred and NACEN) are residue-based. The remaining models are pocket-based, utilizing data provided by the FPocket package [13]. Therefore, it is crucial to first determine whether all ASD-annotated AFRs are located within pockets identified by FPocket.

The protein chains in Table 1 include both single-chain proteins and individual chains within multi-chain complexes. The analysis reveals that 15.51 % of proteins with AFRs have residues located outside pockets identified by FPocket, in both isolated and complex forms. Notably, in 2.80 % of these cases, residues predicted to be outside the pocket in the isolated form are found within a pocket when considering the complex form. As shown in Tables 1, 18.31 % of AFRs are predicted to be located outside pockets when using FPocket and 55.90 % when using NanoShaper on isolated proteins as the case with majority of the models. This suggests that pocket models based solely on pocket data may exclude certain experimentally validated allosteric sites, particularly those that do not conform to the boundaries of pocket regions identified by FPocket or NanoShaper.

**Table 1:** AFR Locations Relative to FPocket-Identified Pockets for ASD-Annotated Sites.

Algorithm	AFR in Pocket	AFR Outside Pocket	Percentage Outside Pocket
FPocket[13]	6087	1364	18.31 %
NanoShaper[50]	3286	4165	55.90 %

### 3.2 Relationship Between Amino Acid Residue Frequency and Predictive Accuracy

**Fig. 2:**
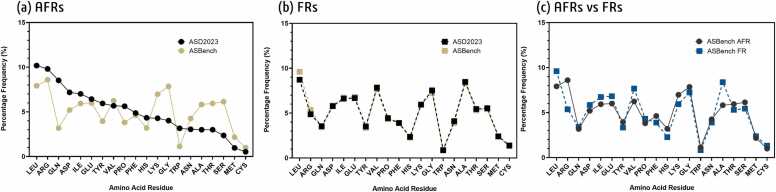
Percentage frequency distribution of amino acid residues in AFRs and FRs from the ASD2023 and ASBench datasets (a) shows the distribution of amino acids in allosteric sites, while (b) shows the distribution of FRs and (c) provides a comparison of AFRs and FRs in the ASBench dataset.

We observed notable variability in the frequency distribution of amino acid residues in allosteric sites, which likely reflects a combination of the inherent physicochemical properties of the residues, their interaction energies, and the structural environment of the protein. These factors, rather than biological preferences alone, are likely responsible for shaping the distribution patterns observed in the ASD2023 and ASBench datasets (Fig. 2). For example, residues such as Arginine (ARG) and Glycine (GLY) were more frequently represented, while Cysteine (CYS) appeared less often in allosteric sites (Fig. 2)..

**Table 2:** Performance of Residue-Based Classification on Test Set (CatBoost Model).

Residue	F1 Score	MCC	Recall	Precision
TRP	0.8095	0.8139	0.6800	1.0000
MET	0.7385	0.7329	0.6667	0.8276
ASP	0.7176	0.7204	0.6104	0.8704
HIS	0.7091	0.6941	0.6393	0.7959
THR	0.7065	0.7093	0.5856	0.8904
ARG	0.6901	0.6689	0.6364	0.7538
GLY	0.6717	0.6615	0.5894	0.7807
CYS	0.6667	0.6713	0.5500	0.8462
PHE	0.6667	0.6645	0.5543	0.8361
ASN	0.6667	0.6626	0.5694	0.8039
LYS	0.6512	0.6418	0.5773	0.7467
SER	0.6448	0.6598	0.5000	0.9077
TYR	0.6197	0.6098	0.5176	0.7719
PRO	0.6139	0.6034	0.5636	0.6739
VAL	0.6115	0.6150	0.5053	0.7742
ALA	0.5988	0.6223	0.4545	0.8772
GLU	0.5850	0.5902	0.4725	0.7679
ILE	0.5755	0.5742	0.4819	0.7143
LEU	0.5387	0.5383	0.4345	0.7087
GLN	0.5000	0.5491	0.3455	0.9048

**Fig. 3:**
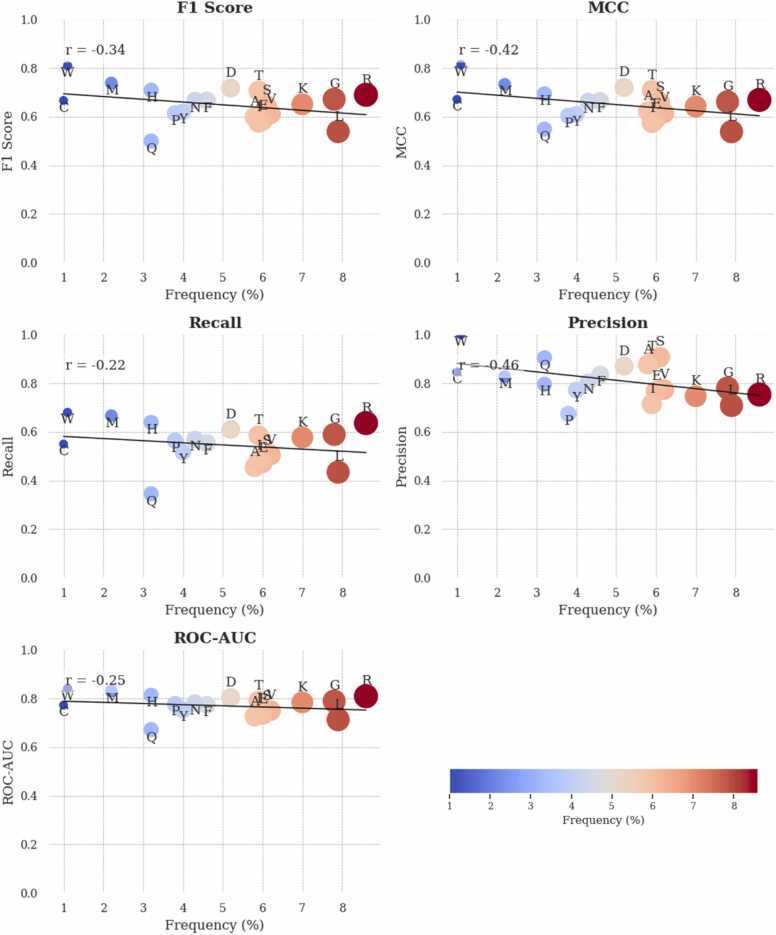
Scatter plots showing the relationship between the frequency of individual amino acid residues in ASBench and various performance metrics across per residue models for AFR prediction. The metrics include F1 Score, MCC, Recall, Precision, and ROC-AUC. Each plot visualizes residue frequency (x-axis) against a specific performance metric (y-axis). Data points represent individual residues, with point size scaled to frequency and colour coded on a gradient from blue (lower frequency) to red (higher frequency). Linear trend lines (black) are fitted to each dataset to illustrate the overall relationship between frequency and model performance, while Pearson correlation coefficients (r) are displayed to quantify the strength of this association. The separate colour bar below the figure indicates the range of frequencies in percentage.

To assess whether this variability influenced predictive model performance, we compared residue frequencies with model metrics. While differences in residue frequency were evident, no strong correlation was found between frequency and model performance (Fig. 3). Residue-specific model performance varied, with residues such as Threonine (THR), Methionine (MET), and Arginine (ARG) showing higher F1 scores and MCC, indicating a balanced performance in terms of precision and recall. The model tuning process included adjusting the *scale_pos_weight* parameter to address class imbalance, but overall, no significant linear correlation was found between residue frequency and metrics such as F1 Score, MCC, Recall, Precision, or ROC-AUC, with R² values consistently below 0.5.

The scatter plots (Fig. 3) visualize the relationship between residue frequency and performance across different metrics. Residues like Tryptophan (TRP) and Methionine (MET), which are less frequent, exhibited higher F1 scores, while more common residues like LEU and Glutamine (GLN) tended to show lower scores. A similar trend was observed for MCC, where TRP showed a higher MCC, while LEU had a lower MCC despite its higher frequency. Table 3 provides the performance metrics of the residue-based classification models on the test set. Overall, lower-frequency residues like TRP and MET performed better across multiple metrics, whereas higher-frequency residues like LEU showed comparatively lower performance.

### 3.3 STINGAllo Ensemble Model Performance

The STINGAllo ensemble model, driven primarily by CatBoost, showed a clear edge over the other models in predicting AFRs, Supplementary Table S1 and Fig. S2. CatBoost’s strong performance was particularly evident when comparing it to XGBoost and LightGBM. This is largely due to its capacity to capture key features of the internal protein nanoenvironment. The model’s performance was assessed using several metrics, including F1 Score, MCC, Recall, Precision, and ROC-AUC, with results summarized in Table 4. The model produced an F1 Score of 0.64 and an MCC of 0.64 while accessing it on its ability in predicting each residue in the site, indicating a well-balanced prediction of AFRs. It also achieved a high precision score of 0.79, which suggests the model was very effective in correctly identifying AFRs without producing too many false positives. Although the Recall was more modest at 0.54, this balance between precision and recall indicates that CatBoost was particularly adept at making reliable predictions without sacrificing accuracy for sensitivity.

**Table 3:** STINGAllo Performance Metrics.

Metric	Value
F1 Score	0.64
MCC	0.64
Recall	0.54
Precision	0.79
ROC-AUC	0.77

### 3.4 DCC evaluation of AFR prediction accuracy for STINGAllo and PASSer models on the test set

**Fig. 4:**
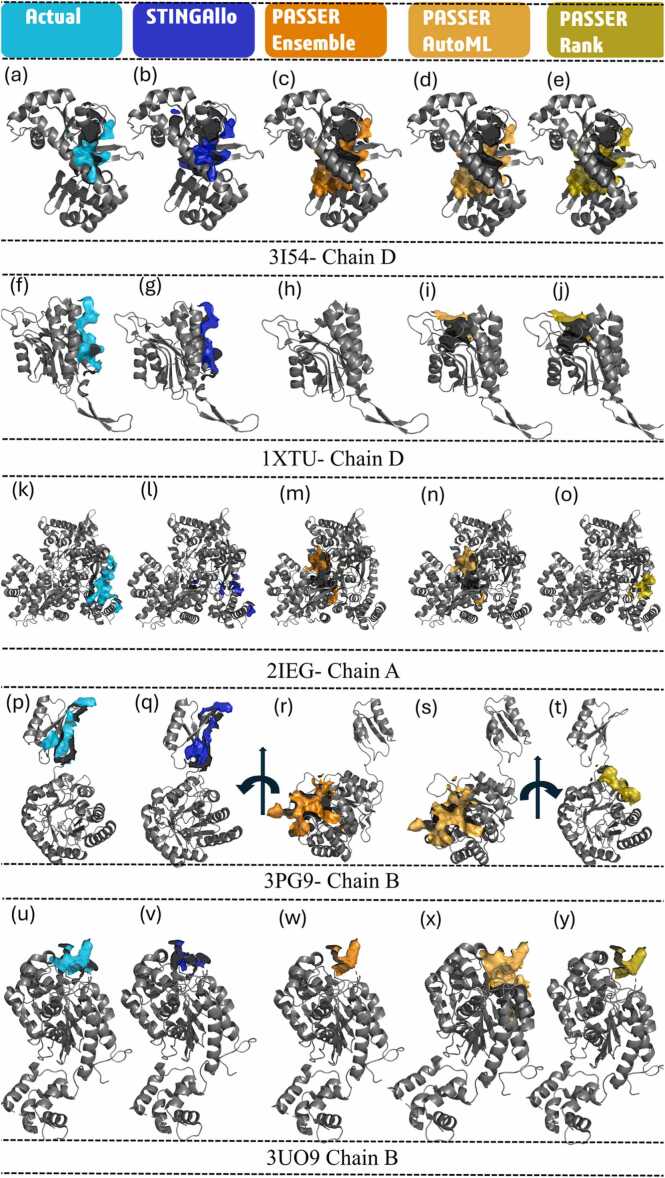
shows the 3D plots of predicted AFRs for the selected proteins by different models. Panels from top left to bottom right represent the following: actual AFRs for panels (a, f, k, p, u) and STINGAllo, PASSer Ensemble, AutoML, and Rank predictions were represented in panels (b, g, l, q, v), (c, h, m, r, w), (d, i, n, s, x), and (e, j, o, t, y), respectively. Cyan for the actual site, blue for STINGAllo, orange for PASSer Ensemble, bright orange for PASSer AutoML and olive for PASSer Rank. The protein cartoon structure is coloured in grey.

The DCC metric was calculated for finding the correctness of prediction in allosteric sites, considering the spatial distance between the predicted AFRs and the actual allosteric sites. Predictions are considered successful when the DCC is ≤ 4 Å. We compare the results from STINGAllo to different model versions: PASSer Ensemble, PASSer AutoML, and PASSer Rank on various proteins and chains. STINGAllo outperformed all the versions of PASSer for all proteins and chains in the test set. More precisely, STINGAllo reached a success rate of 60.23 % in DCC, with most of its predictions inside the acceptable limit of DCC ≤ 4 Å. For all the PASSer models, it was a lot lower; among them, the PASSer Ensemble model gave performances at 21.11 %, PASSer AutoML reached 23.16 %, and the results of PASSer Rank reached 24.21 % (Table S2). We divided the test set into two subsets to allow for a more detailed analysis. The first subset includes instances in which all AFRs are predicted within the pockets detected by FPocket (Table 4). STINGAllo had a success rate of 77.78 % in this subset, outperforming PASSer Ensemble 47.37 %, PASSer AutoML 45.00 %, and PASSer Rank 55.00 %. STINGAllo's superior performance can be attributed to the fact that over 80 % of the ASBench dataset (Table 1) contains instances where the AFRs are within the pocket, providing lots of first subset data for effective learning. The second subset (Table 5) includes instances in which some AFR residues are not predicted by FPocket to belong to the pocket. This gave some hard time to other models, as depicted in Table 5. In this far more challenging subset, STINGAllo maintained a success rate of 55.71 %, again considerably above the performances of PASSer Ensemble at 14.08 %, PASSer AutoML at 17.33 %, and PASSer Rank at 16.00 %. The quite big drop in performances of the PASSer models when FPocket predictions failed to capture all AFRs, mostly in cases where the AFRs are on a flat surface is nicely modelled and visualized in Figs. 4f-j and 4p - t. These results indicate that the STINGAllo model provides more conservative estimates for AFRs compared to the models currently implemented in PASSer.

**Table 4:** DCC Values for STINGAllo and PASSer Models for Proteins with complete AFR belonging to FPocket-Detected Pockets.

PDB Code	Chain	STINGAllo	PASSer Ensemble	PASSer AutoML	PASSer Rank
3ETE	C	0.37	3.82	3.82	3.82
1EFA	B	0.41	3.27	3.27	3.27
2W4I	E	0.63	2.28	2.28	2.28
2Q5O	A	0.92	0.00	21.09	0.00
4AVC	A	1.45	6.46	34.25	34.25
3HQP	C	1.55	39.17	39.17	N/A
1ZDS	B	1.61	1.24	N/A	1.24
2HIM	A	2.03	20.25	20.25	20.25
2PUV	D	2.05	3.09	3.09	5.95
1EFA	C	2.26	N/A	2.20	2.20
4ETZ	A	2.42	1.91	1.91	1.91
3LSF	B	2.44	16.17	12.57	8.99
4HYW	B	2.50	0.45	0.45	0.45
2W4I	B	3.03	N/A	0.97	0.97
3UO9	B	3.12	1.77	10.63	1.77
2HVW	B	7.81	5.22	5.22	16.06
1H9G	A	9.56	33.59	33.59	38.15
4KFB	B	15.10	34.30	23.05	27.30
3L3V	A	19.93	26.39	26.39	11.39
3FUD	A	31.96	37.19	37.19	37.19
4KFB	A	N/A	13.61	13.61	13.61
4EJ8	A	N/A	25.79	1.88	1.88
4I1R	A	N/A	0.53	0.53	0.53

**Table 5:** DCC Values for STINGAllo and PASSer Models for Proteins with the fraction of AFR belonging to FPocket-Detected Pockets.

PDB Code	Chain	STINGAllo	PASSer Ensemble	PASSer AutoML	PASSer Rank
2V4Y	F	0.00	16.49	16.49	16.19
1XXA	B	0.00	10.91	10.91	10.91
3ETG	A	0.00	21.20	12.95	12.95
2ZFZ	B	0.00	9.14	9.14	13.59
2LDB	D	0.00	3.41	3.41	3.41
2ZFZ	F	0.00	13.70	13.70	13.70
1XXA	F	0.00	7.82	7.82	7.82
3ETG	D	0.00	N/A	12.93	12.93
4A2U	F	0.43	1.17	1.17	1.17
2I7N	B	0.60	15.83	15.83	15.83
3GCD	D	0.60	4.94	4.94	13.47
3PTZ	D	0.61	2.38	2.38	2.38
3NWY	E	0.67	20.37	4.64	7.14
3R6S	C	0.92	7.12	7.12	7.12
3PG9	B	0.94	45.26	45.26	22.36
1XTU	C	0.96	18.42	18.42	18.42
1XTU	D	0.96	N/A	18.39	18.39
4MBS	A	1.01	4.92	4.92	32.50
2I7P	C	1.08	13.41	13.41	13.41
3IFA	C	1.26	16.91	16.91	16.91
1G3L	C	1.29	N/A	21.51	21.51
4KZT	Y	1.34	19.97	19.97	19.97
3DBA	B	1.35	1.06	1.06	1.06
1G3L	B	1.46	23.92	3.10	3.10
1B4B	A	1.47	11.86	11.86	11.86
1B4B	B	1.47	15.46	12.62	15.46
3I54	D	1.50	4.79	4.79	4.79
2D60	A	1.53	19.35	19.35	19.14
1B4B	C	1.57	15.88	20.53	15.88
3R6S	A	1.63	9.64	9.64	9.64
4JKT	A	1.79	9.91	9.91	32.18
3NWY	F	1.93	20.23	26.27	20.23
2D5Z	B	2.07	16.89	10.24	10.24
1EM6	B	2.38	29.40	29.40	29.40
1G3L	A	2.41	21.43	21.43	21.43
3S87	A	3.18	2.87	2.87	32.21
3MWB	B	3.48	14.86	14.86	27.12
1F2U	A	3.86	5.77	5.77	5.77
3IRH	B	4.19	9.06	9.06	9.06
4NBN	A	4.21	26.25	13.41	26.25
3N25	D	4.22	31.26	31.26	22.82
1QW7	B	4.28	12.53	N/A	12.53
2QMX	B	5.35	12.57	14.02	14.02
3E3N	B	5.38	14.32	14.32	14.32
2Q8M	B	6.01	17.85	17.85	17.85
1I7S	A	6.48	26.56	13.08	25.22
4BQH	A	7.09	10.71	10.71	10.71
2RDE	A	7.15	2.38	2.38	2.38
2NW8	A	7.53	27.49	27.49	27.49
1PCQ	A	7.70	2.85	2.85	2.85
3QEL	C	8.04	11.12	11.12	39.92
2IEG	A	8.19	27.78	27.78	4.73
1F2U	D	9.43	27.78	27.78	27.78
2NW8	B	10.46	27.38	27.38	27.38
1LVW	C	10.95	29.13	29.13	15.32
2D5X	B	11.71	14.86	11.50	11.04
2WU1	A	11.82	24.82	24.82	24.82
3KGF	B	11.83	25.66	25.66	21.34
3UO9	D	14.20	13.74	32.94	32.94
3BZ7	A	15.74	19.04	19.04	19.04
3VQ8	B	15.93	15.37	11.51	11.51
2YLO	A	18.14	N/A	N/A	N/A
1MP3	A	20.17	29.14	1.34	1.34
4G1N	A	20.87	N/A	1.51	1.51
4LRL	D	21.65	26.90	35.57	23.94
4G1N	D	22.34	2.79	2.79	2.79
2ATS	A	24.90	14.23	14.23	14.23
2BU6	A	26.32	2.34	2.34	2.34
1F2U	C	N/A	5.34	5.34	5.34
2VD3	A	N/A	15.51	33.68	8.21
1CE8	A	N/A	14.90	30.29	14.91
11BG	A	N/A	20.17	20.17	6.41
1H78	A	N/A	32.51	32.51	32.51
3F6G	A	N/A	18.83	18.83	18.83

"N/A" denotes cases where the respective model failed to make a prediction for the specific protein chain. This may occur when the model’s internal algorithms did not identify any suitable AFRs in the given structure or when its prediction confidence fell below a certain threshold, resulting in no output. For the FPocket-based models (PASSer Ensemble, PASSer AutoML, and PASSer Rank), only the top 1 pocket prediction was considered in the analysis.

Indeed, STINGAllo and the PASSer models yielded different results for different proteins, but STINGAllo was always closer to the actual AFRs. For instance, in Protein 3I54 Chain D, STINGAllo succeeded with a DCC of 1.50 Å, while the best of all PASSer models could only reach 4.79 Å (Figs. 4a- 4e). For Protein 1XTU Chain D, the STINGAllo model correctly predicted the allosteric site, located on a flat surface with a DCC of 0.96 Å, whereas the PASSer models either did not predict an allosteric site in case of the ensemble model or resulted in much higher DCC values of 18.39 Å (Figs. 4f–4j), predicting the AFRs inside the available pocket in the chain. For Protein 3PG9 Chain B same case as 1XTU chain D, the performance of the STINGAllo was far superior, at a DCC of 0.94 Å against the predictions of both PASSer Ensemble and PASSer AutoML at 45.26 Å and PASSer Rank at 22.36 Å (Figs. 4k–4o) due to the AFR located on a flat surface outside the purview of FPocket. For Protein 3UO9 Chain B, the STINGAllo was relatively good at a DCC of 3.12 Å. Most importantly, the performance of PASSer Ensemble and PASSer Rank gave similar DCC values, equalling 1.77 Å, whereas for PASSer AutoML it was 10.63 Å. Although in this case, PASSer Rank performs similarly to STINGAllo which is more consistent for varying proteins. The results of the specific protein analyses indeed represent that STINGAllo always predicts AFR with higher precision than PASSer models, as indicated from the low DCC values.

Notice that the STINGAllo predictions in Figs. 4b, 4 g, 4 l, 4q or 4 v are closer to experimental AFRs. The margin increases for more complicated protein structures, where PASSer utterly fails to show precision comparable with STINGAllo. Many times, PASSer Rank outperformed the PASSer Ensemble and AutoML, but in general, it was behind STINGAllo, except for Protein 3UO9 Chain B. That does point out the robustness of STINGAllo over different complexities of proteins. PyMOL session files for AFR visualization and API call scripts for downloading AFR predictions from the PASSer server are also provided in the STINGAllo repository.

**Fig. 5:**
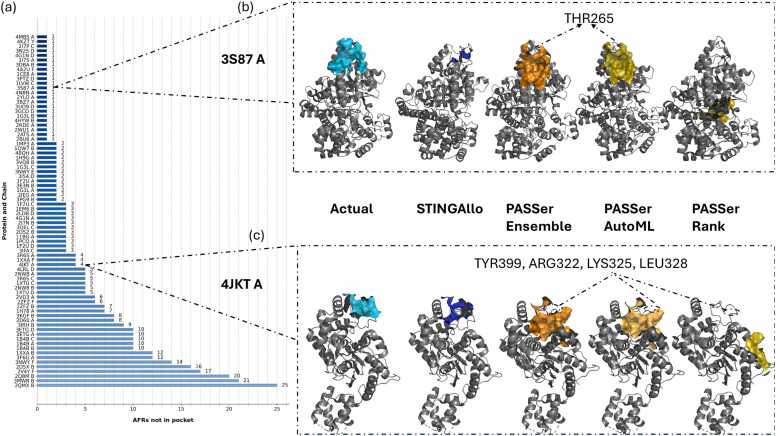
(a) Number of AFRs absent in FPocket predictions for the first subset of the test set. (b) Cartoon representation of protein chains 3S87 A, illustrating THR265 missed by FPocket, with the AFR at close proximity to the correctly predicted pocket for PASSer Ensemble and AutoML and (c) 4JKT A showing the four AFRs missed at close proximity to the predicted pocket matching the actual allosteric site. Cyan for the actual site, blue for STINGAllo, orange for PASSer Ensemble, bright orange for PASSer AutoML and olive for PASSer Rank. The protein cartoon structure is coloured in grey.

In the second subset of the test set, two distinct scenarios were identified where AFRs were located outside the pockets predicted by FPocket. The first scenario is characterized by AFRs positioned on flat protein surfaces, as observed in 1XTU Chain D (Figs. 4f–4j) and 3PG9 Chain B (Figs. 4p–4t). In these cases, the DCC scores for the PASSer models (AutoML, Rank, and Ensemble) were notably high, exceeding 18 Å for 1XTU Chain D and surpassing 45 Å for 3PG9 Chain B. Moreover, PASSer Ensemble did not provide any prediction for 1XTU Chain D. In stark contrast, STINGAllo demonstrated exceptional accuracy in these challenging flat-surface cases, yielding DCC scores below 1 Å for both proteins, thus precisely identifying the AFRs. The second scenario, illustrated in Fig. 5, pertains to instances where FPocket missed some AFRs, but the missed residues remained in close proximity to the predicted allosteric site**.** Fig. 5a shows the number of missed AFRs for all the protein chains in this subset, ranging from 1 missed AFR to as many as 25 missed AFRs, as seen in 2QMX Chain B. In Fig. 5b, 3S87 Chain A exemplifies a case where FPocket missed a single AFR, Thr265. Despite this omission, the missed AFR was still in proximity to other AFRs predicted by the PASSer models, resulting in relatively strong DCC performance. PASSer Ensemble and AutoML both achieved DCC scores of 2.87 Å, while STINGAllo performed similarly, with a DCC of 3.18 Å, successfully predicting the site within 4 Å of the actual allosteric center.

Fig. 5c displays another example, 4JKT Chain A, where four AFRs were located outside FPocket’s predicted pockets. Nevertheless, these AFRs were still spatially close to the predicted active site. Under such conditions, PASSer Ensemble and AutoML yielded DCC scores comparable to STINGAllo, reflecting similar levels of performance when the missed AFRs were near the predicted pocket. PASSer Rank, however, frequently performed poorly in these scenarios, often predicting pockets far removed from the actual allosteric site.

### 3.5 STINGAllo Explanation

**Fig. 6:**
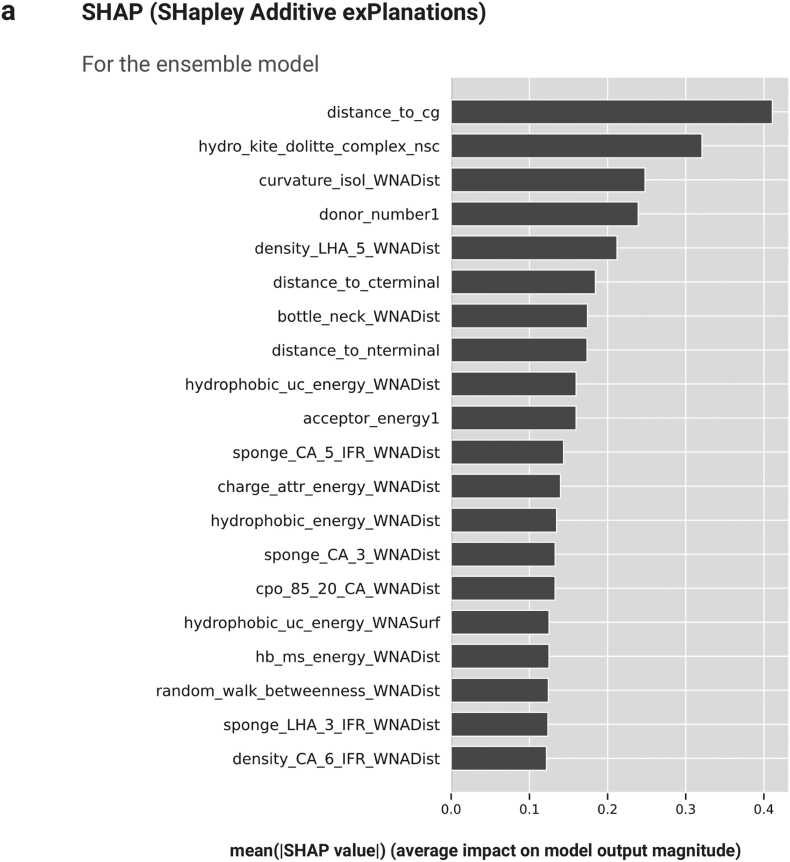

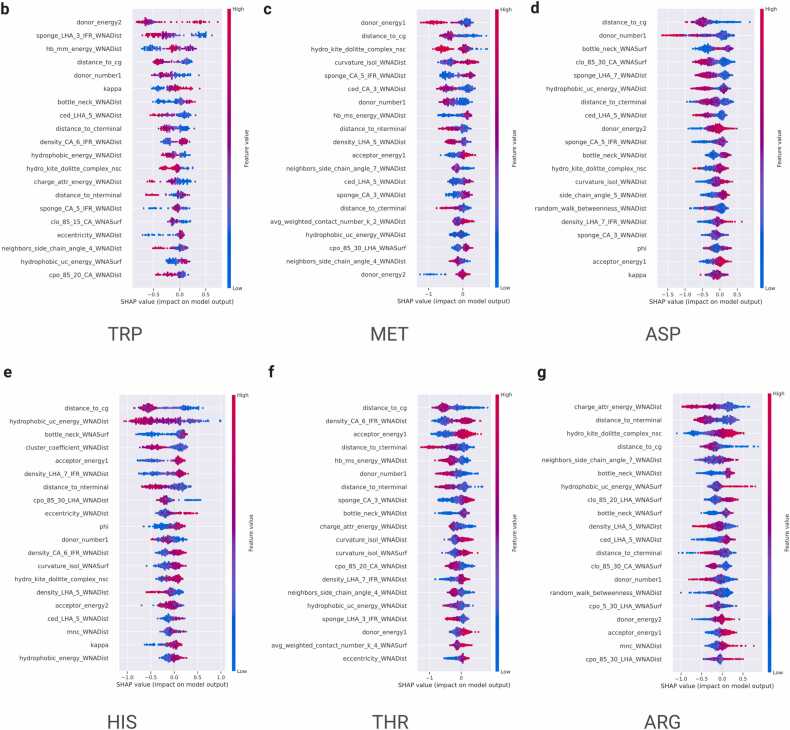
(a) SHAP summary plot showing the mean impact of features across the ensemble of per-residue models.

The ensemble model’s physicochemical descriptor contributions are elucidated through a global SHAP summary plot, as seen in Fig. 6a. The plot demonstrates the averaged impact of each descriptor across the ensemble of AFR residue-specific models, highlighting the ‘distance to the chain’s centre of gravity (cg) descriptor as having the most substantial influence with a mean SHAP value exceeding 0.4. This prominence indicates a pronounced dependency on spatial descriptors, suggesting a pivotal role for geometric features in the model’s ability to differentiate between AFR and FRs. The dominance of spatial features suggests a model that prioritizes structural integrity and spatial configuration in its predictive heuristic. In dissecting the descriptor contributions for individual residue-specific models (best six models), Figs. 6b-g depict the SHAP values for the TRP, MET, ASP, HIS, THR, and ARG models, respectively. Each figure shows a detailed narrative on the importance of individual features, exhibiting a diverse landscape of descriptor contributions specific to the classification of each residue type. The TRP and MET models, which demonstrate high classification precision with F1-scores approaching 0.8, reveal a pronounced significance for the ‘donor energy’ descriptor (Figs. 6b-g).

Fig. 6: (b-g) SHAP value plot for the TRP, MET, ASP, HIS, THR and ARG residue models. In these plots, each dot represents an individual instance and its corresponding SHAP value for a particular feature. Blue dots represent instances with low values for the respective feature, while red dots represent instances with high values. The position of the dots along the x-axis shows the impact of the feature on the model’s output, with values closer to the right indicating a positive impact and values closer to the left indicating a negative impact.

The importance of these descriptors highlights their roles in capturing the properties of residues that influence the model's classification accuracy. The differences in descriptor importance across the models reflect each model's ability to adapt to the unique structural and chemical features of the residues being classified.

## 4 Discussion

The integration of the internal protein nanoenvironment into AFR studies has significantly expanded the scope of AFR identification, surpassing the limitations of molecular surface pocket-focused approaches and previous residue-based methodologies. In training our models, we used the ASBench dataset, which provides experimental annotations of AFRs within protein complexes using LASA. However, there remains the possibility that undiscovered allosteric sites exist within these structures, potentially resulting in some amino acids being mislabelled as FRs rather than AFRs. This misclassification could introduce variability and reduce model accuracy (for all, including our models). Similarly, false positive AFR predictions in the test set might actually correspond to true positives within undiscovered allosteric pockets. Enhanced accuracy will likely follow as more high-resolution protein complex data are annotated and experimentally validated.

Of the eleven already published models cited in the introduction section, nine were trained using pocket information generated from FPocket. The performance metrics of these models assumed that all residues within the predicted pocket are AFRs. However, our comparative analysis of the residues in the ASD 2023 [19] and ASBench [22] datasets revealed that not all predicted residues are located within the predicted pocket (Table 1). This discrepancy can occur either because the protein is not in its fully functional complex form (Single Chains only were considered), leading the program to predict the wrong pocket, or due to the “flatness” of the pocket in some cases[51], causing the program to miss it. Consequently, all models built on this premise inherit these errors, reducing their efficiency in accurately predicting AFRs. We have demonstrated in Table 1, that approximately 18.31 % of the residues in the ASBench dataset do not belong to any pocket predicted by the FPocket program[13] and 55.90 % by the NanoShaper program[50]. This observation highlights a significant limitation in FPocket-based allosteric site prediction methodologies, as it introduces a risk of false negatives (FNs) when relying solely on pocket detection algorithms. These algorithms also often overlook cryptic or non-canonical allosteric sites that do not present a traditional pocket-like curvature, which are increasingly recognized as functionally important in protein regulation[51], [52]. For instance, some allosteric sites may only become functional under specific conformational states or conditions, rendering them invisible to pocket-based methods[53]. STINGAllo algorithm, by contrast, does not depend on FPocket or any pocket prediction tool. Instead, we employ a per-residue prediction approach, which allows us to bypass the limitations of pocket detection altogether. This methodology has resulted in good DCC success rate, F1 scores and MCC values, underscoring the robustness of our approach in identifying AFRs, including possibly those that are cryptic or hidden. For instance, PASSerRank[47] a leading model in allosteric site prediction achieved a DCC of 24.21 % on the ASBench test set while STINGAllo, attained a DCC success rate of 60.23 % (Table 4) and an F1 score of 64 % and an MCC of 64 % (Table 3) on the ASBench test set, predicting each residue.

### 4.1 The critical role of the internal protein nanoenvironment in allosteric regulation

The properties of a protein's internal nanoenvironment offer an intuitive approach to understanding how spatial and physicochemical factors interact to influence amino acid functions, particularly in the context of allosteric regulation. Key factors include the sponge effect, proximity to the protein’s center of geometry (cg), hydrophobic interactions, electrostatic potentials, and structural characteristics, as captured by geometric descriptors like eccentricity and bottleneck features. The sponge descriptor (Figs. 6a-g) calculates the degree of the void spaces around residues and has indeed become integral in most of our predictive models. This measure is indicative of the degree of spatial freedom available to a residue and is essential for the conformational flexibility that must accompany allosteric modulation. By inference, residues that are surrounded by significant areas of 'sponge' must perforce be more capable of participating in the dynamic structural fluctuations underlying allosteric signalling. The distance to cg descriptor thus reflects upon a residue's proximity to the geometric center of the protein, an anchor points per se, reflecting how the position of a residue might affect its potential for allostery considering the overall protein structure. Residues close to or distant from the center of mass differently affect the dynamics and stability of the protein and thus differently affect their propensity to be involved in allosteric communication events and to be defined as AFRs (Fig. 6). In general, a low distance to the center of gravity gives high allosteric potential since the residue can effectively contribute to the protein core architecture and dynamical properties[54], [55], [56]. Hydrophobic interactions play an important role in protein folding and stability, and in general, the hydrophobicity descriptor represents the tendency of the residues to take part in such a non-polar interaction. Other important structural and topological features that characterize proteins are geometric eccentricity and bottleneck. However, while considering electrostatic interactions, descriptors of donor and acceptor energies demarcate charged interactions impinging on the energetic landscape of proteins. Since eccentricity measures the deviation in spatial structure, it, therefore, underlines the residues with a special structural role, while a bottleneck descriptor is a method of identifying critical nodes in a protein structure. These feature some important residues for the integrity of the structure and some for allosteric communication. Such descriptors are important features in understanding the internal nanoenvironment of proteins and extend our ability in the precise prediction of AFRs, giving strategic insights into drug development. The identification of residues with favourable spatial, energetic, and structural properties for allosteric modulation may provide important guidance in the design of allosteric modulators. The properties of internal protein nanoenvironment could be considered as multidimensional features presented across the spatial, energetic, and structural axes as responsible for determination of the protein residue allosteric propensities. One might consult several general overview parts with this regard, and that are described in Supplementary below [57].

### 4.2 Implications for drug design and therapeutic development

The ability to identify AFRs within a large array of protein structures heralds a new era in the rational design of drugs having high specificity and efficiency in the modulation of protein functions. Therapeutic intervention at allosteric sites has several key advantages compared with targeting active or orthosteric sites. This is partly because allosteric sites are generally less conserved across protein families than orthosteric sites, decreasing the possibility of cross-reactivity and thereby reducing off-target effects. This specificity is particularly critical for the design of drugs with a favourable safety profile. Allosteric modulators may provide a more subtle way of regulating protein activity, even down to the degree of being able to fine-tune rather than fully inhibit or activate a protein function.

STINGAllo, although promising, is only one tool within the armamentarium for drug discovery, far from being a final answer. Its strength relies on the capability of defining the residues that form allosteric sites by using internal protein nanoenvironment descriptors of proteins: a new dimension of understanding that will be contributing to the finding of novel allosteric sites. Note that the descriptors used are derived from bound protein structure, and these may not truly represent the real apo states due to the induced conformational changes which are going to be induced upon the binding of modulators. Another limitation is that, in general, bound structures are dependent on the fluctuating and sometimes altered contexts of such residues that could not be fully representative of the unbound state, thus distorting the transferability in the prediction of long-range allostery among the different states. Given the present F1 score of 0.64 and the intrinsic bias present in the dataset, predictions from STINGAllo and any other allosteric site prediction models should be treated as probabilities rather than as an absolute prediction. These implications of the use of bound state structures would therefore suggest that while STINGAllo was able to predict probable allosteric residues, such predictions may not fully capture the real extent of conformation flexibility or in vivo allosteric signalling pathways. This is a limitation that may well be overcome with further refinement of the model, including unbound structure or dynamic simulations such as molecular dynamics, which can express the apo-states and conformational shifts related to allosteric regulation more accurately.

## 5 Conclusions

Our findings highlight the critical role of the internal protein nanoenvironment in determining the allosteric potential of residues, emphasizing the importance of spatial and physicochemical context in allosteric regulation. By integrating a per residue-based approach, our STINGAllo model overcomes the limitations of traditional pocket-focused methodologies, providing more accurate predictions of AFRs with overall DCC success rate of 60.23 % and 77.78 % when all AFRs were predicted within pockets identified by FPocket. The model’s less sensitivity to pocket feature and its capability to handle cryptic allosteric sites, particularly those that are not part of a pocket, further enhance its utility.

The implications of the STINGAllo model for drug design are substantial. By accurately mapping allosteric sites and providing insights into the molecular determinants of allosteric regulation, the model opens new avenues for the development of allosteric modulators. We have integrated the key descriptors defining allosteric internal protein nanoenvironment into the Dictionary of Internal Protein Nanoenvironment (DIPN), hosted by the Brazilian Agricultural Research Cooperation (EMBRAPA) at https://www.proteinnanoenvironments.cnptia.embrapa.br/.

## Author contributions

Conceptualization, G.N., I.M., J.A.S., L.B., F.R.dM., and I.Y.; methodology, F.B.O.; software, F.B.O.; validation, F.B.O., G.N., and I.M.; formal analysis, F.B.O.; investigation, F.B.O.; resources, G.N., I.M., I.Y., J.A.S., and L.B.; data curation, F.B.O.; writing—original draft preparation, F.B.O.; writing—review and editing, F.B.O., G.N., I.M., I.Y., J.A.S., L.B., L.T., J.H.F.G., P.F.G., F.R.dM., and R.K.A.; visualization, F.B.O.; supervision, G.N., L.T., P.F.G., and R.K.A.; project administration, G.N.; funding acquisition, R.K.A. All authors have read and agreed to the published version of the manuscript.

## Funding

This research was funded by Sao Paulo Research Foundation (Fundacão de Amparo à Pesquisa do Estado de São Paulo - FAPESP), Grant numbers 2023/02691-2 and 2020/08615-8. J.E.H.G. would also like to thank FAPESP and CNPq (Grant numbers 2024/13327-2 and 401415/2023-6).

## CRediT authorship contribution statement

**Folorunsho Bright Omage:** Writing – original draft, Visualization, Validation, Software, Methodology, Investigation, Formal analysis, Data curation. **José Augusto Salim:** Writing – review & editing, Data curation. **Ivan Mazoni:** Writing – review & editing, Resources, Data curation. **Inácio Henrique Yano:** Resources, Data curation. **Luiz Borro:** Data curation. **Jorge Enrique Hernández Gonzalez:** Writing – review & editing. **Fabio Rogerio de Moraes:** Data curation. **Poliana Fernanda Giachetto:** Resources, Project administration. **Ljubica Tasic:** Writing – review & editing, Supervision, Funding acquisition. **Raghuvir Krishnaswamy Arni:** Writing – review & editing, Supervision, Funding acquisition. **Goran Neshich:** Writing – review & editing, Writing – original draft, Visualization, Validation, Supervision, Software, Resources, Project administration, Methodology, Investigation, Funding acquisition, Formal analysis, Data curation, Conceptualization.

## Declaration of Competing Interest

The authors declare that they have no known competing financial interests or personal relationships that could have appeared to influence the work reported in this paper.

## Data Availability

In alignment with the journal commitment to open research, we have made the notes supporting the findings of this study accessible. The supplementary data is included in the Zonodo’s links. Additionally, the scripts and codes utilized in our analysis are available on our GitHub repository at https://github.com/omagebright/ Exosite-STING-ML-Prediction.

## Footnotes

Appendix A Supplementary data associated with this article can be found in the online version at doi:10.1016/j.csbj.2024.10.036.
