# Supplementary material for: Protein allosteric site identification using machine learning and per amino acid residue reported internal protein nanoenvironment descriptors
Source: Comput Struct Biotechnol J. 2024 Oct 23;23:3907–19. doi: 10.1016/j.csbj.2024.10.036 (PMC11570862; doi:10.1016/j.csbj.2024.10.036)
Supplement: Supplementary file 2 — Supplementary material [file mmc2.pdf]

Table 1: Overview of selected physicochemical descriptors extracted from the STING-RDB2 database for model development.
Folorunsho Bright Omage

**Descriptor Class**	**Descriptors**	**Description**
Accessibility	acc_ifr_nsc, bsa_ratio_naccess	The STING-RDB2 employs the rolling sphere algorithm, as initially proposed by Lee and Richards, 1971[**?**], to accurately compute the accessible surface area (ASA) of proteins. For the model development, we focused on two pivotal accessibility descriptors: *(i)* The Interface Forming Residues (IFR), which delineates the ASA in isolation from that in a complex, with residues exhibiting a lower IFR compared to their isolated state being integral to the protein interface. *(ii)* The Buried Surface Area (BSA), a critical parameter for gauging the interface size within a complex, is defined by the equation: $$\text{BSA} = \frac{\text{ASA}_{\text{IFR}}}{\text{ASA}_{\text{isolated}}} \qquad (1)$$ Here, ASA_{IFR} denotes the accessible area of a residue at the interface, and $\text{ASA}_{\text{isolated}}$ represents the residue's accessible area in isolation. The computation of BSA and IFR, is by NACCESS© [**?**].

Table 1 continued from previous page

Descriptor Class	Descriptors	Description
Cross_Link_Order_WNA	clo_35_15_CB_WNASurf, clo_35_30_LHA_WNASurf, clo_85_15_CA_WNASurf, clo_85_20_CB_WNASurf, clo_85_20_LHA_WNASurf, clo_85_30_CA_WNASurf, clo_35_30_CA_WNADist, clo_35_30_CB_WNADist, clo_35_30_LHA_WNADist, cpo_35_30_LHA_WNASurf, cpo_5_30_LHA_WNASurf, cpo_85_20_CA_WNASurf, cpo_85_30_LHA_WNASurf, cpo_35_30_LHA_WNADist, cpo_85_20_CA_WNADist, cpo_85_30_LHA_WNADist	Cross-Links (CLO) are contacts established among residues that are distant in the primary sequence yet proximal in the protein's three-dimensional fold. The *order of cross-link* refers to the number of contacts made among discrete segments of the protein sequence, with segments varying in size from 15, 20, to 30 amino acids. For a given amino acid residue, even if multiple contacts target the same sequence stretch, only a single occurrence is counted for the Cross Link Order. This means that a central amino acid may establish more than one contact within a targeted sequence stretch, each potentially with a different amino acid within the same probing sequence length (15, 20, or 30 amino acids).<br>The importance of a residue for protein folding, stability, or binding is suggested to be higher with an increasing order of cross-link. The selected CLO descriptors are calculated by varying three parameters:<br>• The length of the sequence stretch separating the residues in contact (15, 20, or 30 amino acids).<br>• The radius size of the probing sphere, within which the contacts are evaluated (3.5, 5, or 8.5 Å).<br>• The centre of the probing sphere, which can be either the C-α, C-β, or the Last Heavy Atom (LHA) in the side chain.<br>STING-RDB2 contain several CLO descriptors, including variations with the probe sphere centred at different atoms (C-α, C-β, LHA) and considering different stretch lengths and radii. The selected few for this study are categorized below:<br>1. CLO descriptors with the probe sphere centered at C-α for three different stretch lengths and radii are denoted as clo_35_15_CA, clo_5_15_CA, clo_85_15_CA, etc.<br>2. CLO descriptors with the probe sphere centered at C-β are denoted as clo_35_15_CB, clo_5_15_CB, clo_85_15_CB, etc.<br>3. CLO descriptors with the probe sphere centred at the LHA are denoted as clo_35_15_LHA, clo_5_15_LHA, clo_85_15_LHA, etc.<br>Each descriptor captures important physicochemical information, which we leverage to delineate AFR and FRs at per residue level.

Table 1 continued from previous page

Descriptor Class	Descriptors	Description
Curvature WNA	curvature isol WNASurf, curvature complex WNASurf, cavity curvature isol WNASurf, cavity msa complex WNASurf, curvature isol WNADist, curvature complex WNADist, cavity curvature isol WNADist, cavity asa isol WNADist, cavity asa complex WNADist, cavity msa complex WNADist	The curvature for each amino acid is determined utilizing the Surface Racer program [**?**], which computes curvature values at the atomic level. Initially, each atom on the protein's surface is assigned a curvature value indicative of its spatial context: negative for concave regions, positive for convex regions, and zero for buried atoms not exposed on the surface. These values are then averaged for the atoms that constitute an amino acid to define the residue's curvature. Blue Star STING further refines this by calculating the mean curvature for surface-exposed residues only, excluding any atom with a curvature of zero. This curvature is computed for the protein both as an isolated chain and when complexed with other chains, if applicable.<br>For the model, these were the curvature descriptors retrieved from the database:<br>• Curvature in isolation (WNASurf)<br>• Curvature in complex (WNASurf)<br>• Cavity curvature in isolation (WNASurf)<br>• Cavity MSA in complex (WNASurf)<br>• Curvature in isolation (WNADist)<br>• Curvature in complex (WNADist)<br>• Cavity curvature in isolation (WNADist)<br>• Cavity ASA in isolation (WNADist)<br>• Cavity ASA in complex (WNADist)<br>• Cavity MSA in complex (WNADist)
DSSP	kappa, phi, acceptor energy1, acceptor energy2, donor energy1, donor energy2, donor number1, fst bridge pair number, snd bridge pair number	Dihedral angles and energy calculations related to protein secondary structure as defined by DSSP (Dictionary of Protein Secondary Structure).
Density_Sponge_WNA	density LHA_5 WNADist, density CA_6 IFR WNADist, density LHA_7 IFR WNADist, sponge CA_3 WNADist, sponge LHA_7 WNADist, sponge CA_5 IFR WNADist, sponge LHA_3 IFR WNADist	Density and 'sponge' metrics provide insight into the spatial distribution and packing of amino acids within protein structures.
Distances	distance to cterminal, distance to nterminal, distance to cg	Metrics assessing the spatial proximity of amino acids to key structural features of proteins, such as the N-terminal, C-terminal, and the center of geometry.
Energy_Density_WNA	ced_CA_7_WNASurf, ced_CA_3_WNADist, ced_LHA_5_WNADist	Energy density measurements based on weighted neighborhood analysis (WNA) to evaluate the energetic landscape surrounding amino acids in proteins.

Table 1 continued from previous page

Descriptor Class	Descriptors	Description
Entropy_Density_WNA	entd_LHA_3_WNADist	Entropy density metrics using weighted neighborhood analysis to assess the disorder and distribution of energy states within protein structures.
Graph_Descriptor_WNA	dice similarity WNASurf, dmnc WNASurf, degree WNASurf, bottle neck WNASurf, eccentricity WNADist, mnc WNADist, closeness WNADist, cluster coefficient WNADist, random walk betweenness WNADist, bottle neck WNADist	Graph-theoretical descriptors derived from weighted neighborhood analysis to characterize the topological and geometric properties of protein structures.
Hydrophobicity	hydro_kite_dolitte_complex_nsc	A measure of the hydrophobic character of amino acids, impacting protein folding and stability.

Table 1 continued from previous page

Descriptor Class	Descriptors	Description
Residue_Contacts_WNA	hb_ms_energy_WNASurf, hb_ss_energy_WNASurf, hydrophobic_energy_WNADist, charge_attr_energy_WNADist, hb_mm_energy_WNADist, hb_ms_energy_WNADist, aromatic_energy_WNADist, hb_ss_energy_WNADist	The intricate nature of protein interactions is characterized by various atomic contacts, which are fundamental to the structural integrity and functionality of proteins and their complexes. The methodology for calculating residue-residue contacts is shown in the work of Mancini et al.[**?**]. The STING database database categorizes contact types based on their interaction energy. For the development of the predictive model, the following STING descriptors were selected to encapsulate the aforementioned interactions within the nanoenvironment of the protein:<br>**Interaction Energies for Residue-Residue Contacts:**<br>**Hydrophobic Interactions:** Hydrophobic, 0.6 Kcal/mol.<br>**Hydrogen Bonding:** Polar, 2.6 Kcal/mol.<br>**Aromatic Stacking:** π-π Stacking, 1.5 Kcal/mol.<br>**Salt Bridging:** Ionic, 10.0 Kcal/mol.<br>**Cysteine-Bridging:** Covalent, 85.0 Kcal/mol.<br>**Selected STING Descriptors:**<br>• `hb_ms_energy_WNASurf` – Energy of main-chain to side-chain hydrogen bonds on the surface.<br>• `hb_ss_energy_WNASurf` – Energy of side-chain to side-chain hydrogen bonds on the surface.<br>• `hydrophobic_energy_WNADist` – Energy of hydrophobic interactions based on distance.<br>• `charge_attr_energy_WNADist` – Energy of charge attraction based on distance.<br>• `hb_mm_energy_WNADist` – Energy of main-chain to main-chain hydrogen bonds based on distance.<br>• `hb_ms_energy_WNADist` – Energy of main-chain to side-chain hydrogen bonds based on distance.<br>• `aromatic_energy_WNADist` – Energy of aromatic stacking interactions based on distance.<br>• `hb_ss_energy_WNADist` – Energy of side-chain to side-chain hydrogen bonds based on distance.
Rotamer	phi	Rotational isomer metrics, specifically the phi angle, which is crucial for understanding the conformational dynamics of amino acid side chains in proteins.

Table 1 continued from previous page

Descriptor Class	Descriptors	Description
Side Chain Orientation WNA	side chain angle_5 WNADist, neighbors side chain angle_4 WNADist, neighbors side chain angle_7 WNADist	The side chain orientation is quantified for each amino acid residue within a protein chain by computing the angle between two vectors: $C\alpha\text{-}\vec{CENTROID}$ and $C\alpha\text{-}\vec{LHA}$. The vector $C\alpha\text{-}\vec{CENTROID}$ is defined from the $C\alpha$ atom of an amino acid residue to the centroid of a specific region (probe sphere). Conversely, $C\alpha\text{-}\vec{LHA}$ is the vector from the $C\alpha$ atom to the Last Heavy Atom (LHA) of the same amino acid residue. For each residue, the deviation of their side chains ($C\alpha\text{-}\vec{LHA}$) from the vector pointing towards the probe sphere's centroid is calculated. Moreover, the average angle for all amino acid residues within the probe sphere is determined. Subsequently, this average angle is subtracted from the individual residue's angle, yielding a measure of how each residue's side chain orientation diverges or converges relative to its neighbors. For the development of the model, selected side chain orientation descriptors include:<br>• Side chain angle with 5Å weighted neighbor average distance (WNADist),<br>• Neighbor's side chain angle with 4Å WNADist,<br>• Neighbor's side chain angle with 7Å WNADist.
Stride	phi, accessibility	Metrics including dihedral angles and solvent accessibility, providing insight into the secondary structure and surface characteristics of proteins.
Unused_Contacts_WNA	hydrophobic uc energy WNASurf, hydrophobic uc energy WNADist	Each amino acid residue has the potential to establish a certain number of interatomic contacts. The concept of *Unused Contacts* is defined by the discrepancy between the maximum feasible contacts and the actual contacts formed. Contacts within this framework are categorized based on the type of contact and residue type. Only those contacts derived from Protein Data Bank (PDB) structures determined by X-ray crystallography with a resolution of 2.0 Å or better are considered. Structures exhibiting double occupancy atoms are explicitly excluded from this analysis.

Table 1 continued from previous page

Descriptor Class	Descriptors	Description
Weighted Contact Number WNA	avg weighted contact number k2 WNASurf, avg weighted contact_number k4 WNASurf, avg weighted contact number k2 WNADist	The Weighted Contact Number (WCN) is a descriptor of the backbone flexibility of amino acid residues [**?**]. The WCN for each residue in the protein sequence is calculated by summing the inverse square distances from the residue of interest to all other residues in the protein. The WCN for residue i, denoted as WCN_i, is computed using the equation: $$WCN_i = \sum_{j \neq i} \frac{1}{r_{ij}^2} \quad (2)$$ where j represents any residue in the protein other than residue i, and r_{ij}^2 is the square of the Euclidean distance between the α-carbons of residues i and j. The average WCN for a residue is determined based on its k nearest neighbors as follows: $$\overline{WCN}_i = \sum_{j \in \mathcal{K}_i} \frac{z_j}{K} \quad (3)$$ where $\mathcal{K}_i$ denotes the set of k nearest neighbors to residue i (based on the square Euclidean distance between α-carbons), z_j is the normalized WCN (z-score) of the neighbor j, and $K = \|\mathcal{K}_i\|$ is the number of nearest neighbors (up to a maximum of k). In this study, we utilized various WCN descriptors, including: • average weighted contact number ($k = 2$) on accessible surface (*avg_weighted_contact_number_k2_WNASurf*), • average weighted contact number ($k = 4$) on accessible surface (*avg_weighted_contact_number_k4_WNASurf*), • average weighted contact number ($k = 2$) on distance-based metric (*avg_weighted_contact_number_k2_WNADist*).
