# Supplementary material for: Protein allosteric site identification using machine learning and per amino acid residue reported internal protein nanoenvironment descriptors
Source: Comput Struct Biotechnol J. 2024 Oct 23;23:3907–19. doi: 10.1016/j.csbj.2024.10.036 (PMC11570862; doi:10.1016/j.csbj.2024.10.036)
Supplement: Supplementary file 4 — Supplementary material [file mmc4.pdf]

Protein allosteric site identification using machine learning and per amino acid residue reported internal protein nanoenvironment descriptors

Folorunsho Bright Omage[a,c], José Augusto Salim[b], Ivan Mazoni[a], Inácio Henrique Yano[a], Luiz Borro[a], Jorge Enrique Hernández Gonzalez[d], Fabio Rogerio de Moraes[a], Poliana Fernanda Giachetto[a], Ljubica Tasić[c], Raghuvir Krishnaswamy Arni[d], Goran Neshich[a,1,*]

[a]*Computational Biology Research Group, Embrapa Digital Agriculture, Campinas, São Paulo, Brazil*
[b]*Department of Plant Biology, Institute of Biology, University of Campinas (UNICAMP), Campinas, São Paulo, Brazil*
[c]*Biological Chemistry Laboratory, Department of Organic Chemistry, Institute of Chemistry, University of Campinas (UNICAMP), Campinas, São Paulo, Brazil*
[d]*Department of Physics, Multi-User Center for Biomolecular Innovation, State University of São Paulo (UNESP), São Paulo 05315-970, Brazil*

1. Supplementary-Methodology

1.1. Evaluation Metrics for Model Performance

To thoroughly assess the performance of our predictive models, we employed a suite of evaluation metrics, each providing unique insights into different aspects of model accuracy and reliability. These metrics are derived from the fundamental components of a confusion matrix: true positives (TP), true negatives (TN), false positives (FP), and false negatives (FN). Metrics detail

1.1.1. Accuracy

Accuracy represents the proportion of true results (both true positives and true negatives) among the total number of cases examined. It is defined as:

$$\text{Accuracy} = \frac{TP + TN}{TP + FP + TN + FN}$$

1.1.2. Recall

Recall, also known as sensitivity, measures the proportion of actual positives that are correctly identified by the model.

$$\text{Recall} = \frac{TP}{TP + FN}$$

1.1.3. Precision

Precision assesses the proportion of positive identifications that were actually correct. Precision is calculated as:

$$\text{Precision} = \frac{TP}{TP + FP}$$

1.1.4. Matthews Correlation Coefficient (MCC)

The Matthews Correlation Coefficient is a more robust metric that takes into account all four categories of the confusion matrix, providing a balanced measure of model performance even when class sizes are imbalanced. MCC is given by:

$$\text{MCC} = \frac{(TP \times TN) - (FP \times FN)}{\sqrt{(TP + FP) \times (TP + FN) \times (TN + FP) \times (TN + FN)}}$$

1.1.5. F1 Score

The F1 score is the harmonic mean of precision and recall, offering a balance between the two metrics. It is particularly useful when the class distribution is uneven. The F1 score is defined as:

$$\text{F1 Score} = 2 \times \frac{\text{Precision} \times \text{Recall}}{\text{Precision} + \text{Recall}}$$

1.1.6. Receiver Operating Characteristic (ROC) and Area Under the Curve (AUC)

ROC analysis is a graphical representation of a model's diagnostic ability, plotting the true positive rate (recall) against the false positive rate (1 - specificity) at various threshold settings. The area under the ROC curve (AUC) provides a single measure of overall model performance across all classification thresholds. The closer the AUC is to 1, the better the model is at distinguishing between the positive and negative classes.

1.2. SHAP Analysis

In this study, we employed SHAP (SHapley Additive exPlanations), a game theory-based approach, to explain the output of STING-AFR models. This methodology enables the interpretation of the effect of each physicochemical descriptor on the model's prediction for individual predictions, thus providing insights into both the ensemble and individual model's behaviour. We instantiate a SHAP Tree explainer for each loaded model. The explainer was

*Corresponding author
Email address: goran.neshich@embrapa.br (Goran Neshich)
[1]Current address: Computational Biology Research Group, Embrapa Agricultural Informatics, Campinas, São Paulo, Brazil.

then used to compute SHAP values for the selected instances, which quantify the contribution of each feature to the model's prediction.

To elucidate the individual contributions of descriptors to the prediction of specific instances, we employed waterfall plots. These plots provide a step-by-step decomposition of how each descriptor's SHAP value influences the model's output for a given instance, starting from a base value. The base value represents the model's average prediction across all data points, and each step in the waterfall plot reflects the incremental impact of a descriptor, either positively (red) or negatively (blue) adjusting the prediction from this baseline. This detailed visualization allows for an in-depth analysis of the predictive dynamics at play for individual instances, highlighting the specific descriptors that are most influential in determining the allosteric potential of a residue.
