# Supplementary material for: Protein allosteric site identification using machine learning and per amino acid residue reported internal protein nanoenvironment descriptors
Source: Comput Struct Biotechnol J. 2024 Oct 23;23:3907–19. doi: 10.1016/j.csbj.2024.10.036 (PMC11570862; doi:10.1016/j.csbj.2024.10.036)
Supplement: Supplementary file 5 — Supplementary material [file mmc5.pdf]

Algorithm 1 Residue-Based Classification Algorithm

Require: X_train_scaled, y_train, X_val_scaled, y_val, Classifier (`CatBoost`, `XGBoost`, `LightGBM`)

$residue\_names \leftarrow$ `unique`$(X\_train\_scaled['residue\_name'])$
$metrics\_list \leftarrow []$ **for** $residue\_name\ in\ residue\_names$ **do**
$X\_train\_residue \leftarrow X\_train\_scaled[X\_train\_scaled['residue\_name'] == residue\_name].drop('residue\_name', axis = 1)$
$y\_train\_residue \leftarrow y\_train[X\_train\_scaled['residue\_name'] == residue\_name]$
$count\_neg, count\_pos \leftarrow$ `bincount`$(y\_train\_residue)$
$scale\_pos\_weight \leftarrow count\_neg/count\_pos$
$model \leftarrow$ `Classifier`$(scale\_pos\_weight = scale\_pos\_weight, verbose = 0)$
$model.fit(X\_train\_residue, y\_train\_residue)$
$model\_filename \leftarrow f'../models/A - Residue - V4/\{residue\_name\}\_model\_catboost.joblib'$
`joblib.dump`(model, $model\_filename$)
$loaded\_model \leftarrow$ `joblib.load`$(model\_filename)$
$X\_val\_residue \leftarrow X\_val\_scaled[X\_val\_scaled['residue\_name'] == residue\_name].drop('residue\_name', axis = 1)$
$y\_val\_residue \leftarrow y\_val[X\_val\_scaled['residue\_name'] == residue\_name]$
$y\_pred \leftarrow loaded\_model.predict(X\_val\_residue)$
$f1 \leftarrow$ `f1_score`$(y\_val\_residue, y\_pred)$
$mcc \leftarrow$ `matthews_corrcoef`$(y\_val\_residue, y\_pred)$
$recall \leftarrow$ `recall_score`$(y\_val\_residue, y\_pred)$
$precision \leftarrow$ `precision_score`$(y\_val\_residue, y\_pred)$
$rocauc \leftarrow$ `roc_auc_score`$(y\_val\_residue, y\_pred)$
$metrics\_list.append(\{'Residue' : residue\_name, 'F1' : f1, 'MCC' : mcc, 'Recall' : recall, 'Precision' : precision, 'ROC - AUC' : rocauc\})$

$sorted\_metrics \leftarrow$ `sorted`$(metrics\_list, key = lambdax : x['F1'], reverse = True)$
