# Supplementary material for: Protein allosteric site identification using machine learning and per amino acid residue reported internal protein nanoenvironment descriptors
Source: Comput Struct Biotechnol J. 2024 Oct 23;23:3907–19. doi: 10.1016/j.csbj.2024.10.036 (PMC11570862; doi:10.1016/j.csbj.2024.10.036)
Supplement: Supplementary file 6 — Supplementary material [file mmc6.pdf]

0.0.1. *Geometric Eccentricity and Bottleneck Descriptors*

In Tryptophan (TRP) model, SHAP values underscored the importance of *geometric eccentricity* and *bottleneck* descriptors. Geometric eccentricity, a descriptor derived from graph-theoretical analysis, quantifies the extent to which a residue's spatial positioning deviates from conventional geometric forms. This deviation is indicative of how uniquely a residue is embedded within the protein's three-dimensional architecture. A higher eccentricity value suggests that the residue occupies a spatial niche that may be critical for the protein's conformational dynamics. Such residues, by virtue of their unique positioning, could serve as pivotal points for transmitting allosteric signals across the protein structure, making them prime targets for allosteric modulation. The bottleneck descriptor, on the other hand, illuminates residues that act as critical nodes within the protein's interaction network. These residues, often situated in regions where multiple structural pathways converge, can significantly influence the protein's overall stability and functionality. Alterations to bottleneck residues, whether through mutation, post-translational modifications, or ligand binding, can propagate substantial structural rearrangements, thereby modulating the protein's activity.

Geometric eccentricity and bottleneck descriptors in our predictive models, as highlighted by the SHAP analysis and visualized in the waterfall plots, offers a revealing approach to identifying AFRs. With the model focusing on whether the residue possesses unique spatial attributes and also holds central roles within the protein's structural network.

0.0.2. *Donor and Acceptor Energy Metrics*

Hydrogen bonds, characterized by their directionality and strength, are pivotal in maintaining the intricate three-dimensional structures of proteins. The *donor energy* metric encapsulates the energy potential of residues capable of donating a hydrogen bond, essentially quantifying their propensity to engage in stabilizing interactions within the protein matrix. Similarly, the *acceptor energy* metric captures the potential energy of residues poised to accept hydrogen bonds, further cementing their role in the structural cohesion and integrity of protein complexes. The incorporation of donor and acceptor energy metrics into our models highlights the nuanced contribution of hydrogen bonding to AFR prediction. Allosteric modulation often entails subtle yet significant conformational shifts within protein structures, with hydrogen bonds acting as the molecular levers that facilitate these transformations. Residues with high donor or acceptor energy potentials are likely to be key players in the allosteric mechanism, serving as hinge points that relay conformational changes from the allosteric site to the active site, or vice versa.

0.0.3. *Sponge Descriptor: Emphasizing Spatial Context*

Among the array of physicochemical descriptors analyzed, the *sponge* descriptor stands out for its unique ability to capture the essence of the spatial context surrounding each residue within its nano-environment. This descriptor, by quantifying the void or empty spaces around residues, serves as a critical indicator of the structural flexibility inherent to protein architectures. The sponge descriptor operationalizes the concept of spatial freedom for residues, a factor that is pivotal in facilitating the conformational adaptability required for allosteric regulation. The computation of the sponge descriptor involves aggregating the van der Waals volumes of all atoms within a defined radius around a residue's alpha carbon (CA) or the Last Heavy Atom (LHA) in its side chain. This aggregate volume is then normalized by the total volume of the spherical region considered, providing a measure of how densely atoms are packed around the residue:

$$\text{Sponge}_{\text{residue}} = \frac{\sum \text{Van der Waals volume of atoms within sphere}}{\text{Volume of sphere}} \tag{1}$$

This metric illuminates the 'breathing room' available to each residue, which directly impacts its potential to undergo or contribute to allosteric transitions or form AFR.

The presence of significant 'sponge' areas around residues implies the existence of pathways that can accommodate or even facilitate the propagation of allosteric signals. These pathways, formed by the interstitial voids, allow for the dynamic rearrangements of atomic positions essential for transmitting allosteric effects from the modulator site to the functional site of the protein. In essence, the sponge descriptor sheds light on the structural conduits through which allosteric signalling can traverse.

0.0.4. *Spatial Orientation and Density Metrics*

Metrics related to *spatial orientation* and *density* further highlight the importance of the three-dimensional arrangement and the compactness of residues' surroundings in their functional roles within proteins. Spatial orientation metrics delve into the angular disposition of residues in relation to one another and the protein's geometric center. This orientation is crucial in defining the residue's potential to participate in or propagate allosteric signals, as the directionality of interactions often governs the efficiency of signal transmission. For instance, residues that are aligned in a manner conducive to forming or disrupting key interaction networks upon conformational shifts are likely pivotal in the allosteric process.

Density metrics, on the other hand, quantify the compactness of residues' surroundings, providing an estimate of how closely packed the amino acids are within a given spatial domain. High-density areas might indicate regions of structural stability or rigidity, whereas lower density could signify regions more amenable to conformational flexibility. The balance between stability and flexibility is a cornerstone of allosteric regulation, as it ensures the protein's structural integrity while allowing for the dynamic changes essential for allosteric modulation.

0.0.5. Descriptor Contributions

The utilization of waterfall plots, Figure **??** in our SHAP analysis offers a compelling visualization of how individual descriptors influence the predictive outcome of our models, either augmenting or diminishing the base prediction for a given residue's allosteric potential. These plots provide a step-by-step breakdown of the prediction process, starting from a base value that represents the average model output across the dataset, and cumulatively adding the impact of each descriptor to arrive at the final prediction for a specific instance. The base prediction serves as the foundation upon which the effects of individual descriptors are layered. This value reflects the model's average expectation for the allosteric potential of residues before considering the unique characteristics of any specific residue. It provides a neutral benchmark, enabling a clear assessment of how each descriptor's contribution sways the prediction away from this average.

Descriptors that contribute positively to the model's prediction are those that enhance a residue's likelihood of being an AFR according to the model. For instance, descriptors indicating a favorable spatial orientation or optimal hydrogen bonding capacity can increase the predicted allosteric potential of a residue. In the waterfall plots, these positive contributions are visually represented, often pushing the prediction higher than the base value, signalling the model's recognition of features that are conducive to allosteric activity. Conversely, certain descriptors might exert a negative influence on the model's prediction, effectively reducing a residue's predicted allosteric potential. These could include metrics that suggest structural rigidity, lack of accessibility, or unfavorable energetic states that are less likely to support allosteric modulation. In waterfall plots, these negative contributions are depicted as downward steps, pulling the prediction below the base value and indicating characteristics that are possibly detrimental to allosteric functionality.

Collectively, these descriptors paint a detailed picture of the nano-environment of AFRs, emphasizing the intricate balance of spatial, structural, and energetic factors that govern allosteric potential. By elucidating these complex interactions, our models not only enhance our ability to predict AFRs with high precision but also contribute to a deeper understanding of the molecular mechanisms underlying allosteric regulation.
