# Supplementary material for: Protein allosteric site identification using machine learning and per amino acid residue reported internal protein nanoenvironment descriptors
Source: Comput Struct Biotechnol J. 2024 Oct 23;23:3907–19. doi: 10.1016/j.csbj.2024.10.036 (PMC11570862; doi:10.1016/j.csbj.2024.10.036)
Supplement: Supplementary file 3 — Supplementary material [file mmc3.pdf]

Descriptor Tables in the STING Database

Air
Electrostatic_Potential
Electrostatic_Potential_GN
Electrostatic_Potential_SW
Electrostatic_Potential_VD
Electrostatic_Potential_WNA
Energy_Density
Energy_Density_GN
Energy_Density_VD
Energy_Density_WNA
Entropy_Density
Entropy_Density_GN
Entropy_Density_SW
Entropy_Density_WNA
EPAtom
Evolutionary_Pressure
Atom_ASA
FPocket_Atom
atom_asa_dataframe
FPocket
Atom
Graph_Descriptor
Graph_Descriptor_SW
Graph_Descriptor_VD
Graph_Descriptor_WNA
HSSP
Ligand_Pocket_And_Water_Contact

Catalytic_Residue
chain_arranged
Mouth_Atom
Chain
Mouth
Multiple_Sequence_Alignment
Contact
Pocket_Atom
Prosite
Cross_Link_Order
Protein_Ligand_Contact
Cross_Link_Order_GN
Cross_Link_Order_SW
Residue_Contacts
Cross_Link_Order_VD
Residue_Contacts_GN
Cross_Link_Order_WNA
Residue_Contacts_WNA
Cross_Presence_Order
Cross_Presence_Order_DNA_GN
Rotamer
Cross_Presence_Order_DNA_SW
scaled_balanced_sponged
Cross_Presence_Order_DNA_VD
Cross_Presence_Order_DNA_WNA
Side_Chain_Orientation
Cross_Presence_Order_GN

Side_Chain_Orientation_GN
Cross_Presence_Order_SW
Side_Chain_Orientation_SW
Cross_Presence_Order_VD
Side_Chain_Orientation_VD
Cross_Presence_Order_WNA
Side_Chain_Orientation_WNA
Curvature_Atom
Curvature
Solvation
Curvature_GN
Solvation_GN
Curvature_SW
Solvation_SW
Curvature_VD
Solvation_VD
Curvature_WNA
Solvation_WNA
Curvature_Atom
Curvature
Solvation
Curvature_GN
Solvation_GN
Curvature_SW
Solvation_SW
Curvature_VD
Solvation_VD
Curvature_WNA

Solvation_WNA
Space_Clash
Density_Sponge
Density_Sponge_DNA
Sting_HSSP
Density_Sponge_DNA_GN
Stride
Density_Sponge_DNA_SW
Structure
Density_Sponge_DNA_VD
Density_Sponge_DNA_WNA
Unused_Contacts
Density_Sponge_GN
Unused_Contacts_GN
Density_Sponge_SW
Unused_Contacts_SW
Density_Sponge_VD
Unused_Contacts_VD
Density_Sponge_WNA
Unused_Contacts_WNA
Weighted_Contact_Number
Distances
Weighted_Contact_Number_GN
distribution.png
Weighted_Contact_Number_SW
DSSP
Weighted_Contact_Number_VD
EFR_slides
Weighted_Contact_Number_WNA

All tables from the STING database were selectively retrieved to match the PDB_ID codes from the Allosteric database.

Descriptor Tables in the STING Database

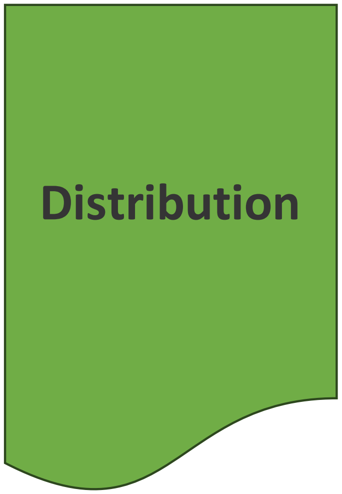

Air

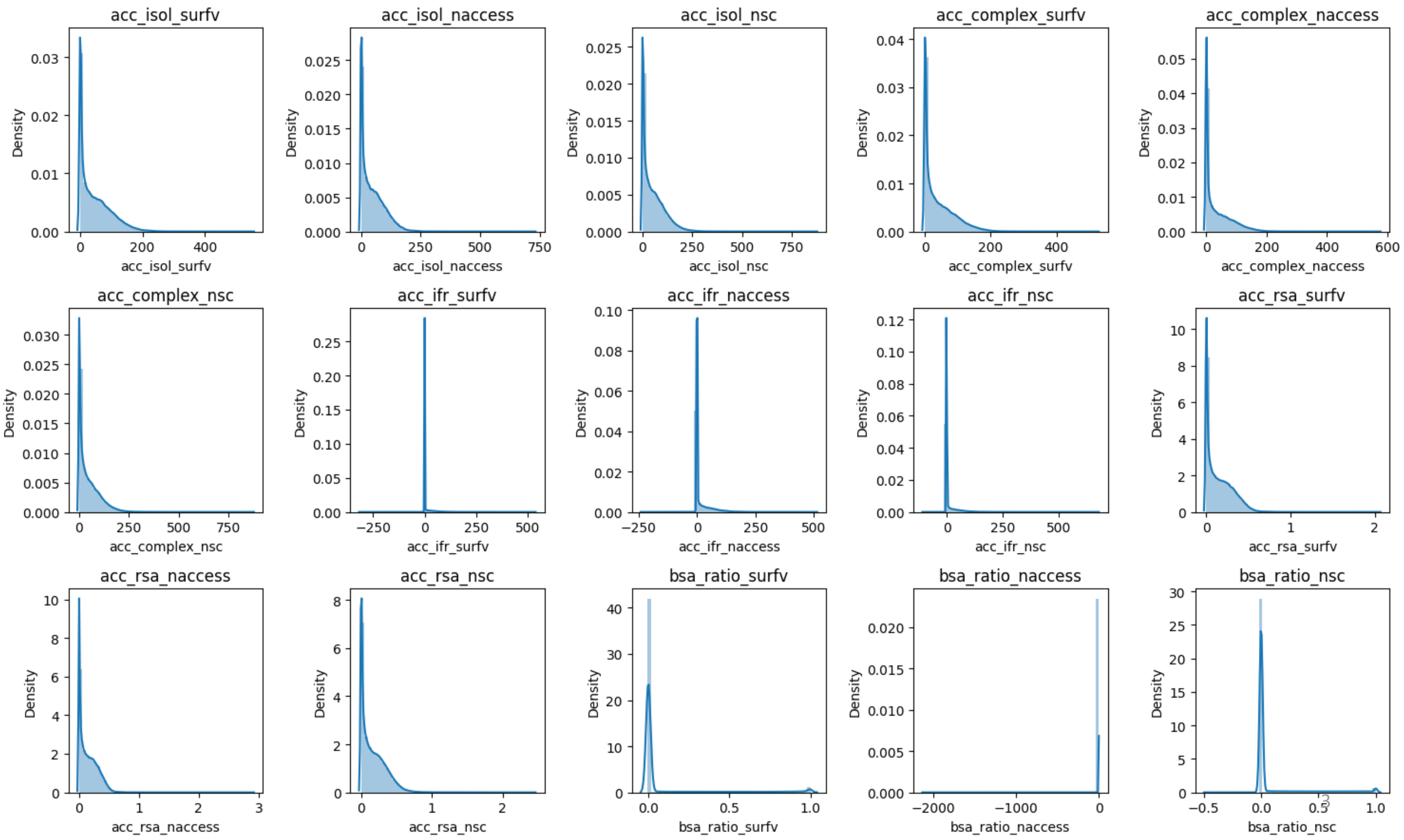

acc_isol_surfv
acc_isol_naccess
acc_isol_nsc
acc_complex_surfv
acc_complex_naccess
acc_complex_nsc
acc_ifr_surfv
acc_ifr_naccess
acc_ifr_nsc
acc_rsa_surfv
acc_rsa_naccess
acc_rsa_nsc
bsa_ratio_surfv
bsa_ratio_naccess
bsa_ratio_nsc
Density

Electrostatic_Potential

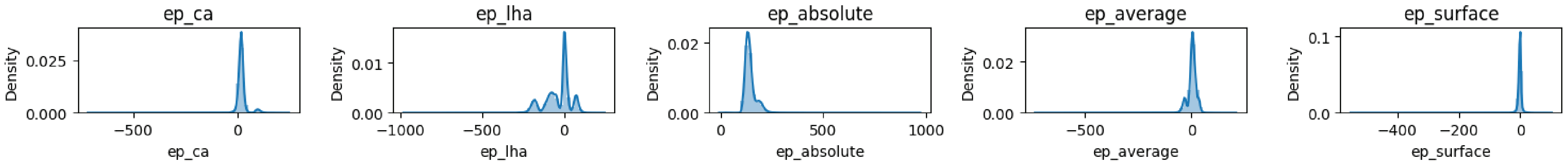

Electrostatic_Potential_GN

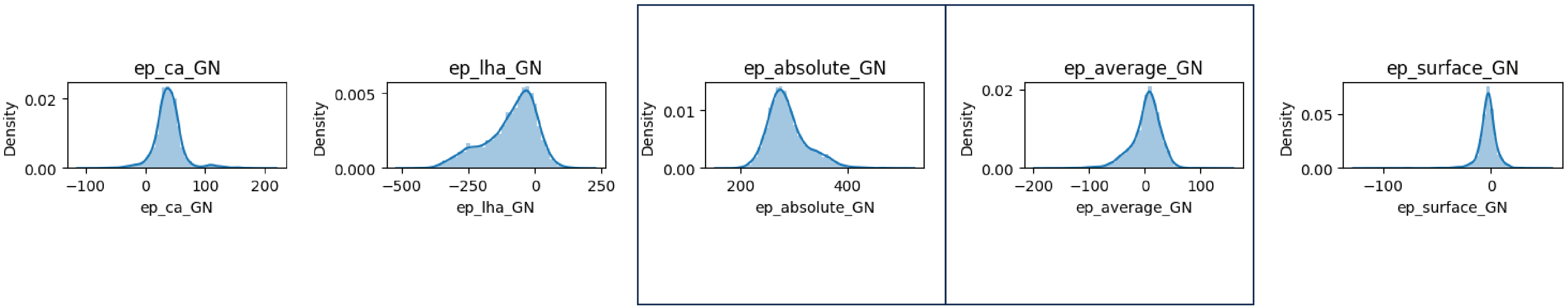

Electrostatic_Potential_SW

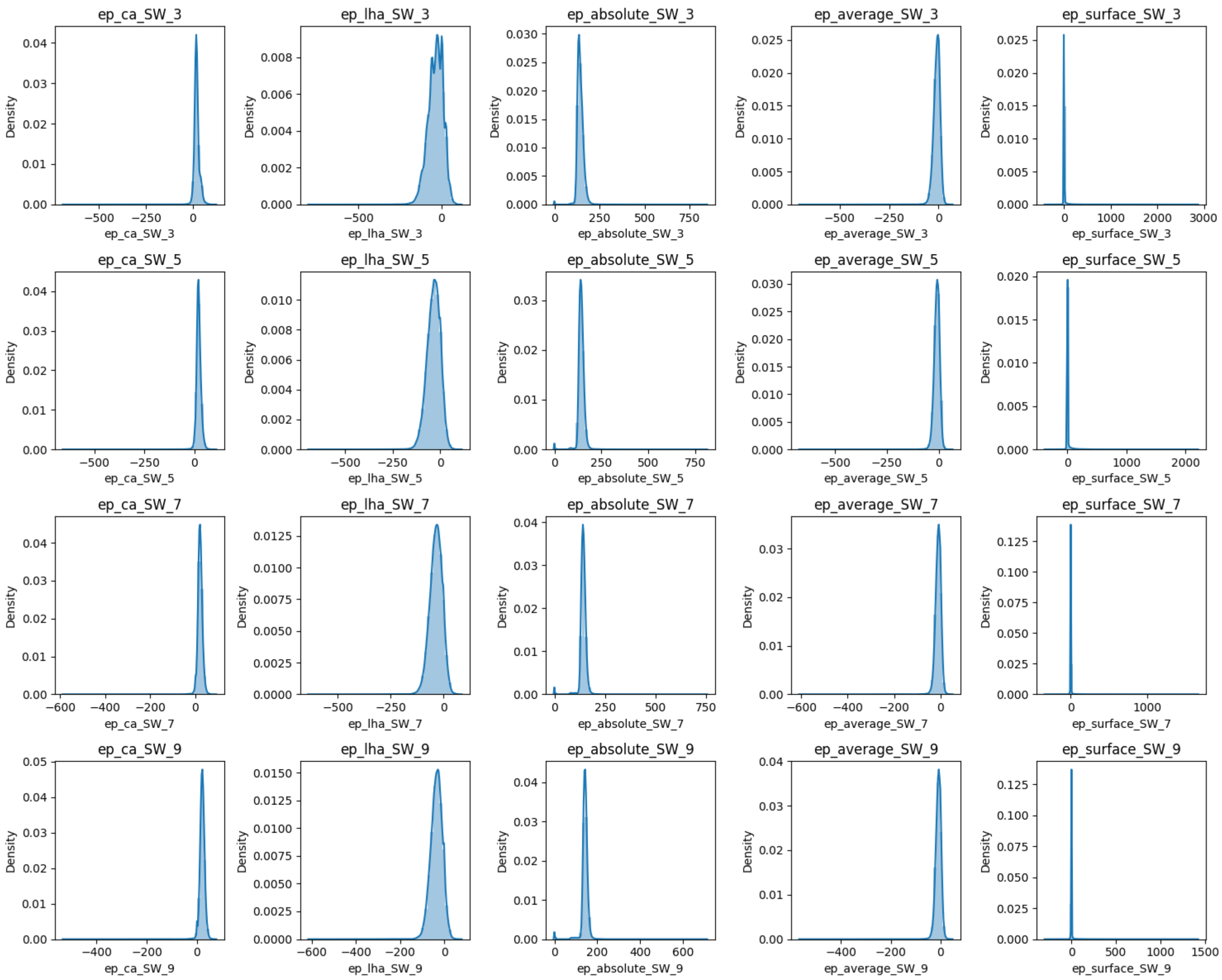

Electrostatic_Potential_VD

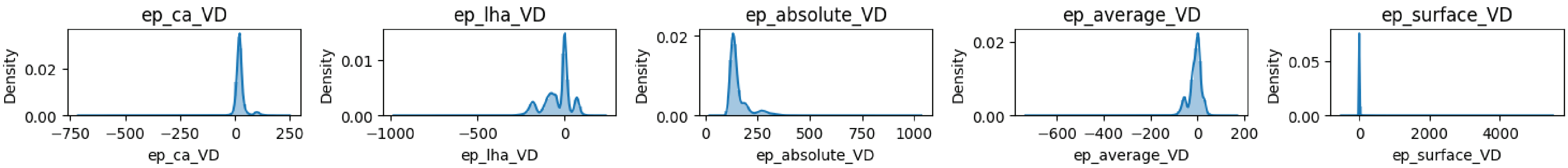

Electrostatic_Potential_WNA

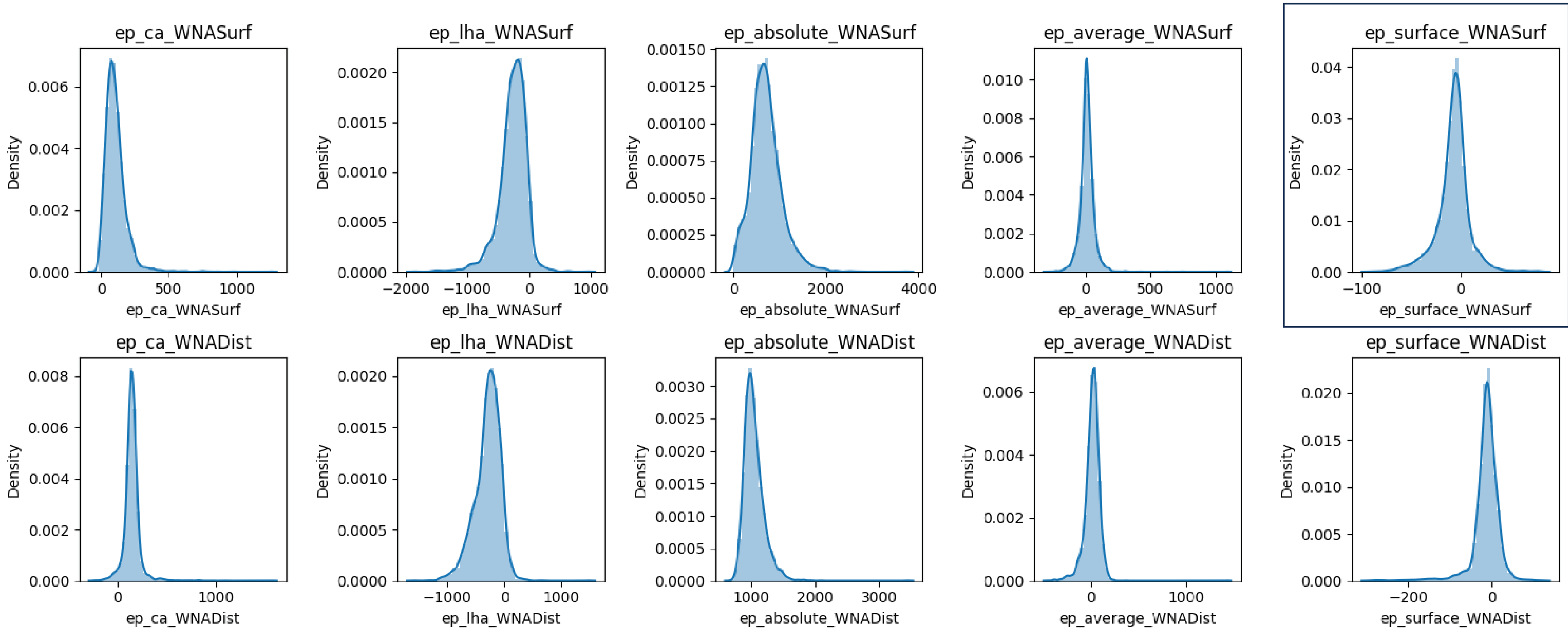

Energy_Density

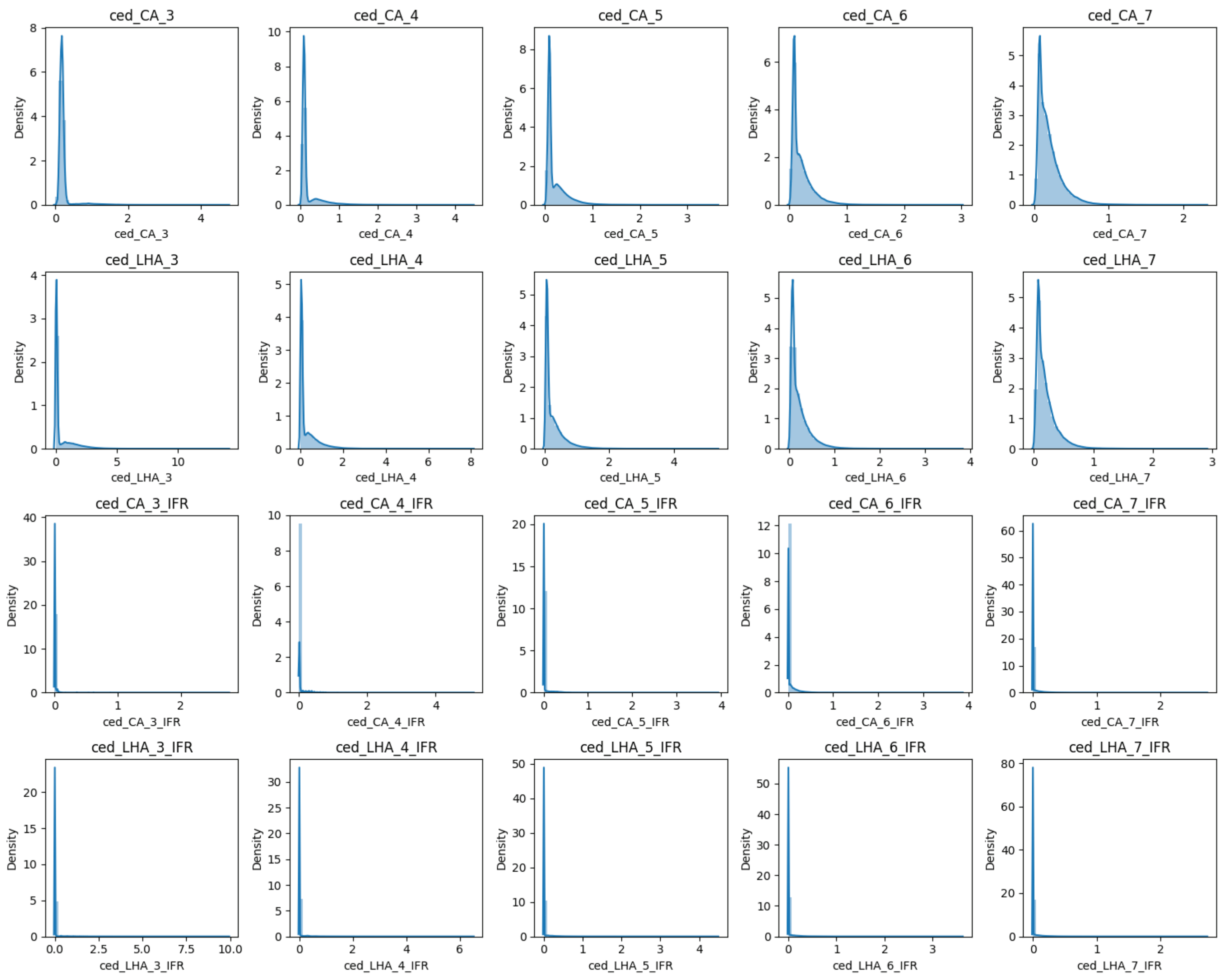

Energy_Density_GN

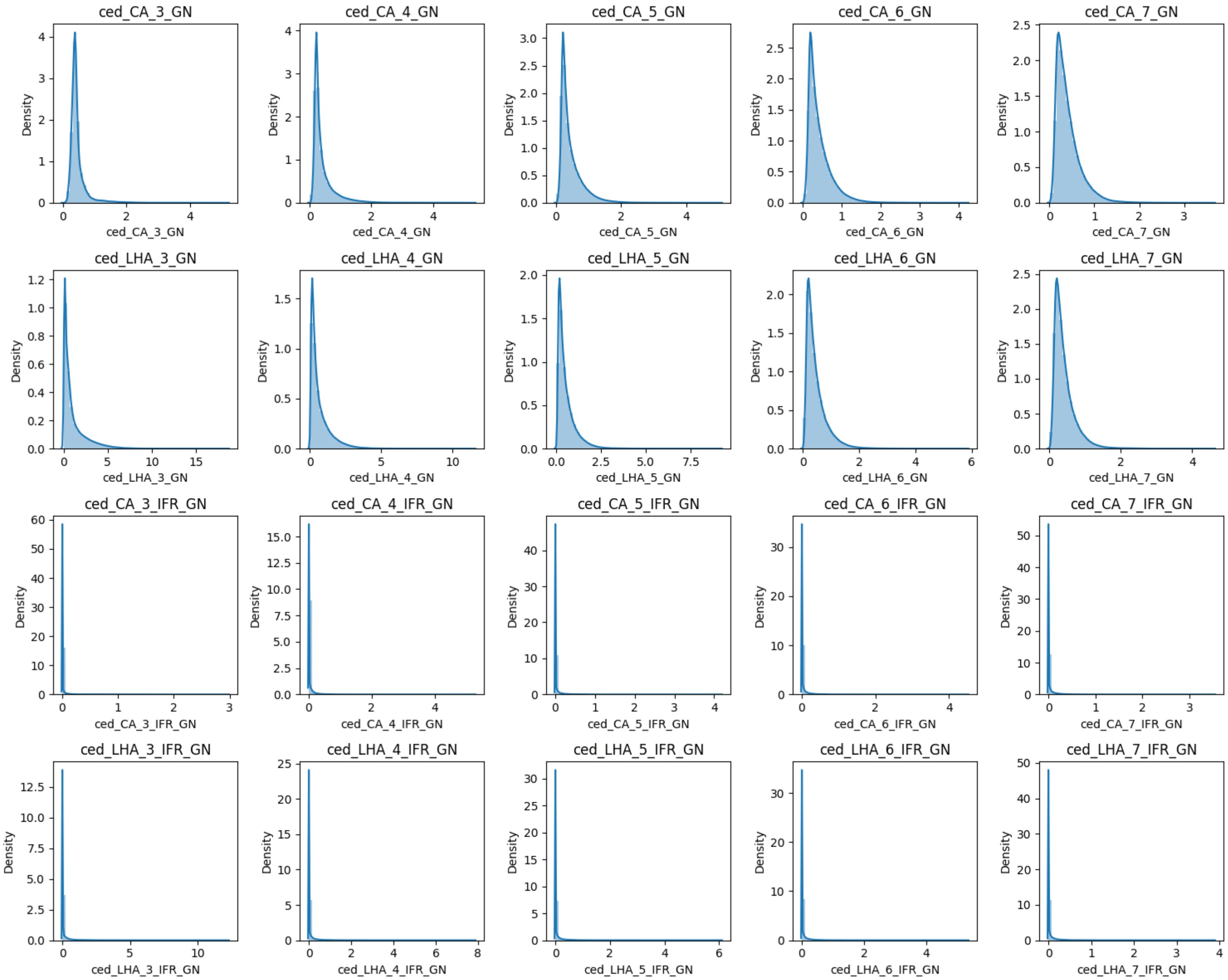

Energy_Density_VD

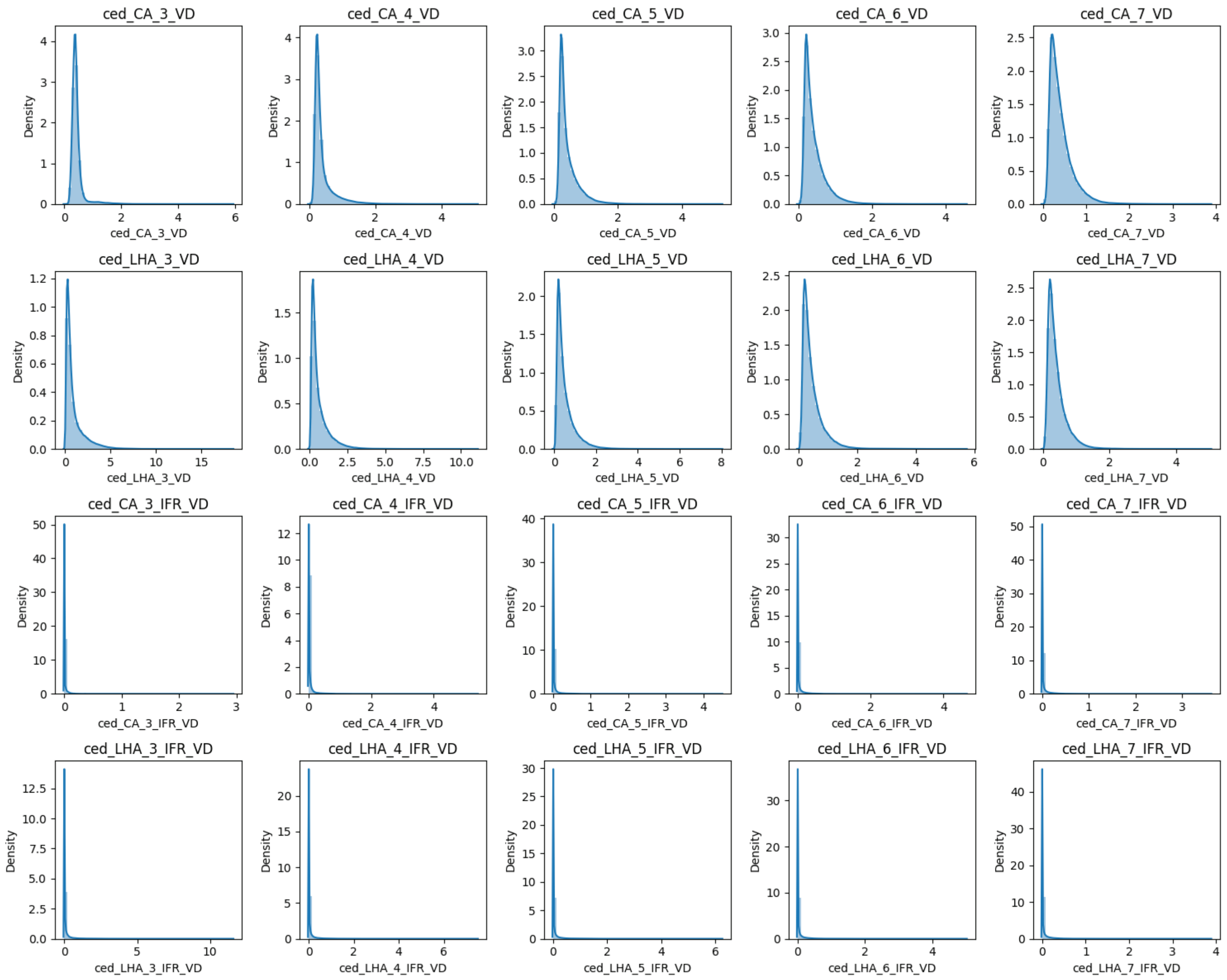

Energy_Density_WNA

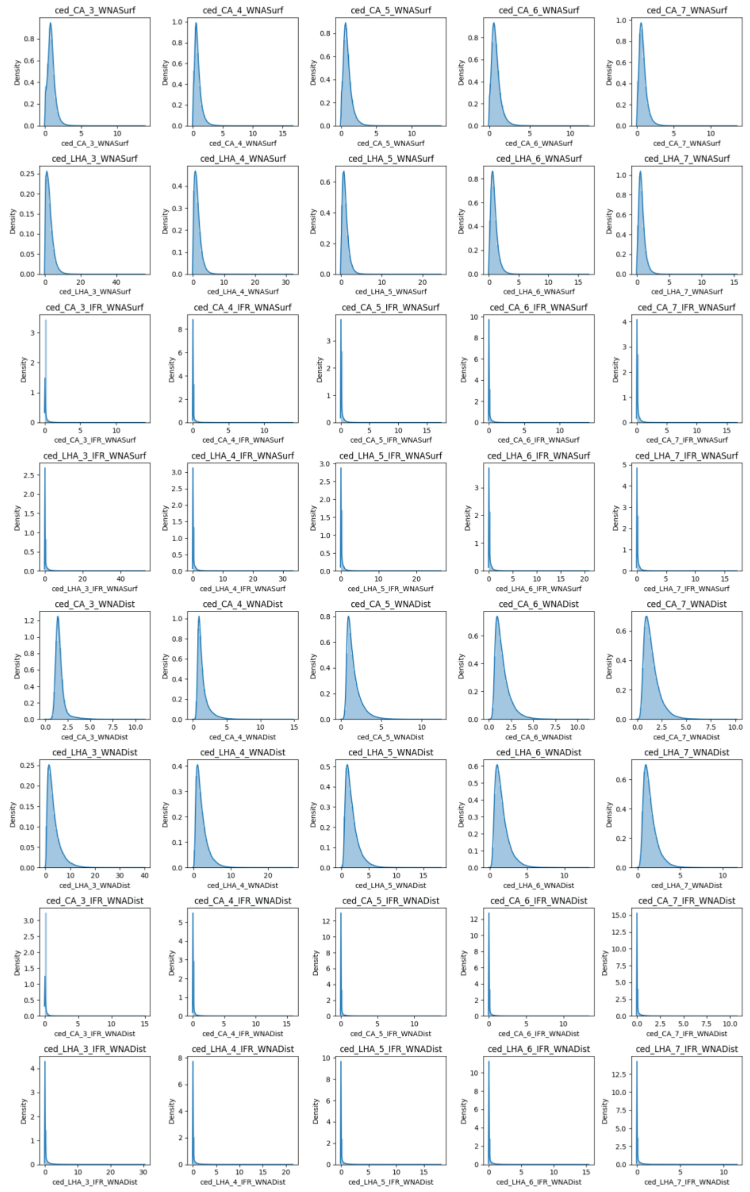

Entropy_Density

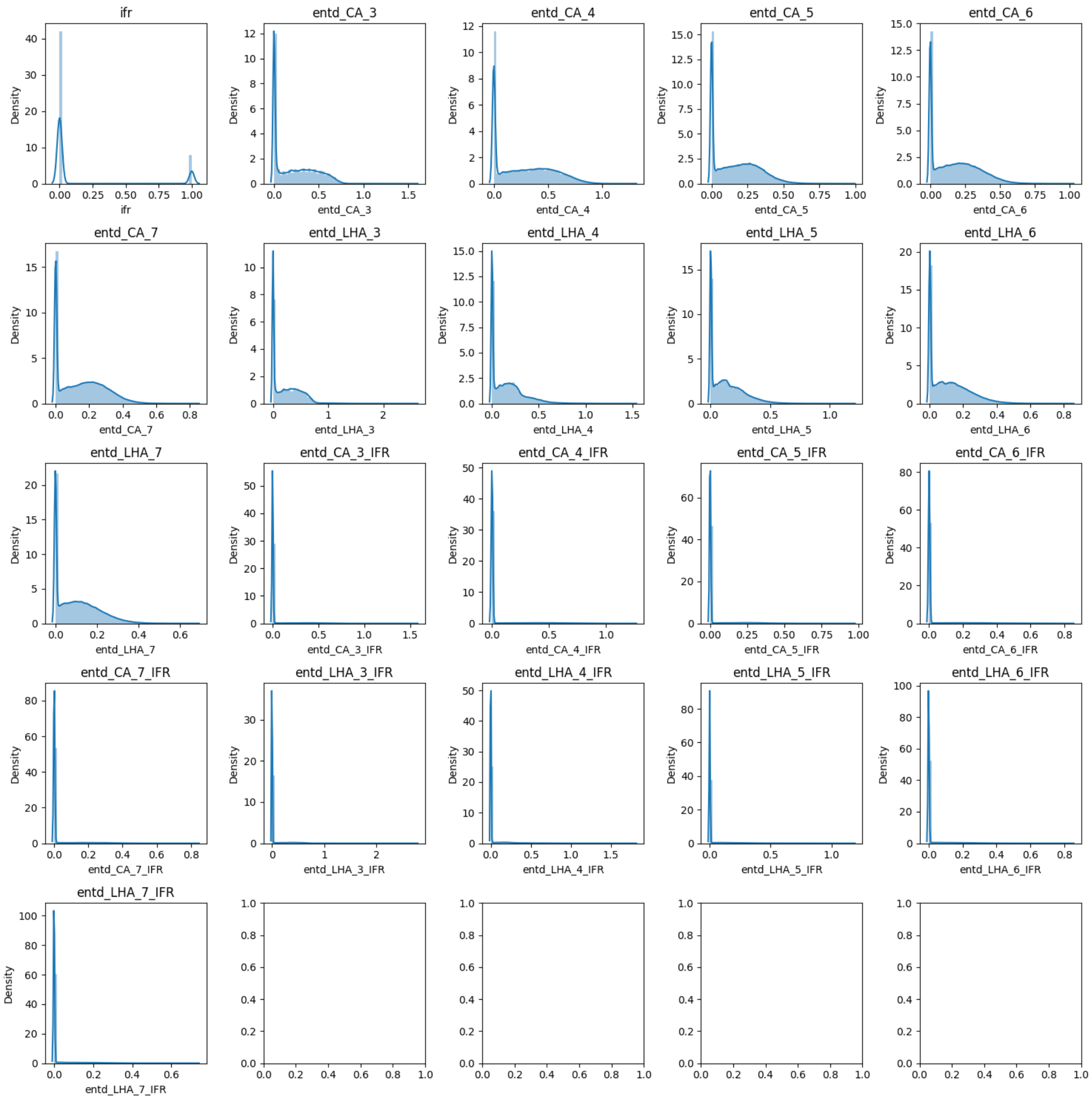

Entropy_Density_GN

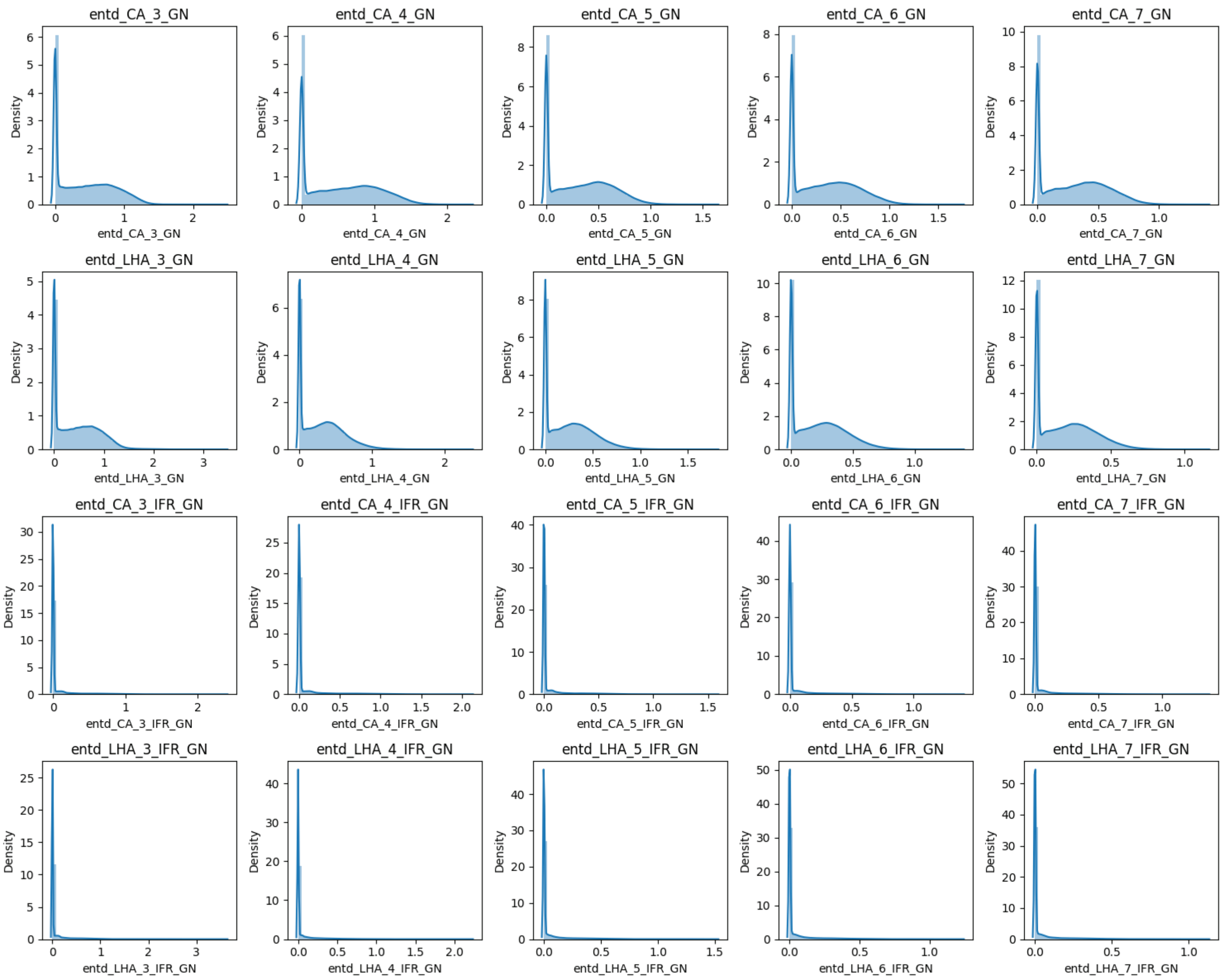

Entropy_Density_SW

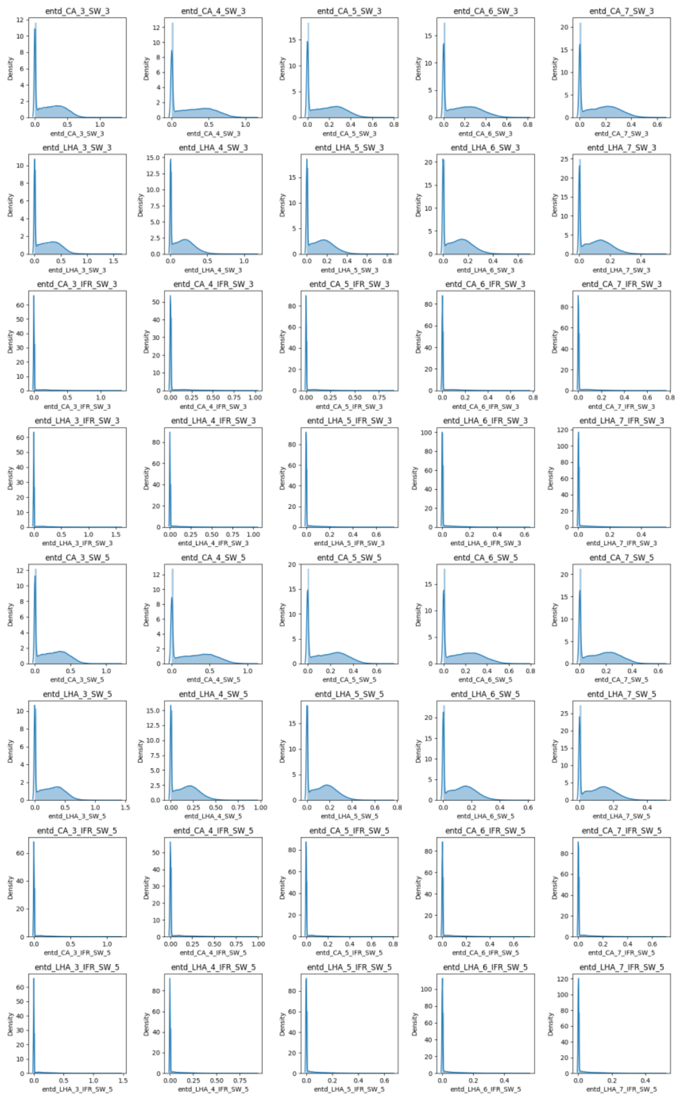

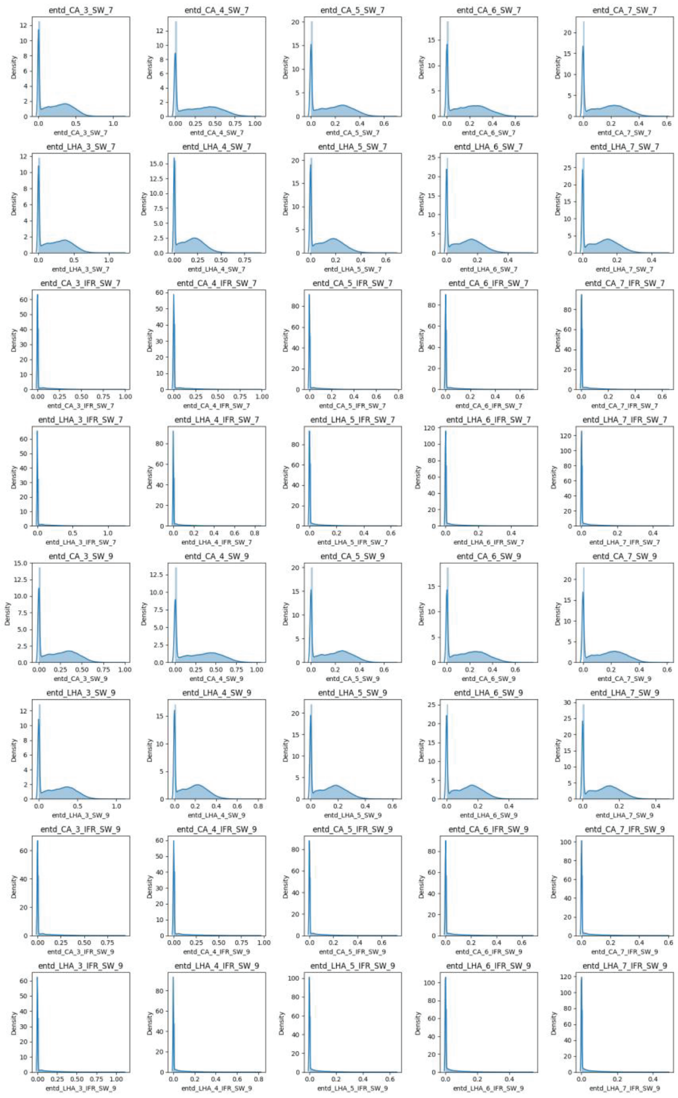

Entropy_Density_WNA

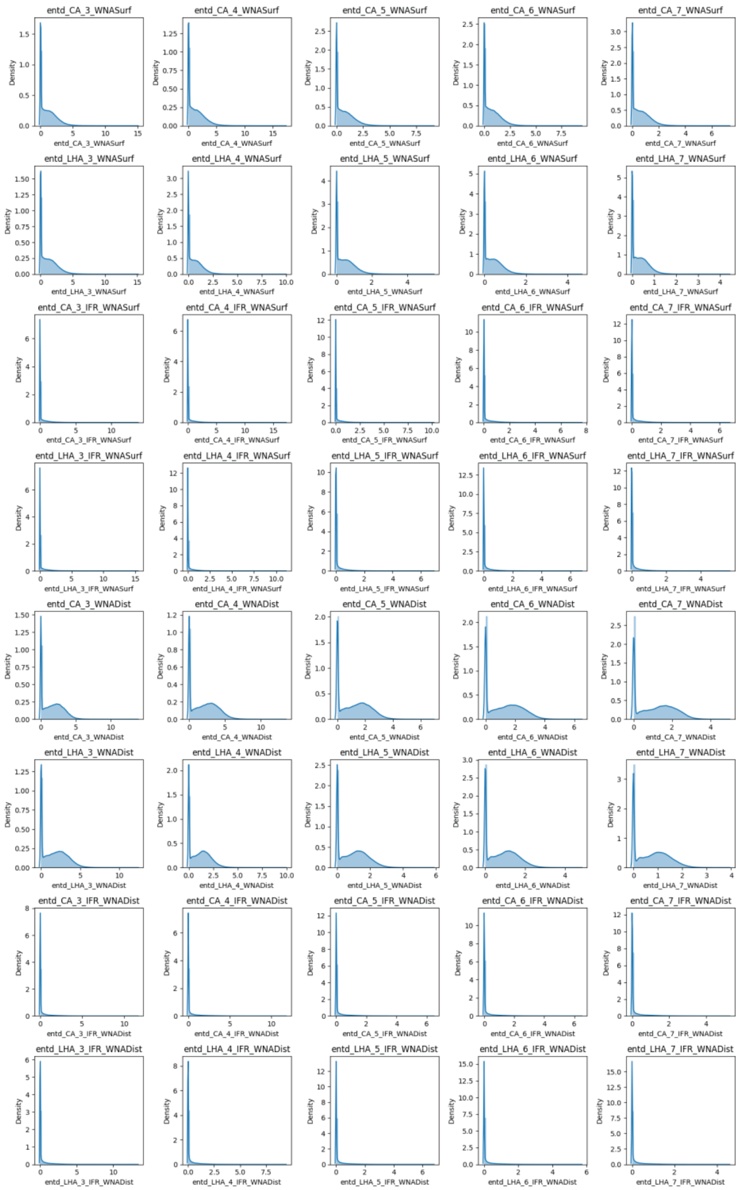

FPocket

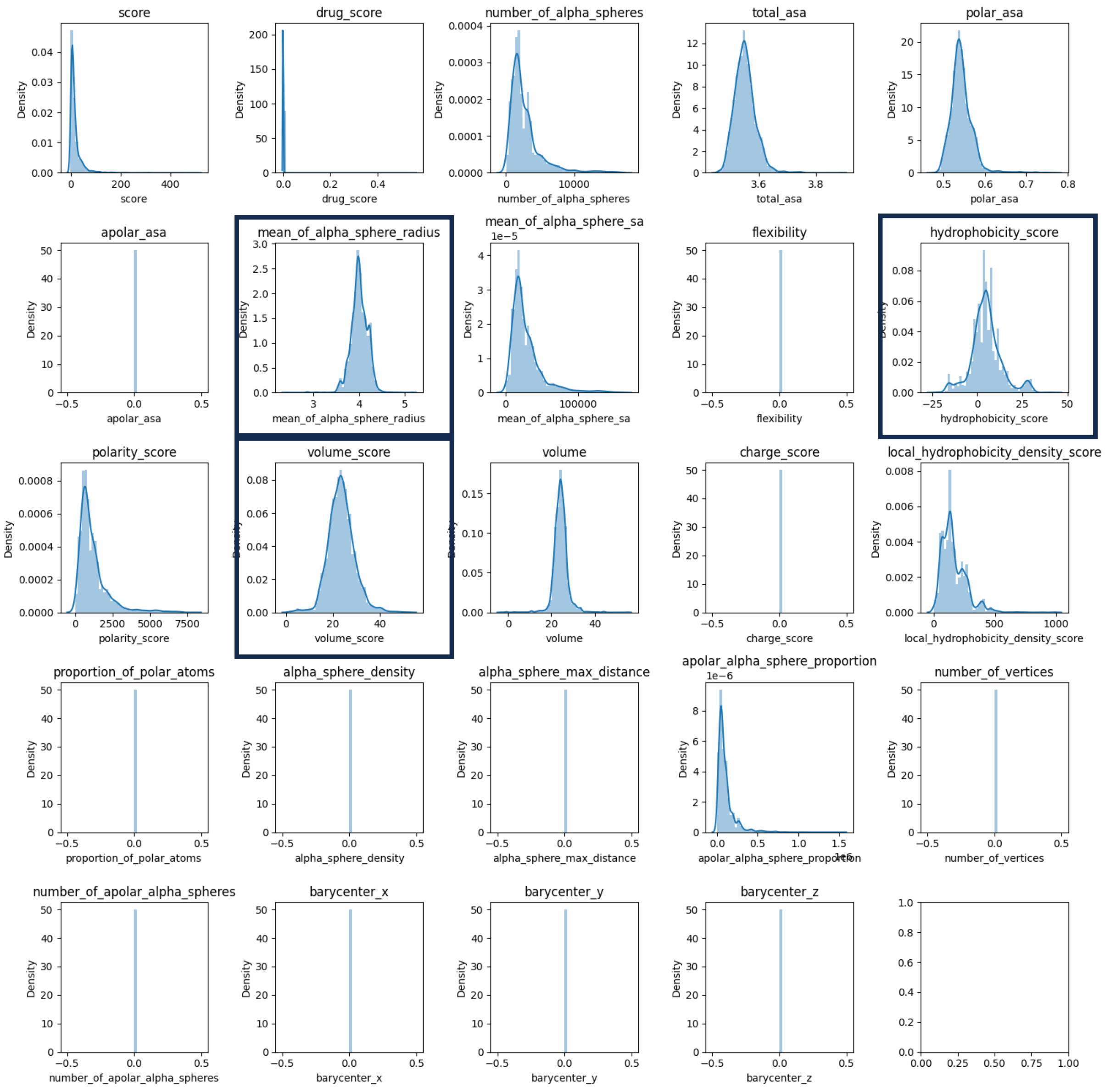
score
drug_score
number_of_alpha_spheres
total_asa
polar_asa
apolar_asa
mean_of_alpha_sphere_radius
mean_of_alpha_sphere_sa
flexibility
hydrophobicity_score
polarity_score
volume_score
volume
charge_score
local_hydrophobicity_density_score
proportion_of_polar_atoms
alpha_sphere_density
alpha_sphere_max_distance
apolar_alpha_sphere_proportion
number_of_vertices
number_of_apolar_alpha_spheres
barycenter_x
barycenter_y
barycenter_z
Density

Graph_Descriptor

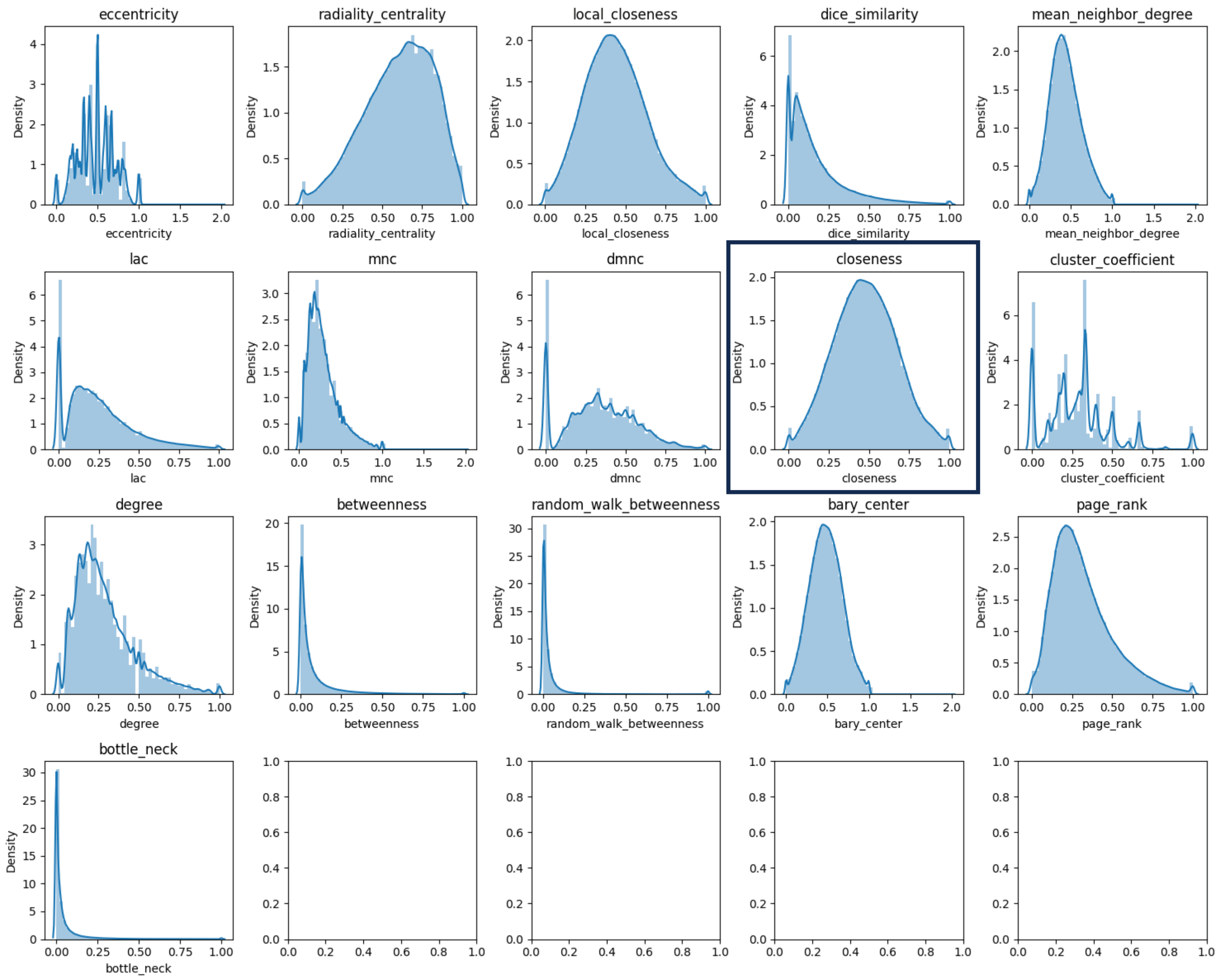

Graph_Descriptor_SW

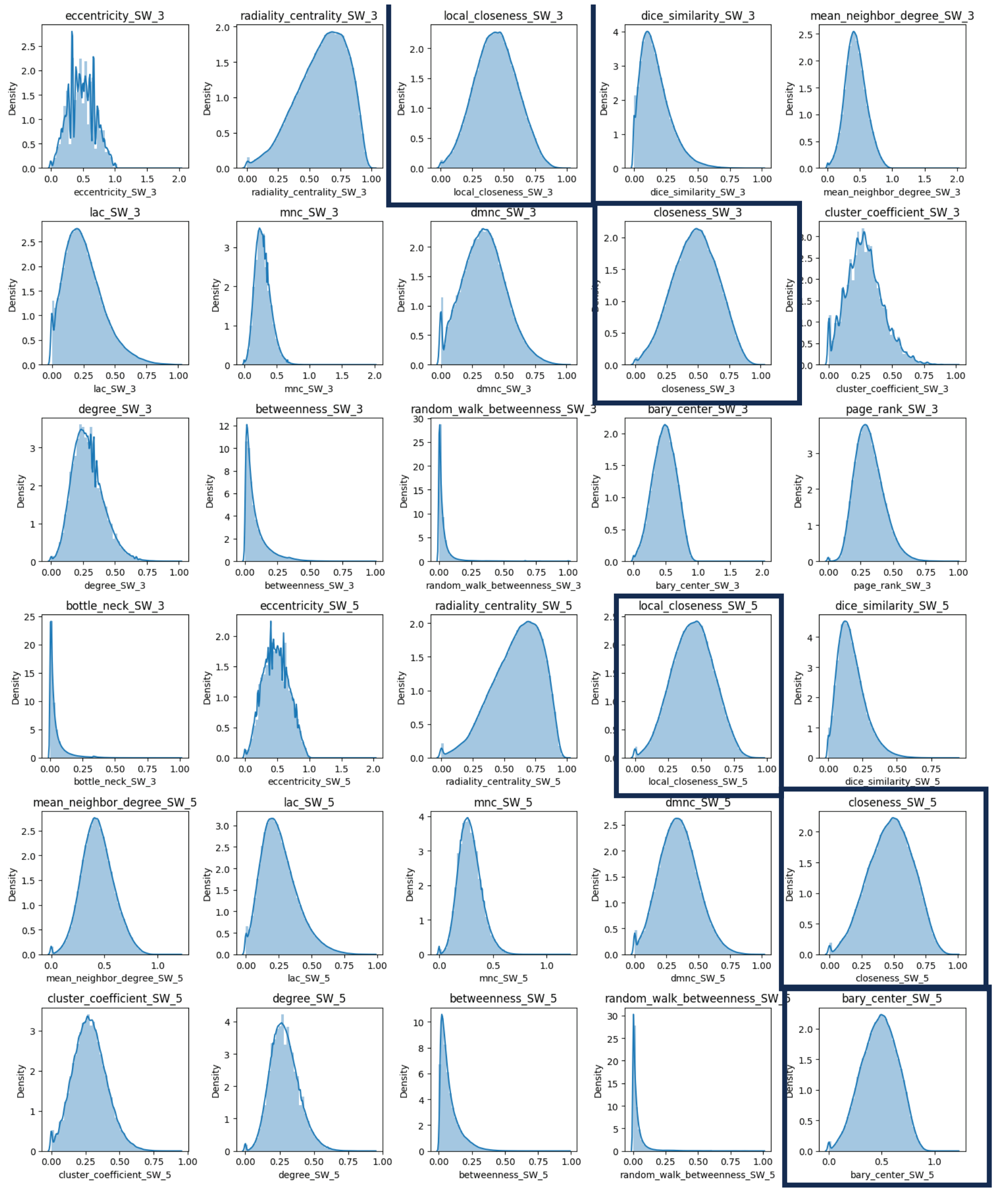

Graph_Descriptor_SW

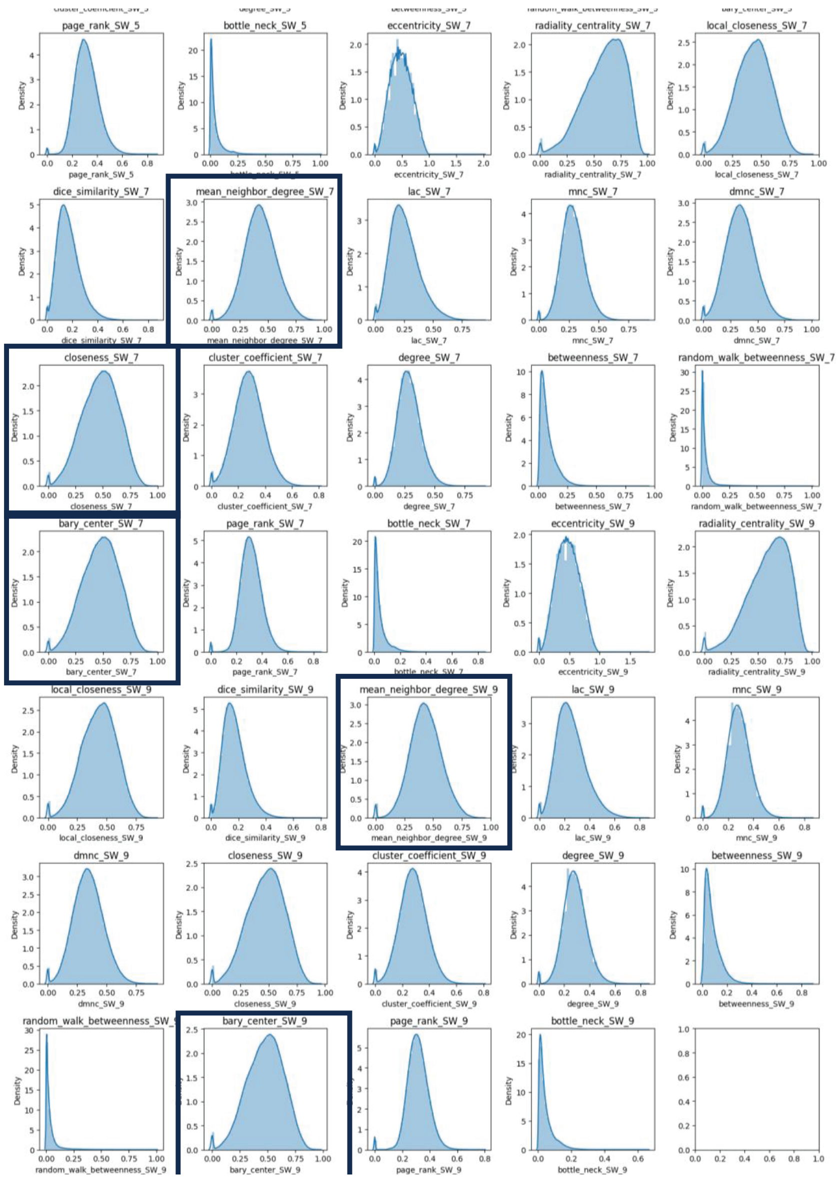

Graph_Descriptor_VD

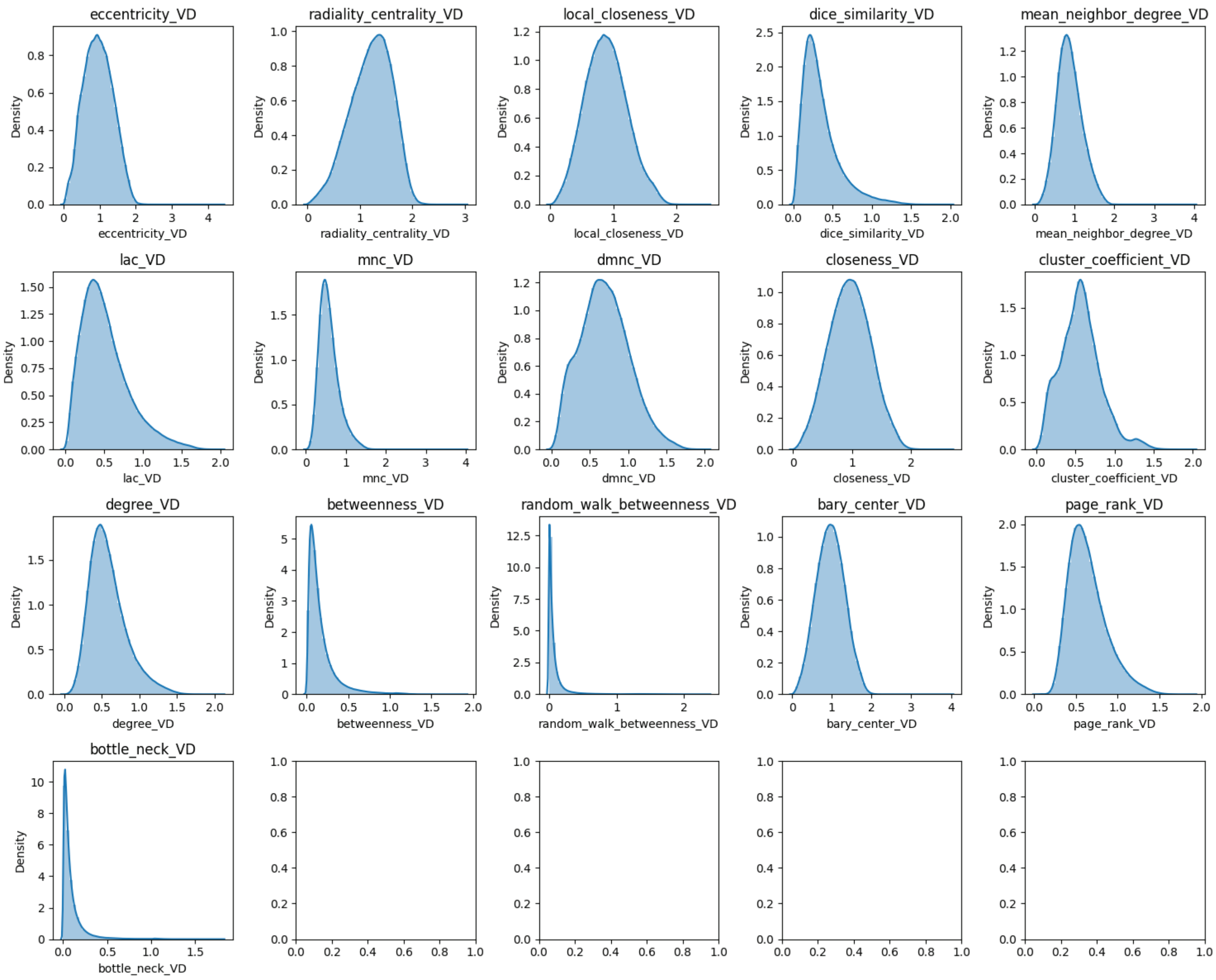

Graph_Descriptor_WNA

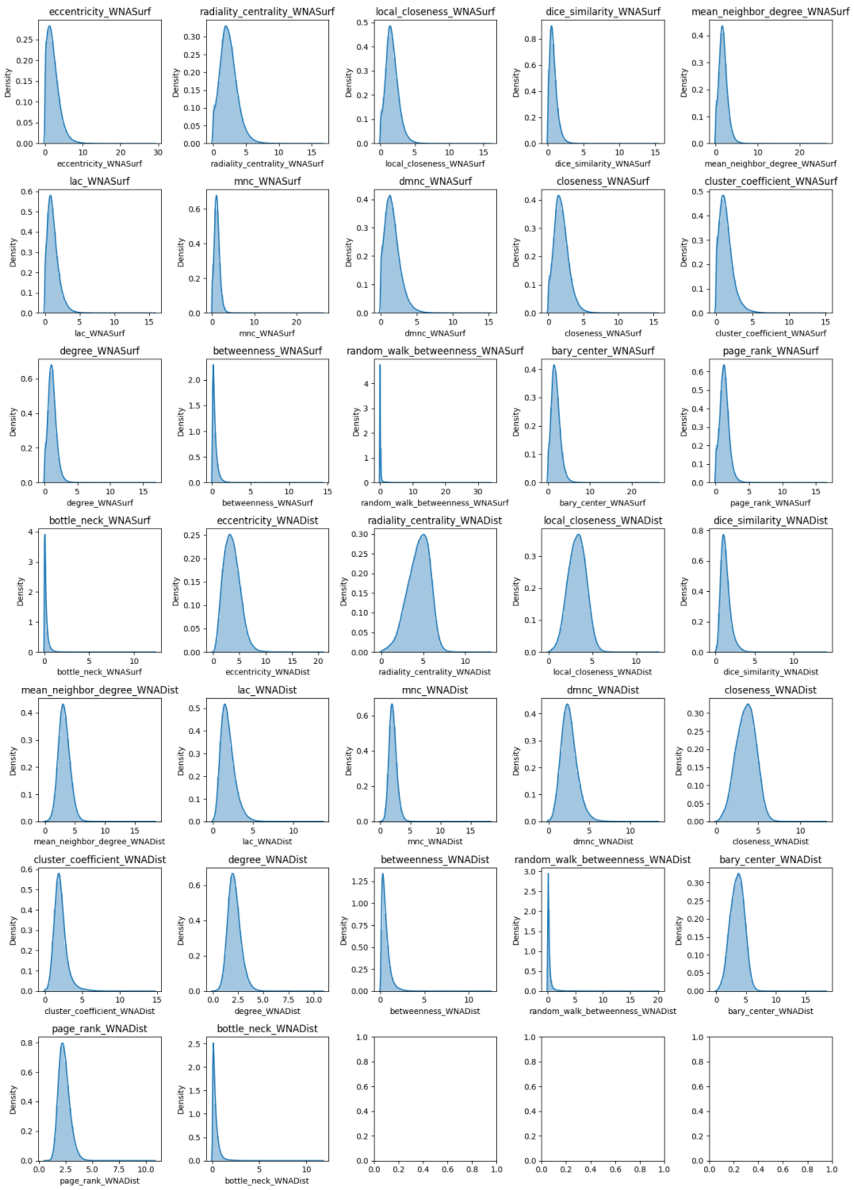

HSSP

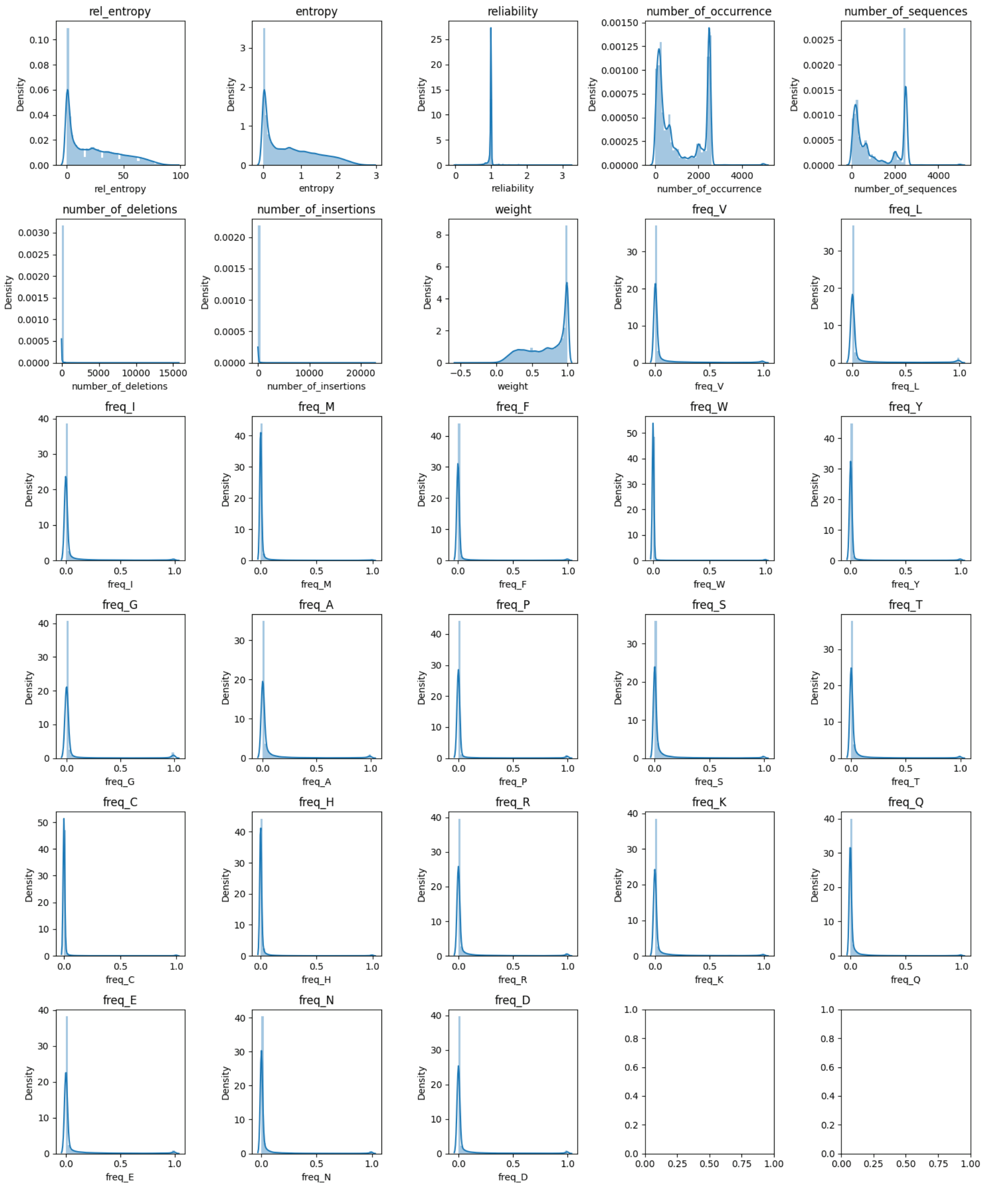

rel_entropy
entropy
reliability
number_of_occurrence
number_of_sequences
number_of_deletions
number_of_insertions
weight
freq_V
freq_L
freq_I
freq_M
freq_F
freq_W
freq_Y
freq_G
freq_A
freq_P
freq_S
freq_T
freq_C
freq_H
freq_R
freq_K
freq_Q
freq_E
freq_N
freq_D
Density

Cross_Link_Order_GN

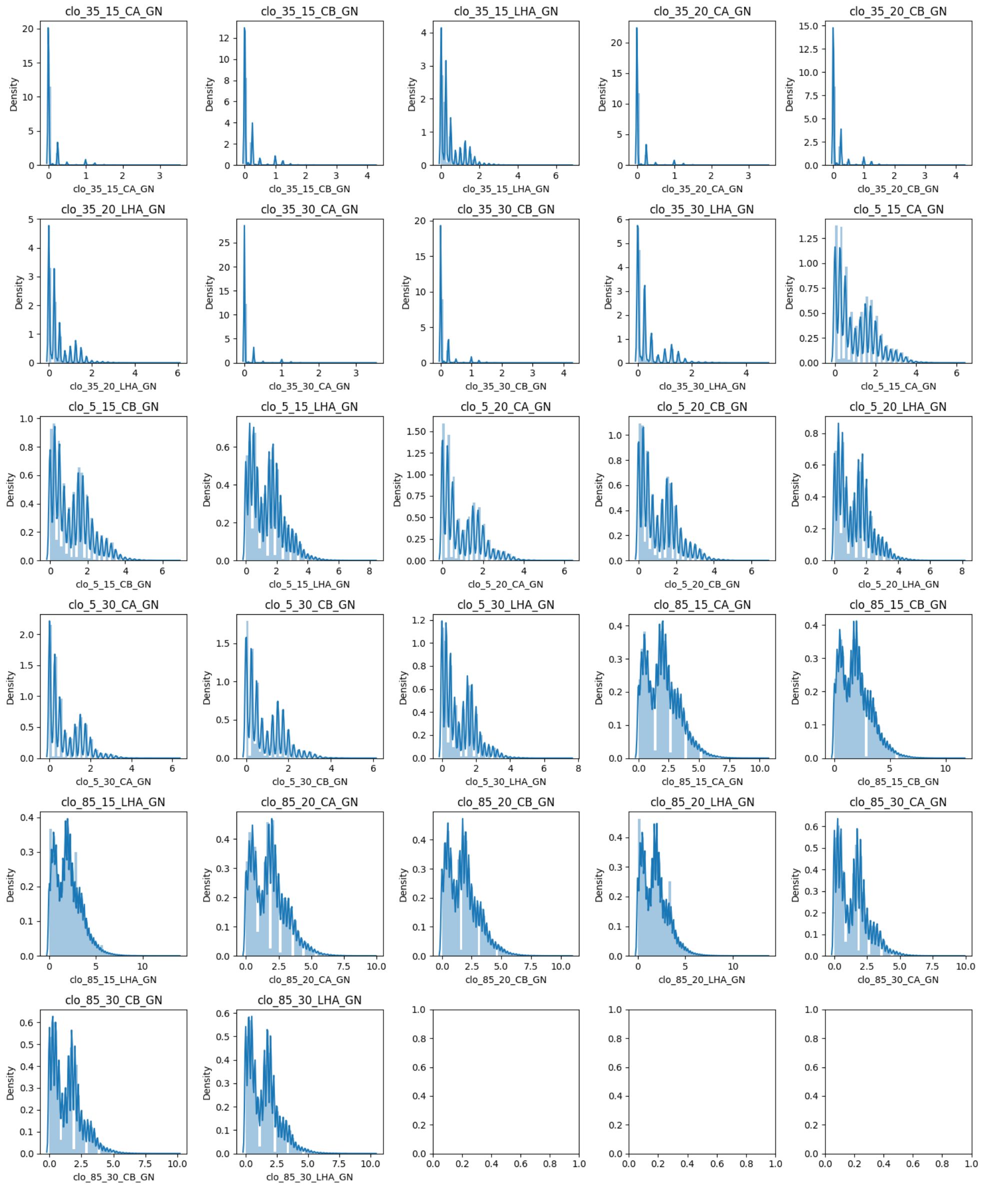

Cross_Link_Order_SW

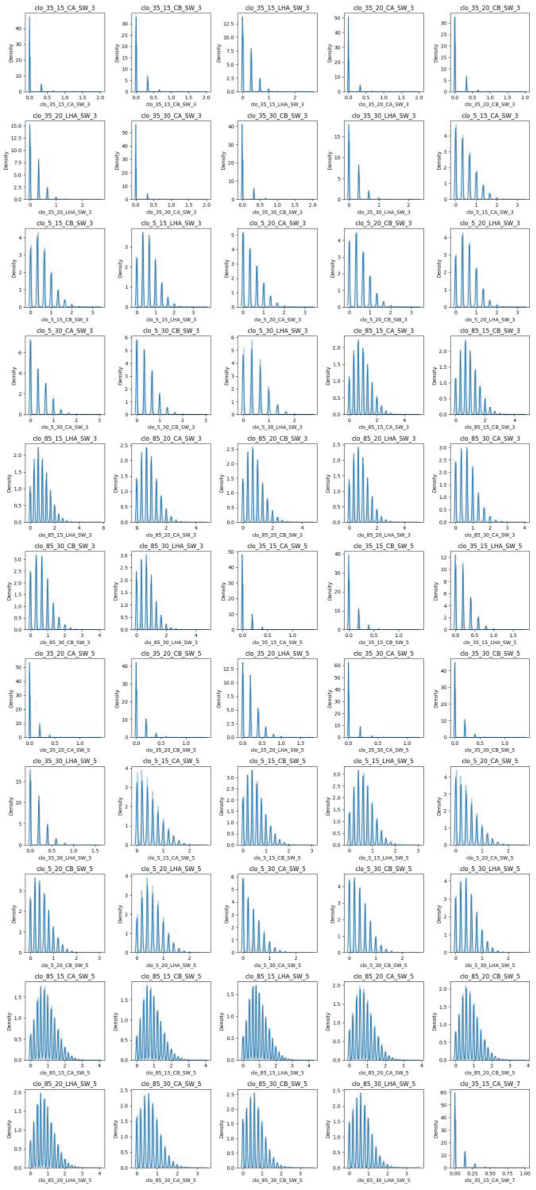

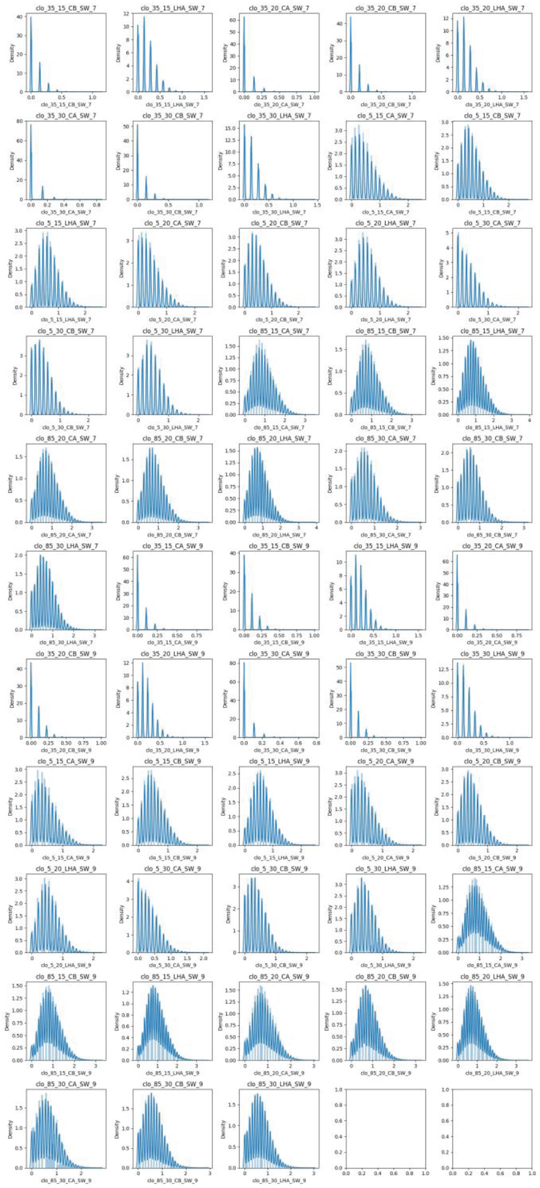

Cross_Link_Order_VD

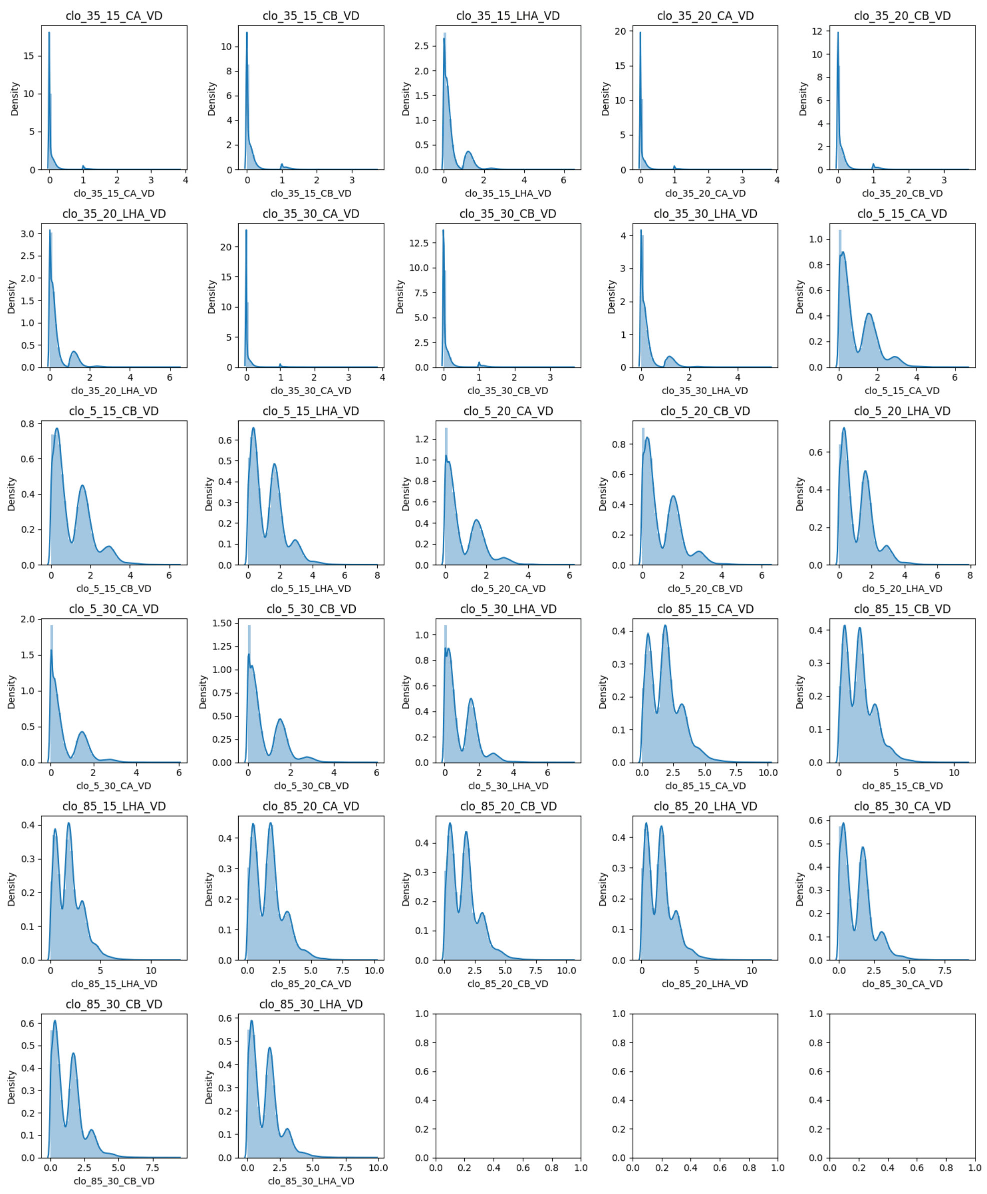

Residue_Contacts_GN

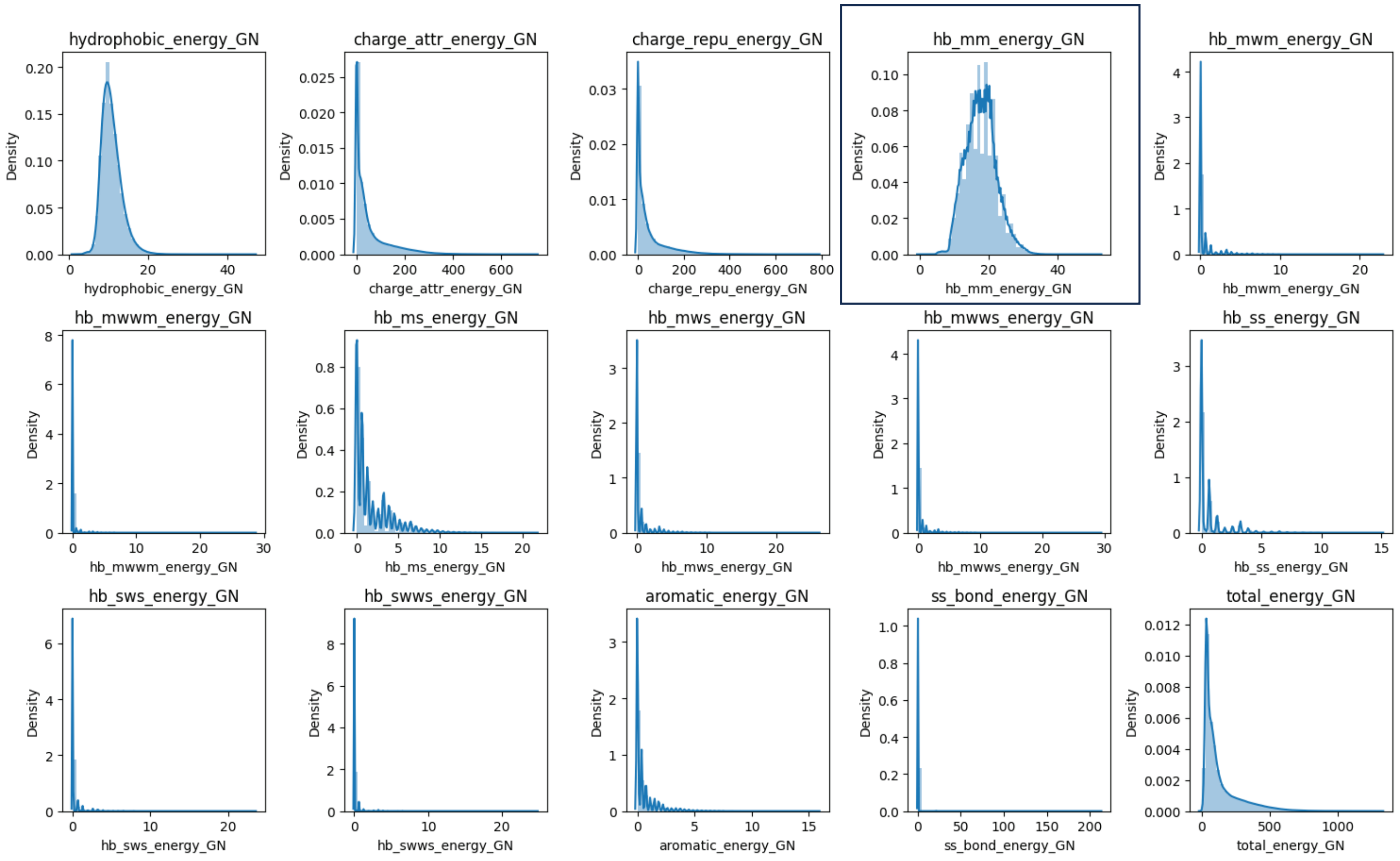

Cross_Link_Order_WNA

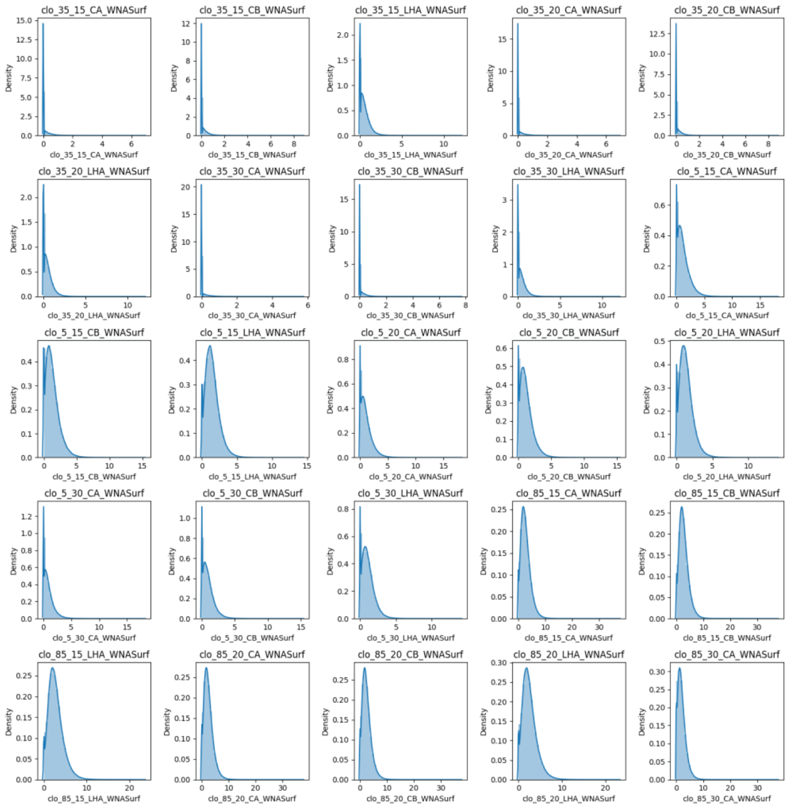

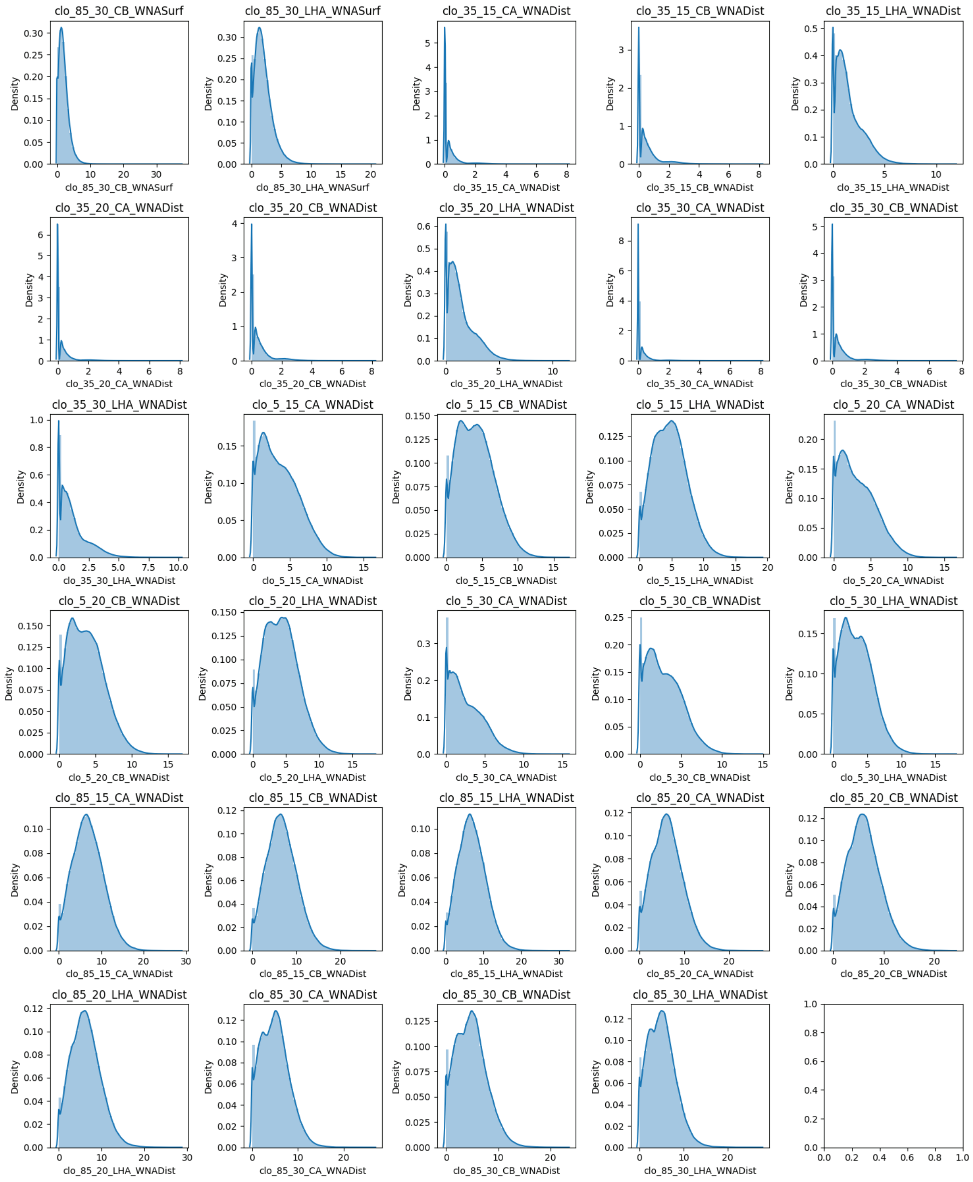

Residue_Contacts_WNA

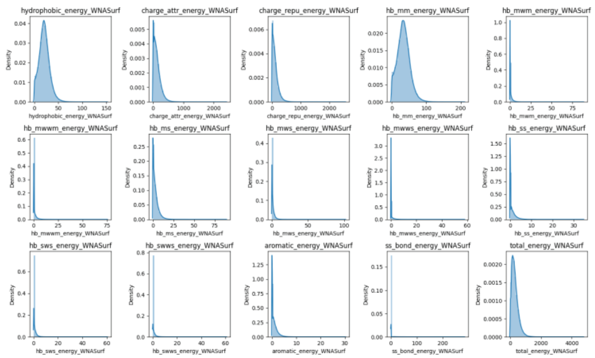

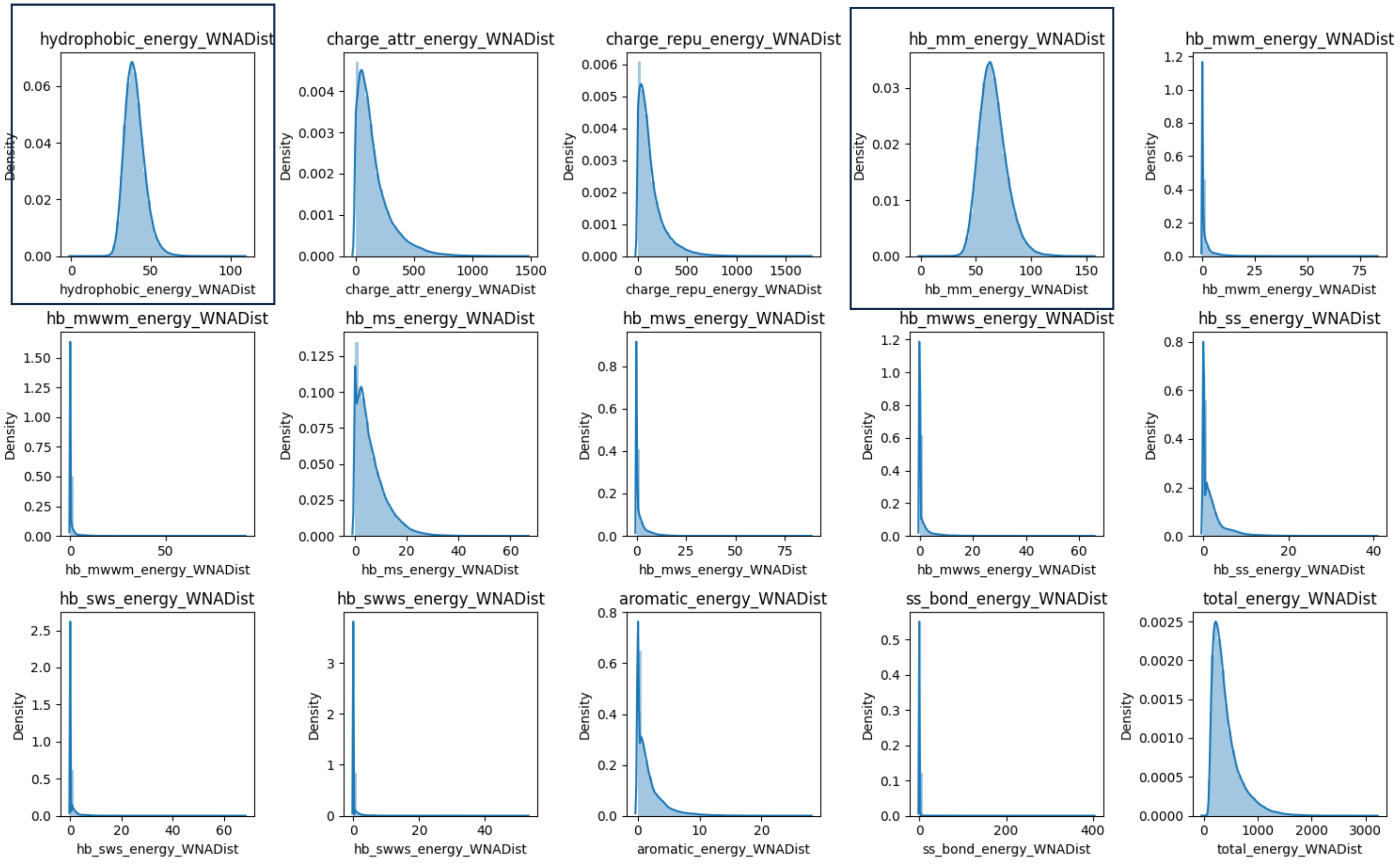

Side_Chain_Orientation

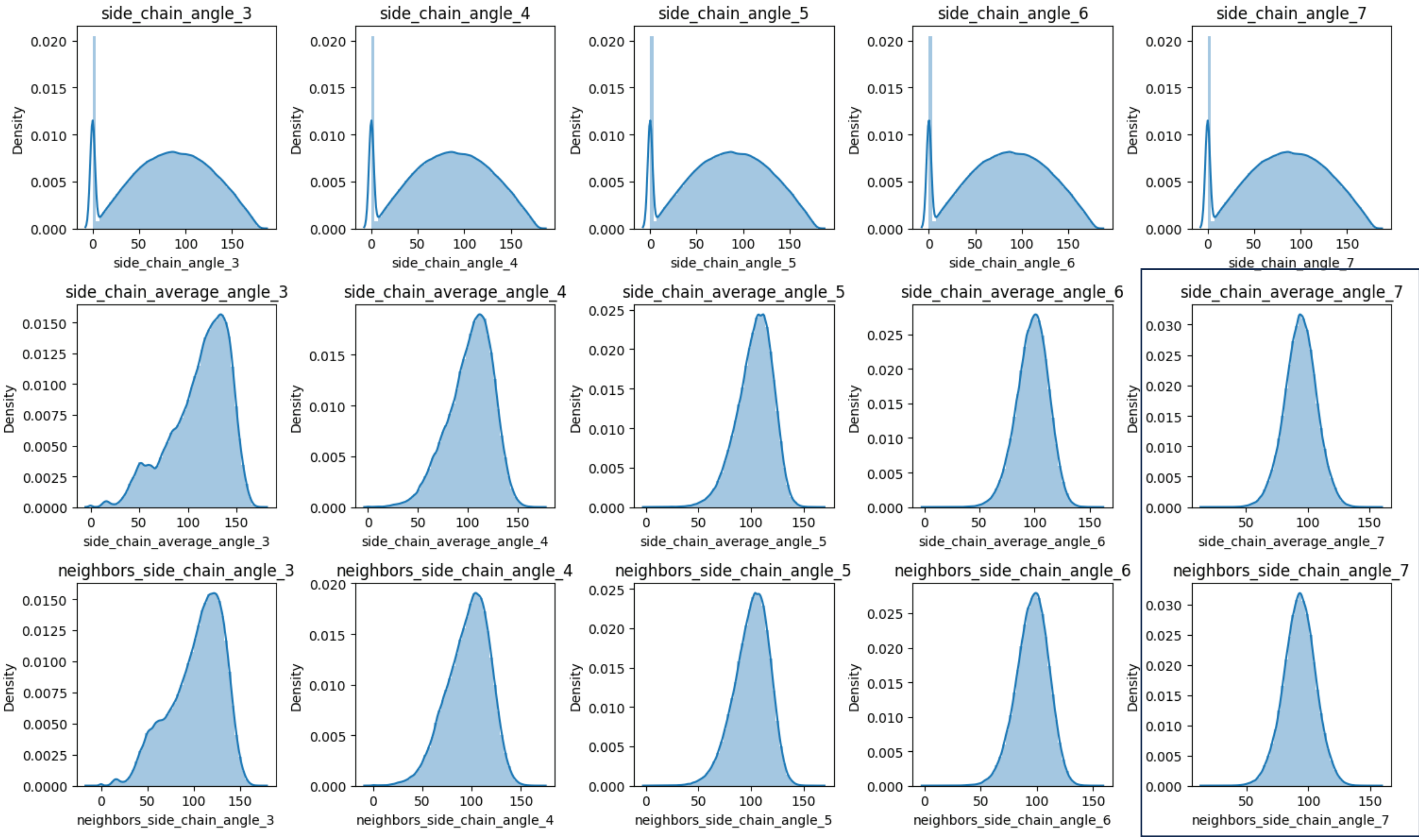

Cross_Presence_Order_GN

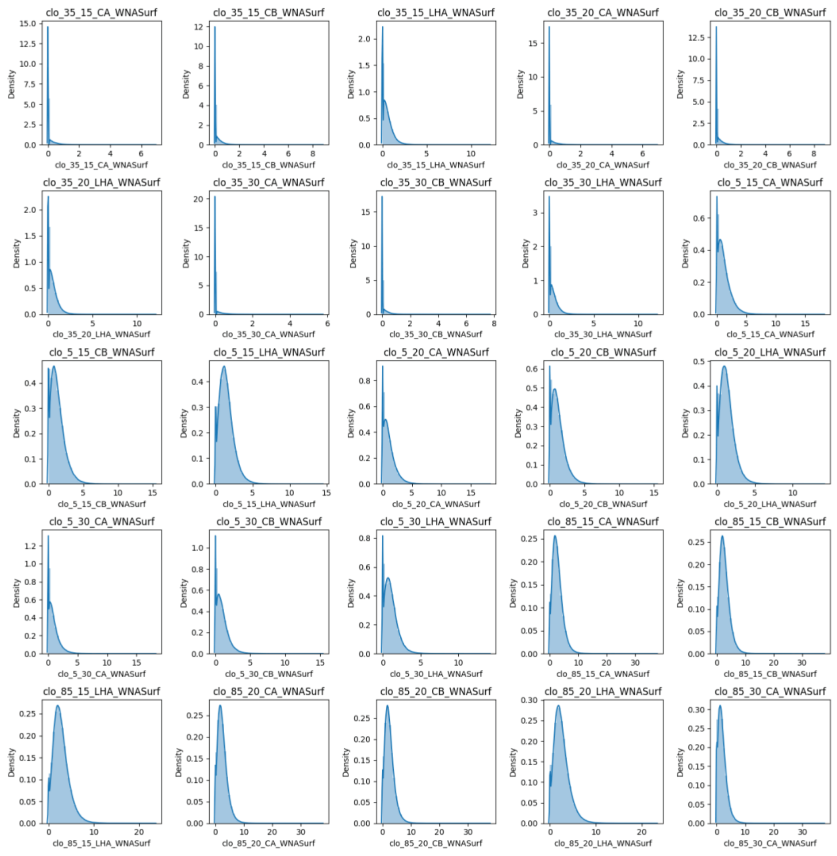

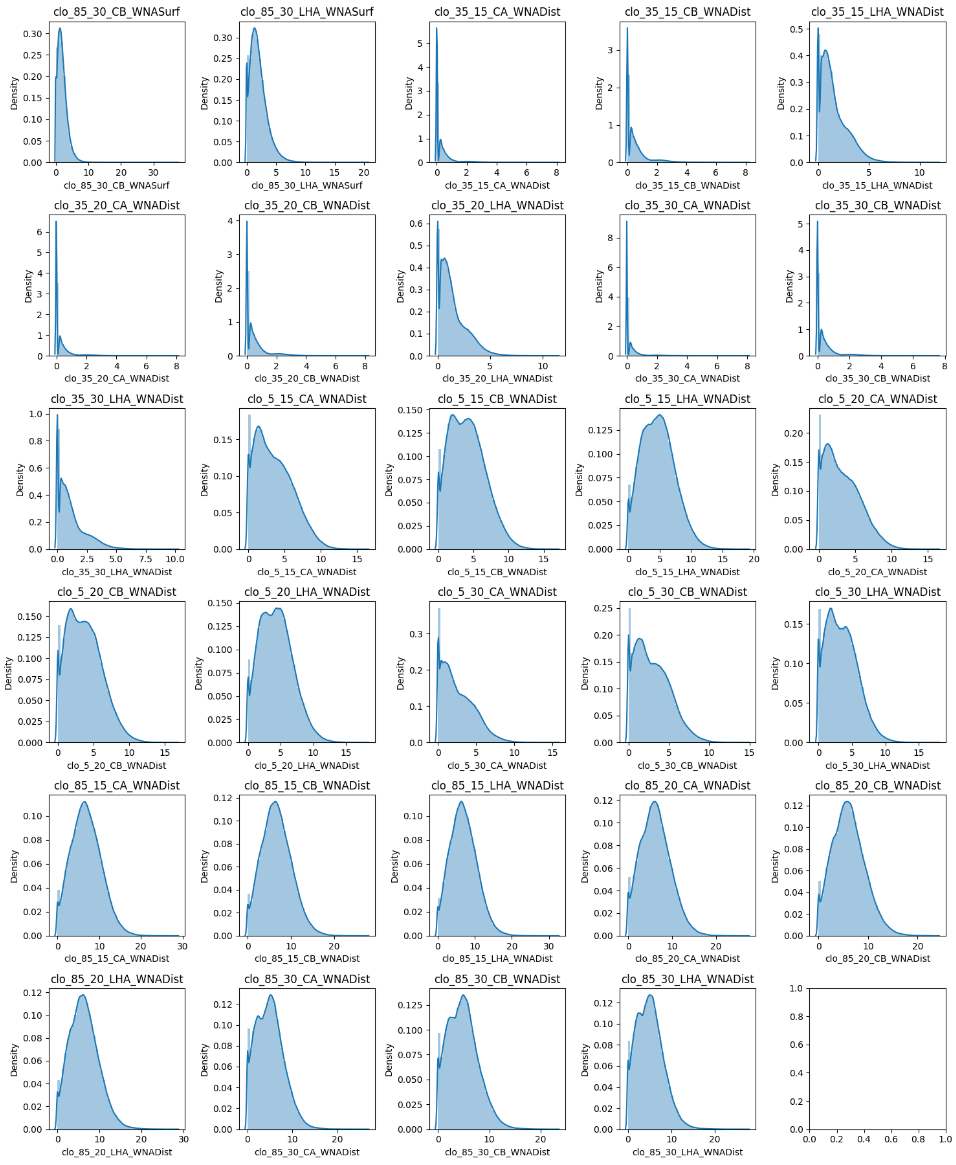

Side_Chain_Orientation_GN

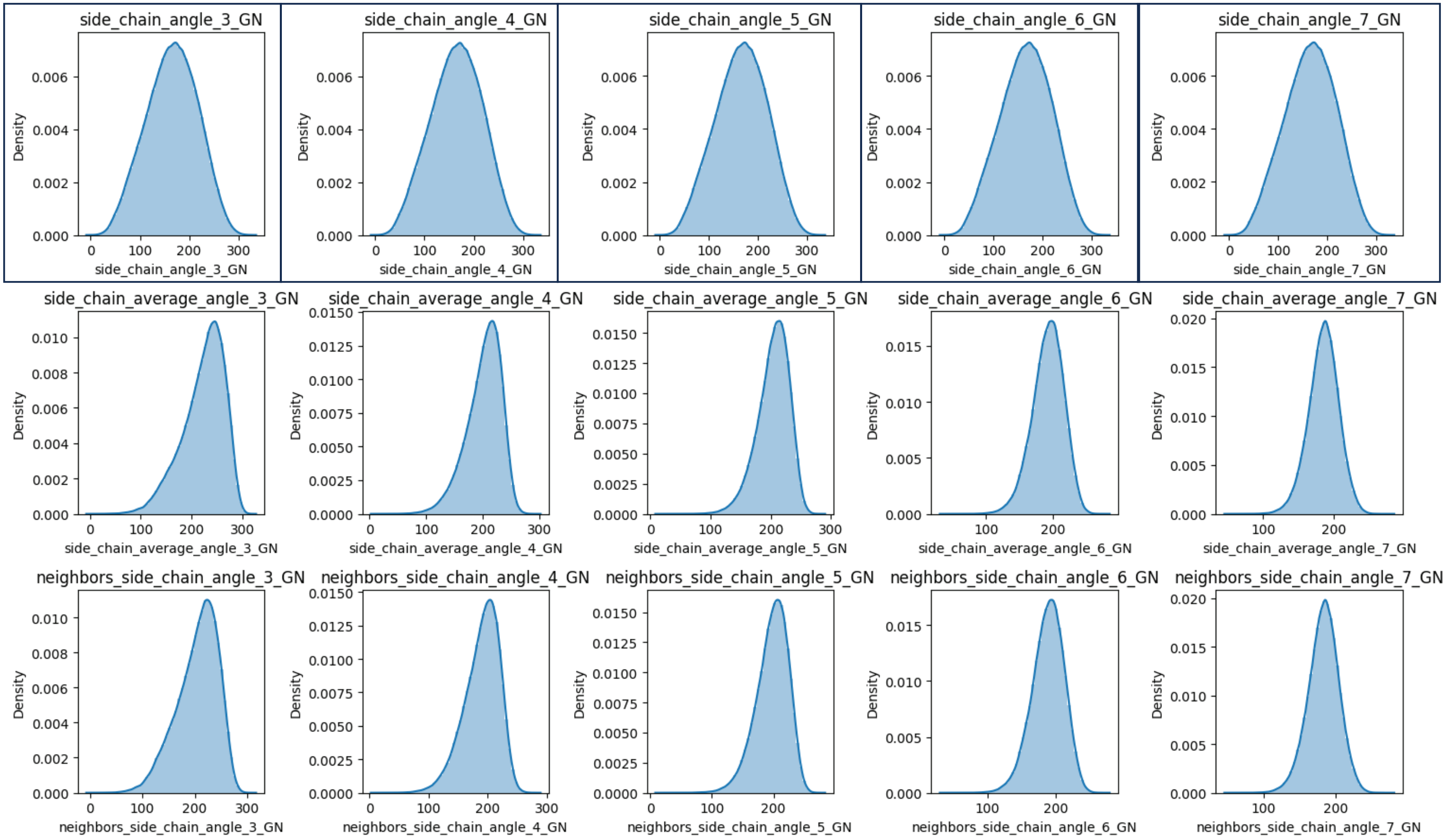

Cross_Presence_Order_SW

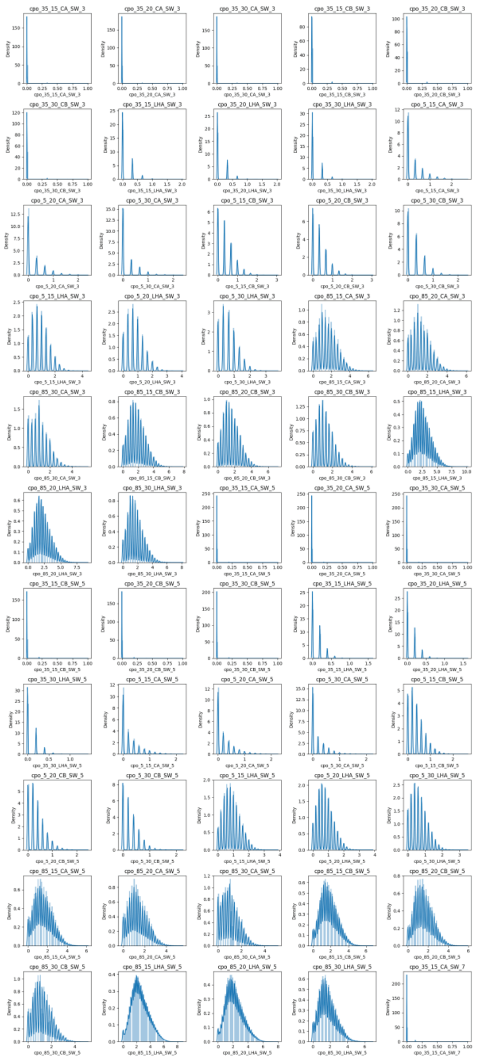

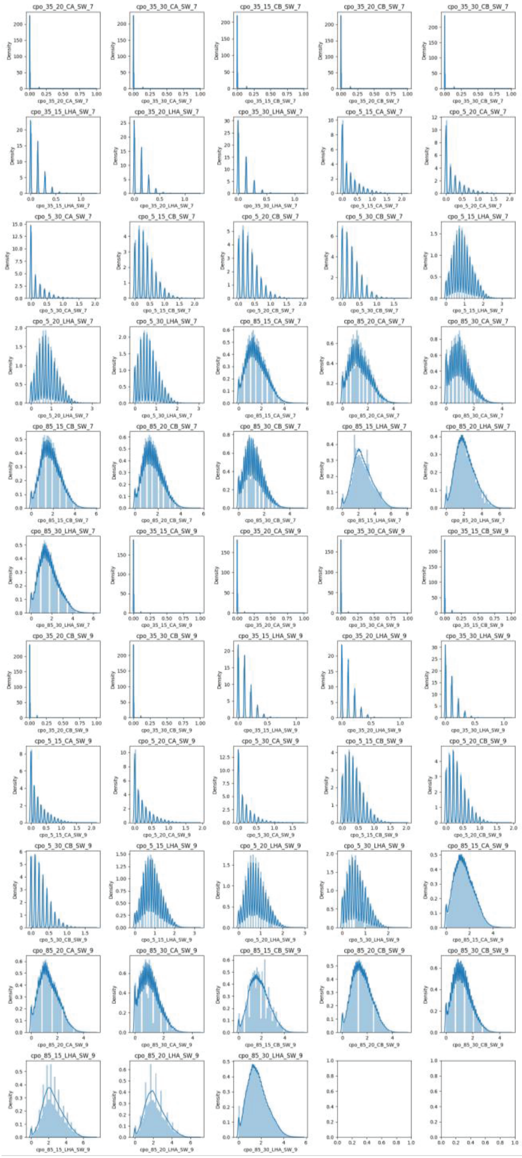

Side_Chain_Orientation_SW

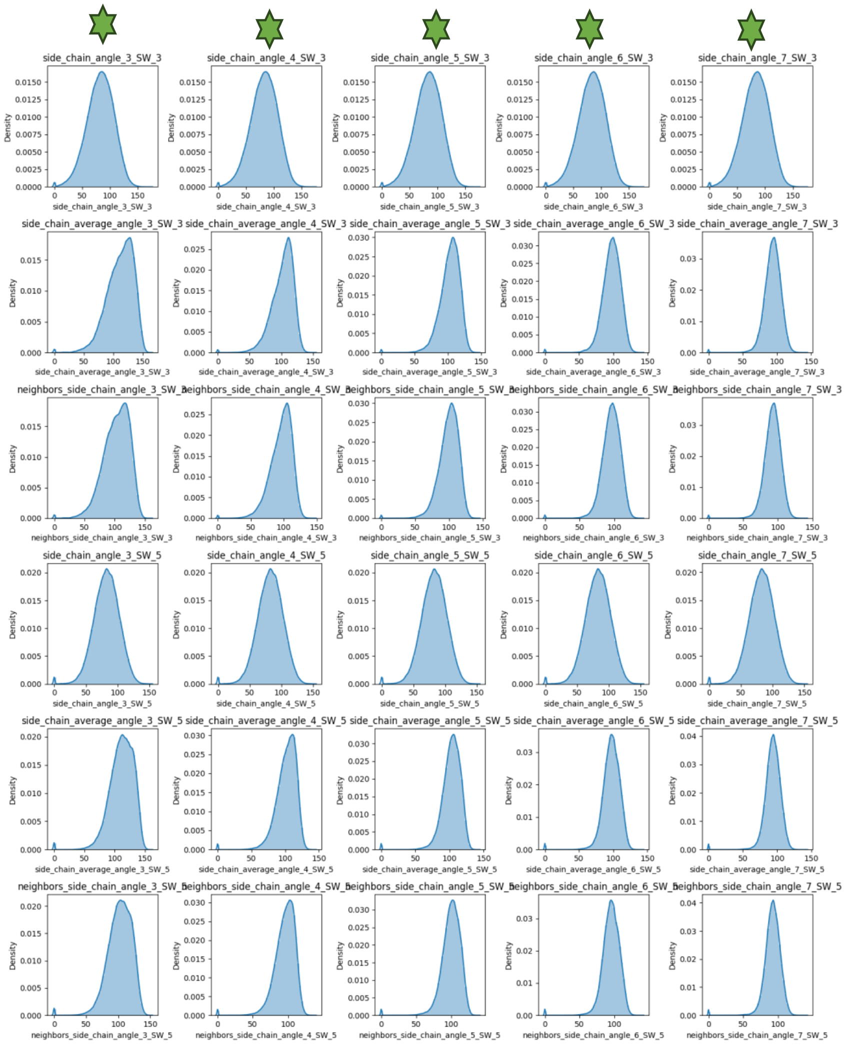

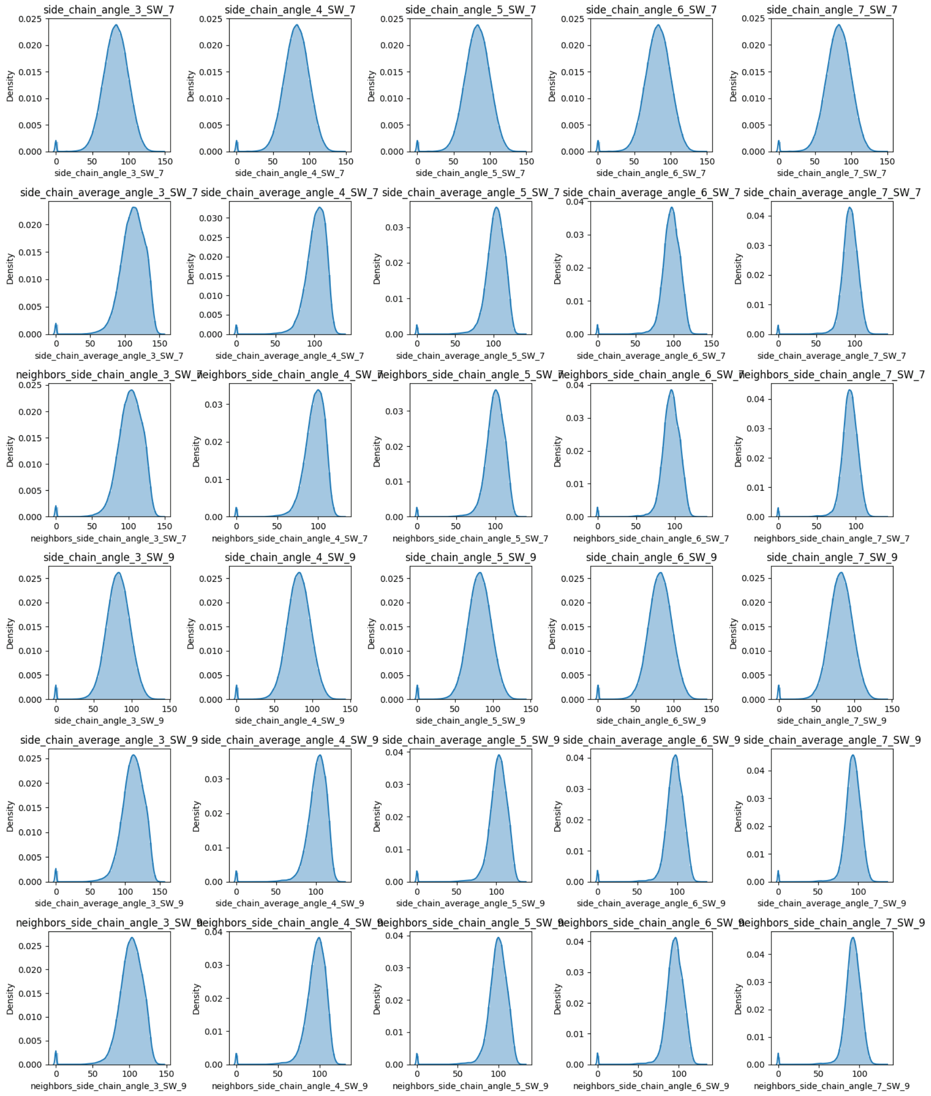

Side_Chain_Orientation_VD

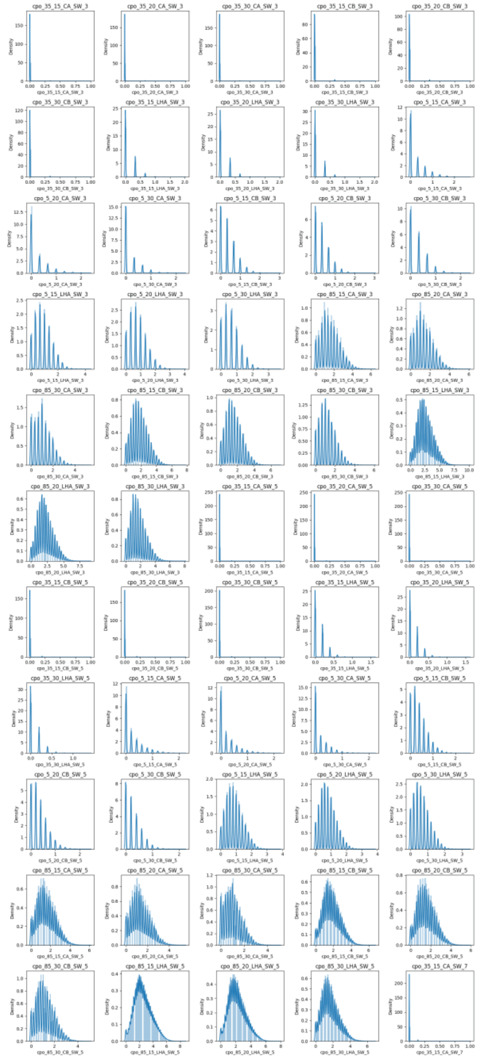

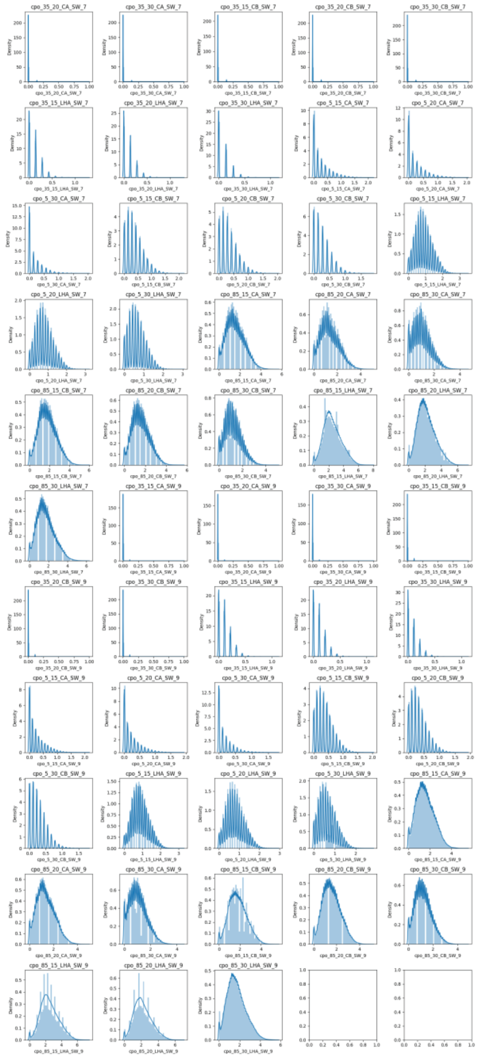

Cross_Presence_Order_WNA

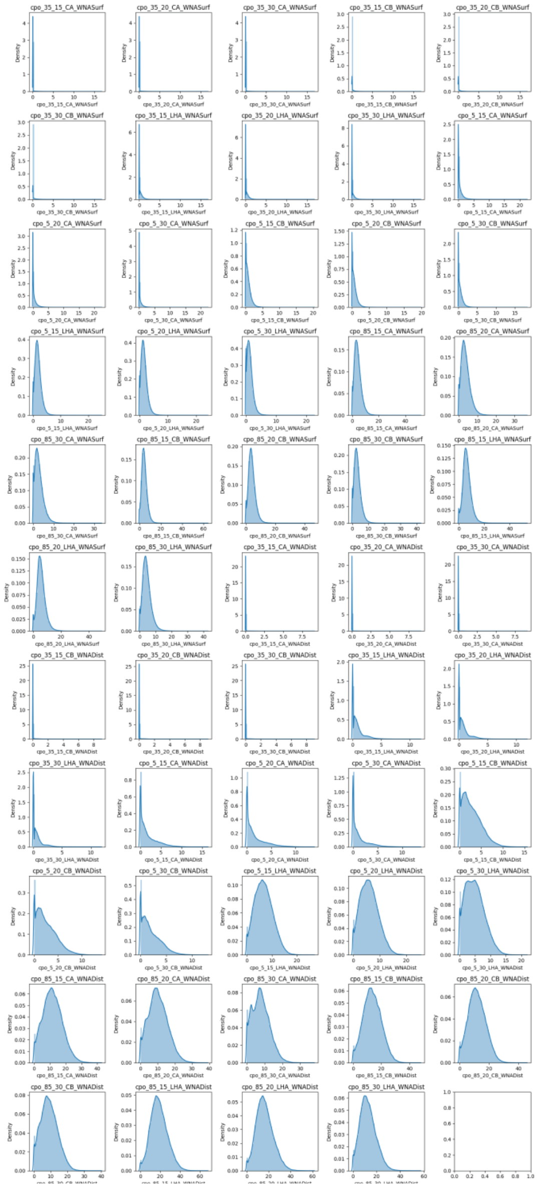

Side_Chain_Orientation_WNA

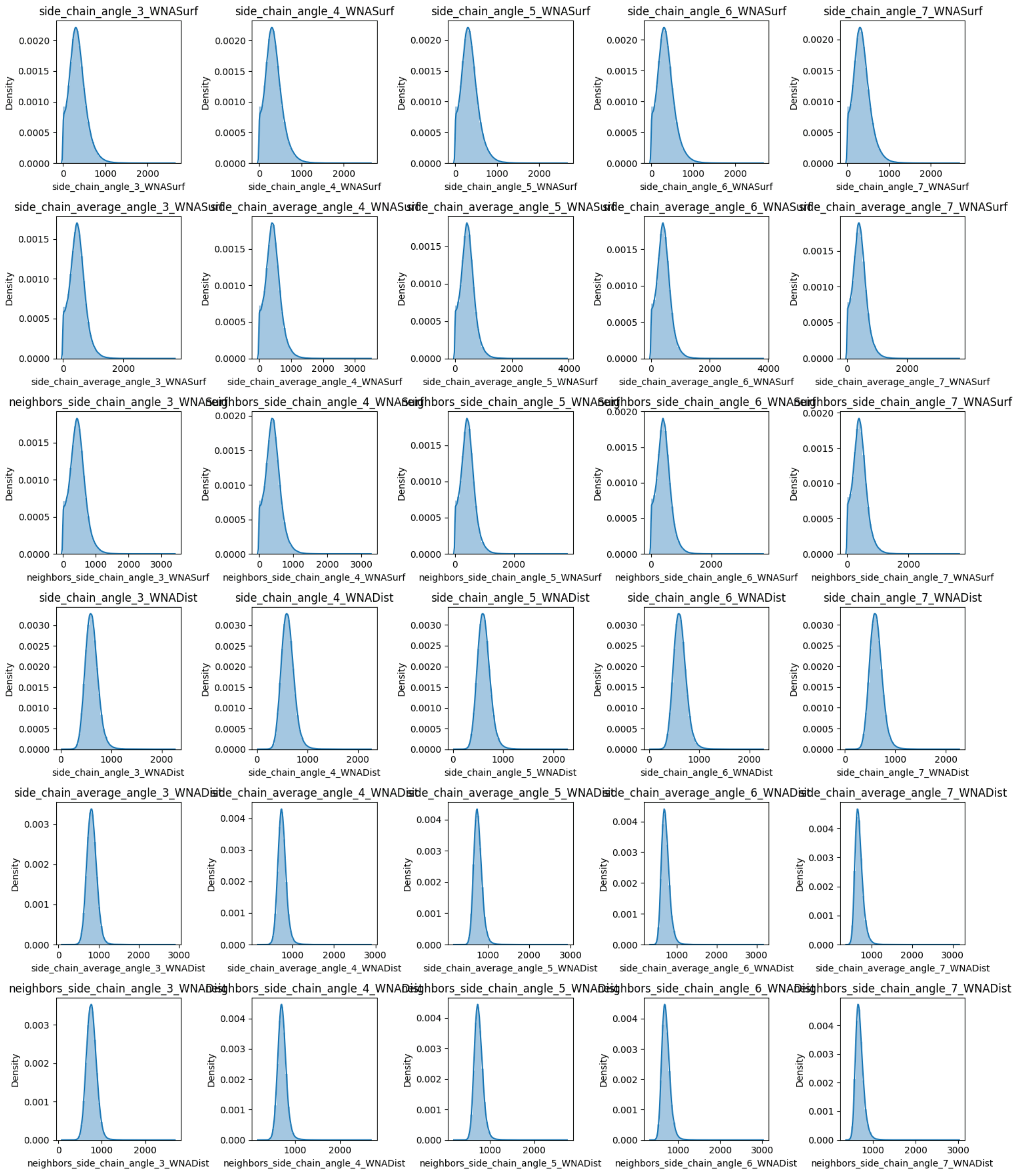

Curvature

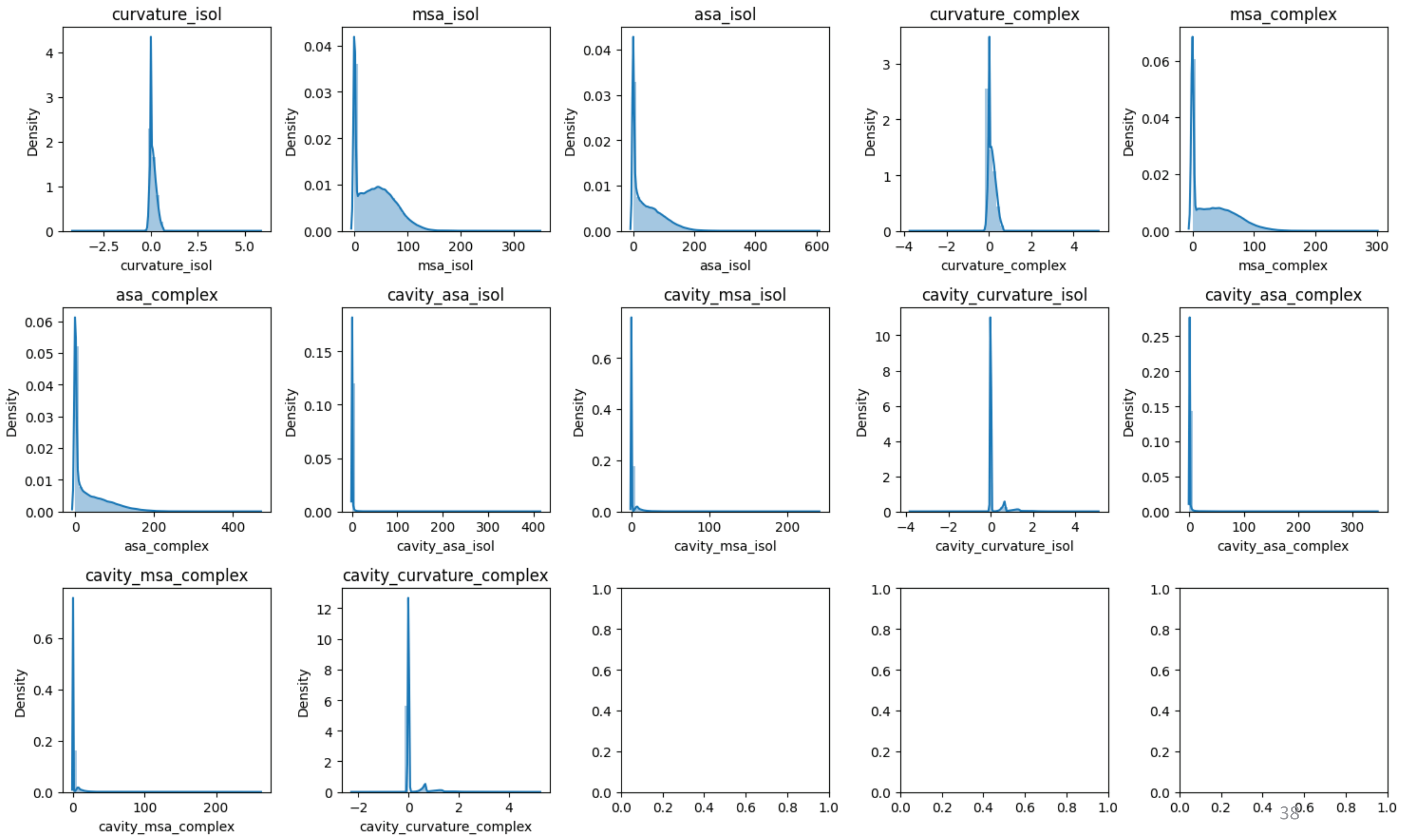
curvature_isol
msa_isol
asa_isol
curvature_complex
msa_complex
asa_complex
cavity_asa_isol
cavity_msa_isol
cavity_curvature_isol
cavity_asa_complex
cavity_msa_complex
cavity_curvature_complex
Density

Solvation

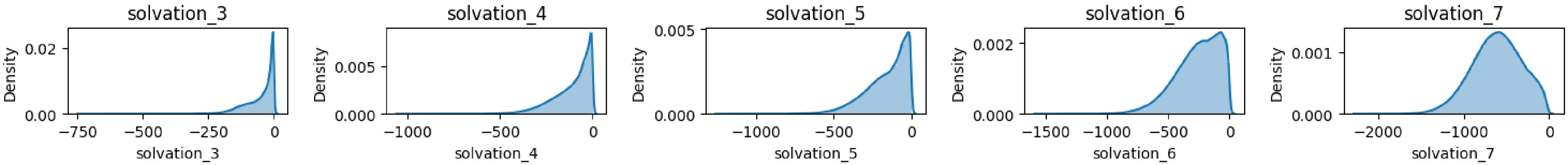

solvation_3
Density
0.02
0.00
−750
−500
−250
0
solvation_3
solvation_4
Density
0.005
0.000
−1000
−500
0
solvation_4
solvation_5
Density
0.005
0.000
−1000
−500
0
solvation_5
solvation_6
Density
0.002
0.000
−1500
−1000
−500
0
solvation_6
solvation_7
Density
0.001
0.000
−2000
−1000
0
solvation_7

Curvature_GN

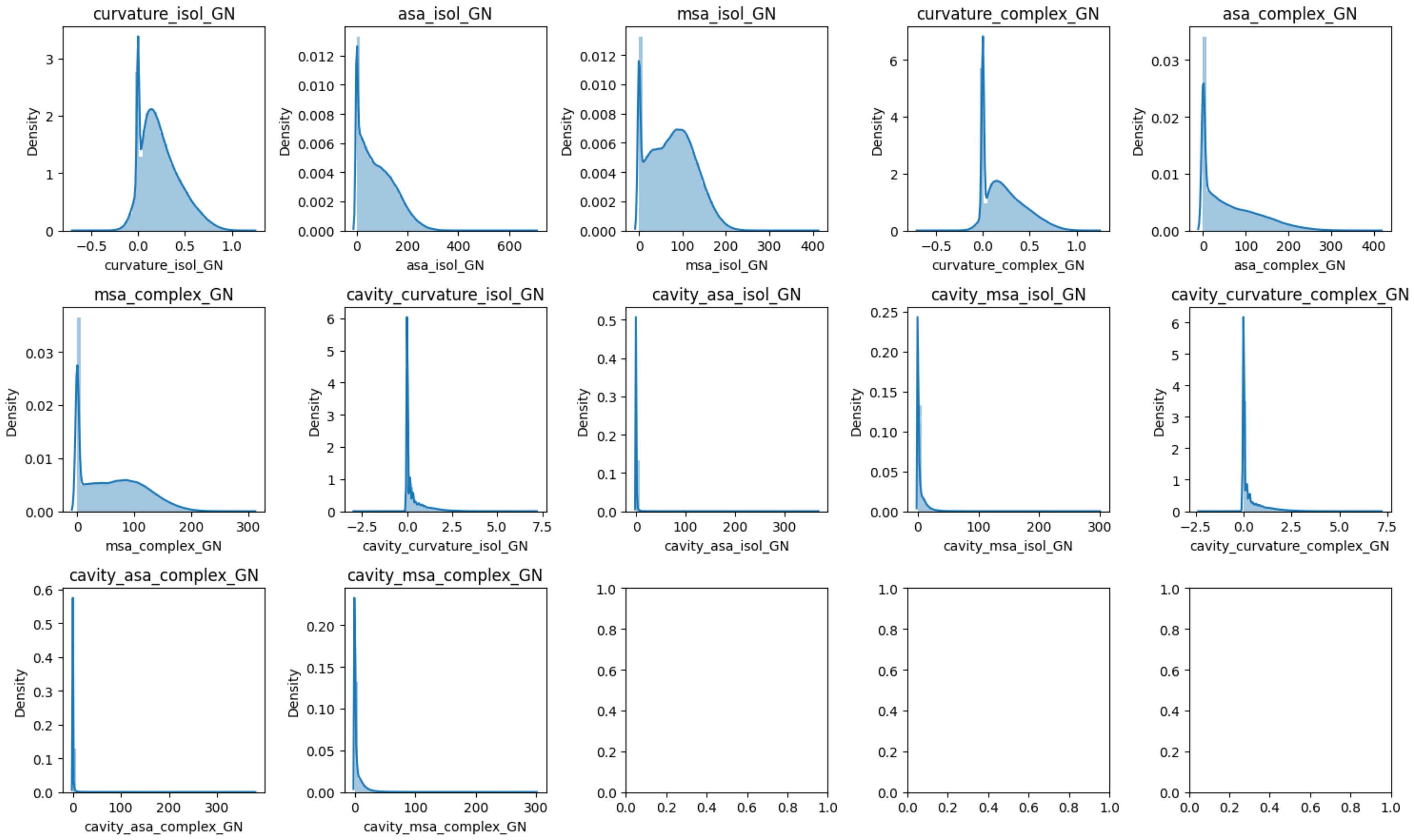

Solvation_GN

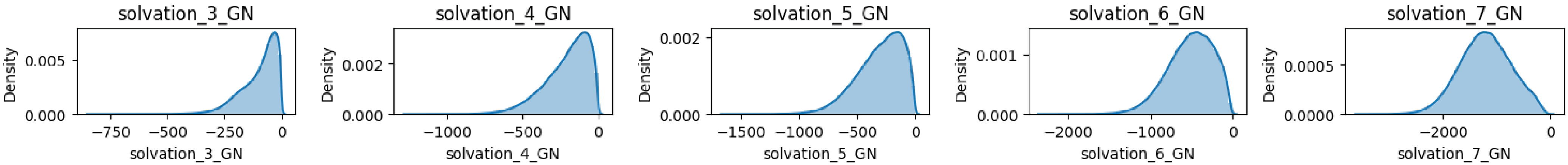

Solvation_SW

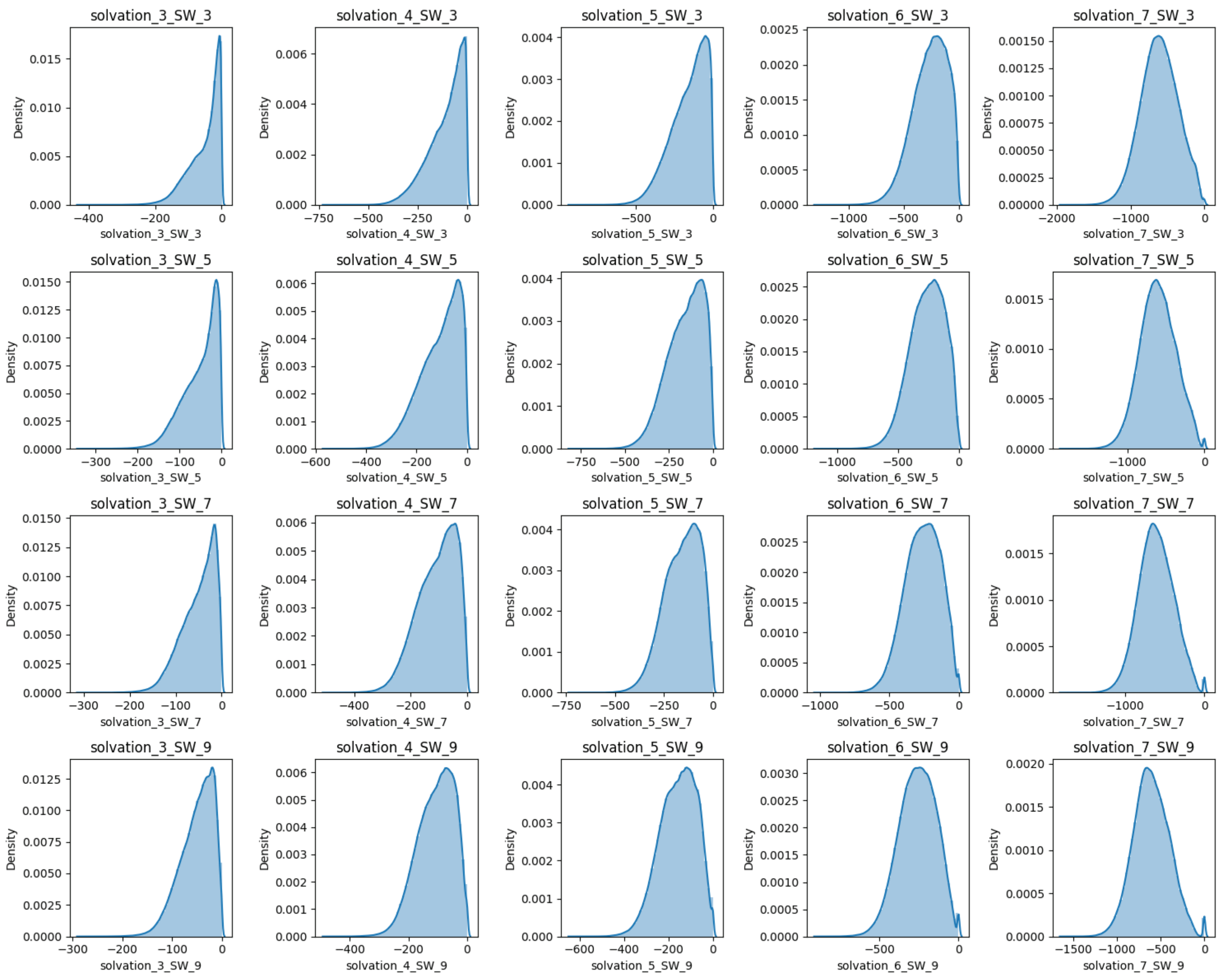

Curvature_SW

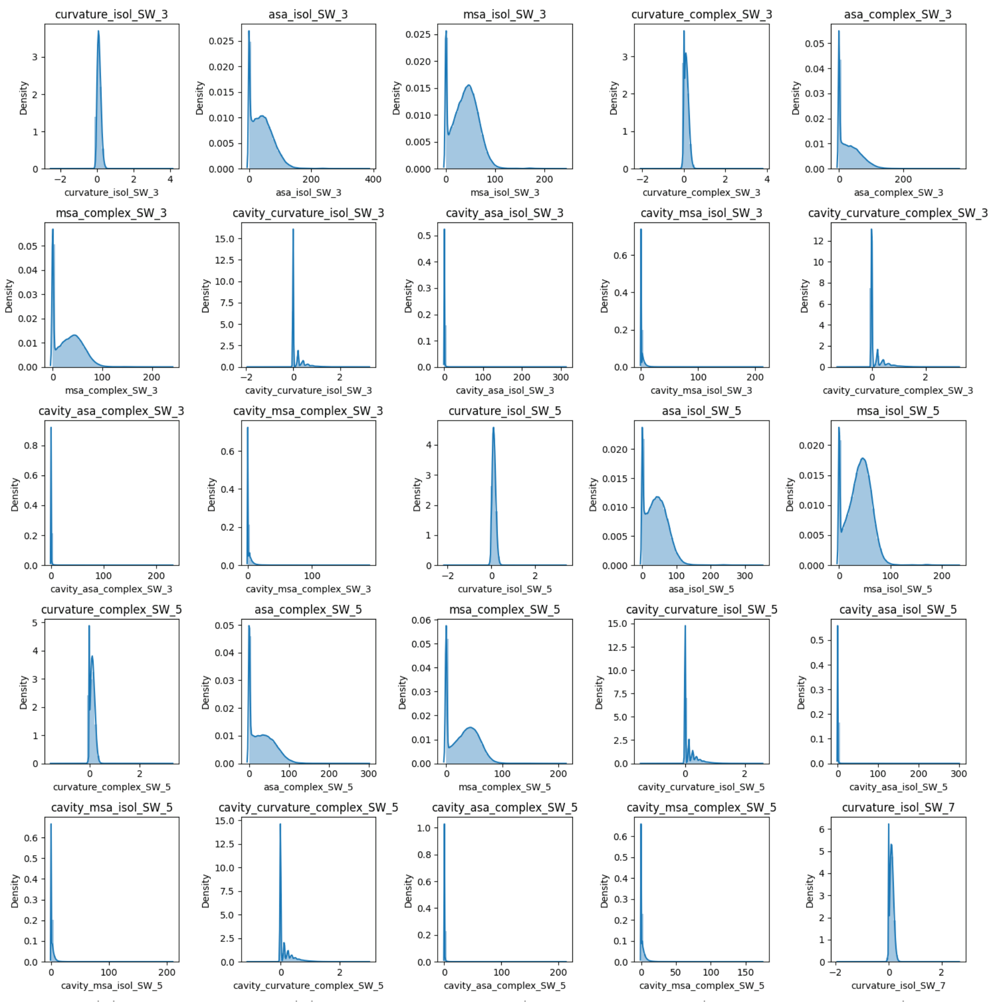

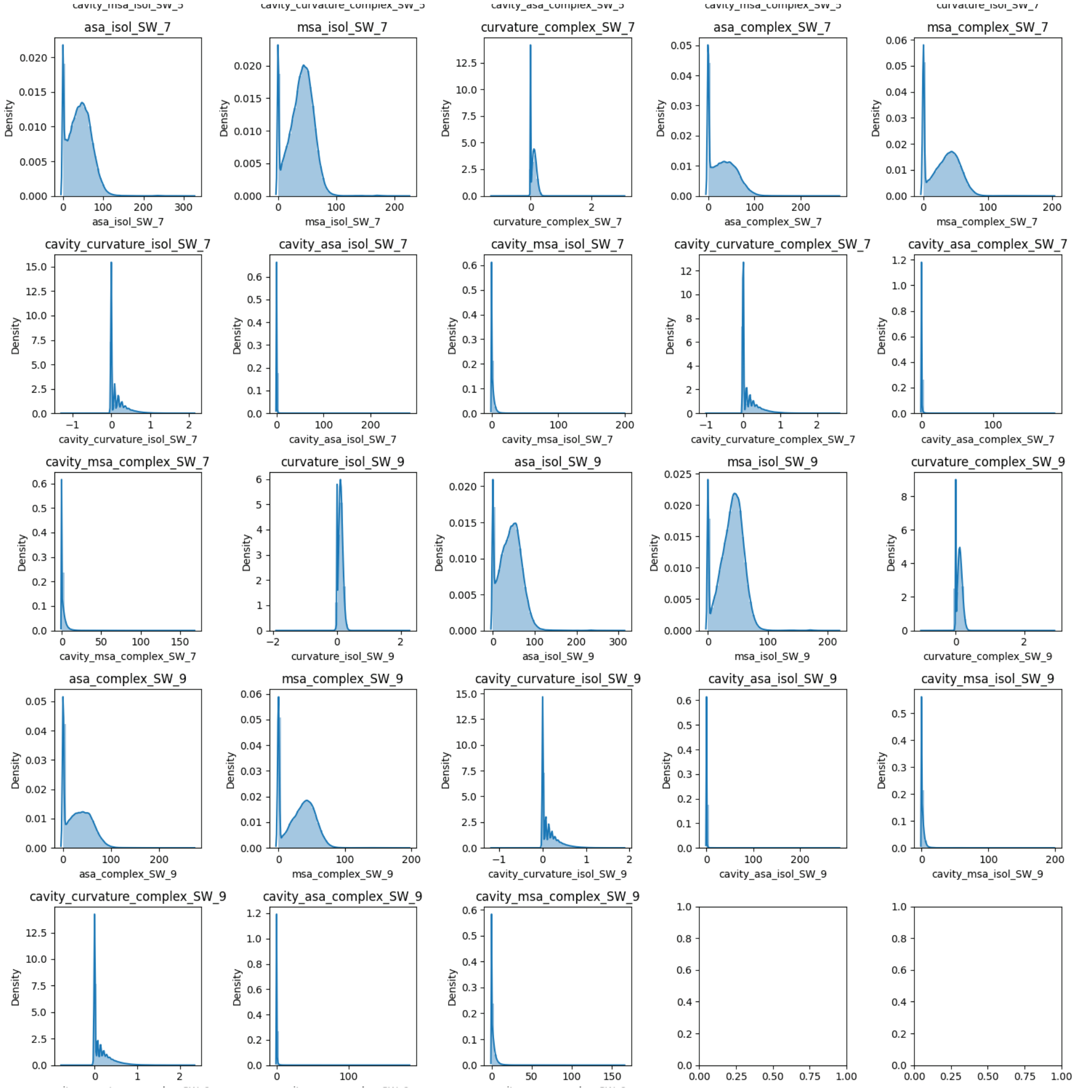

Solvation_VD

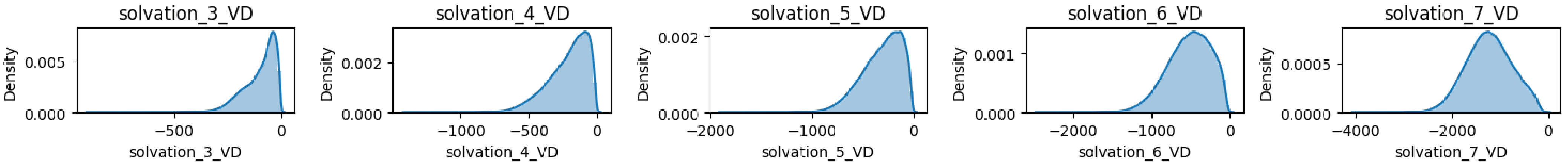

Curvature_VD

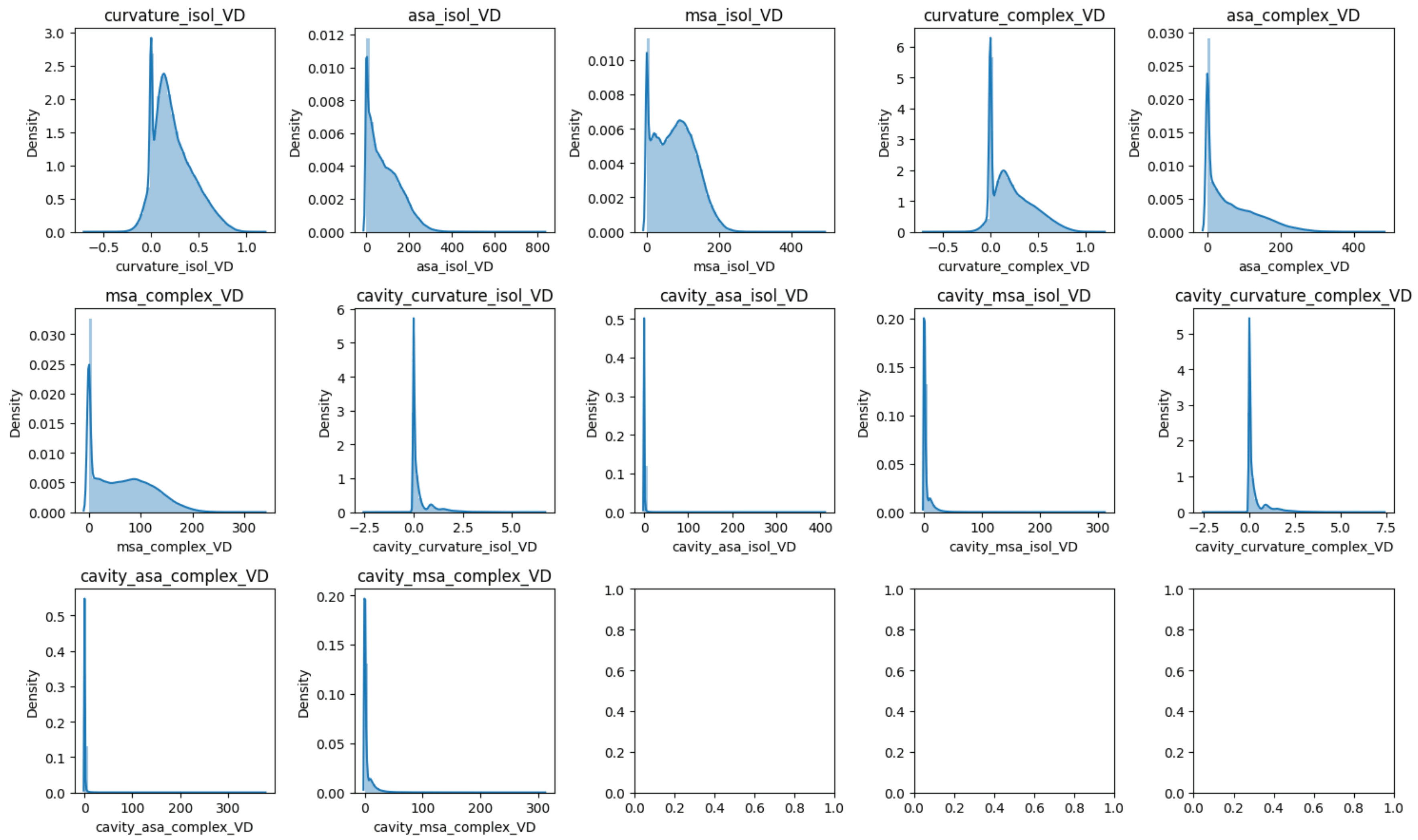

Solvation_WNA

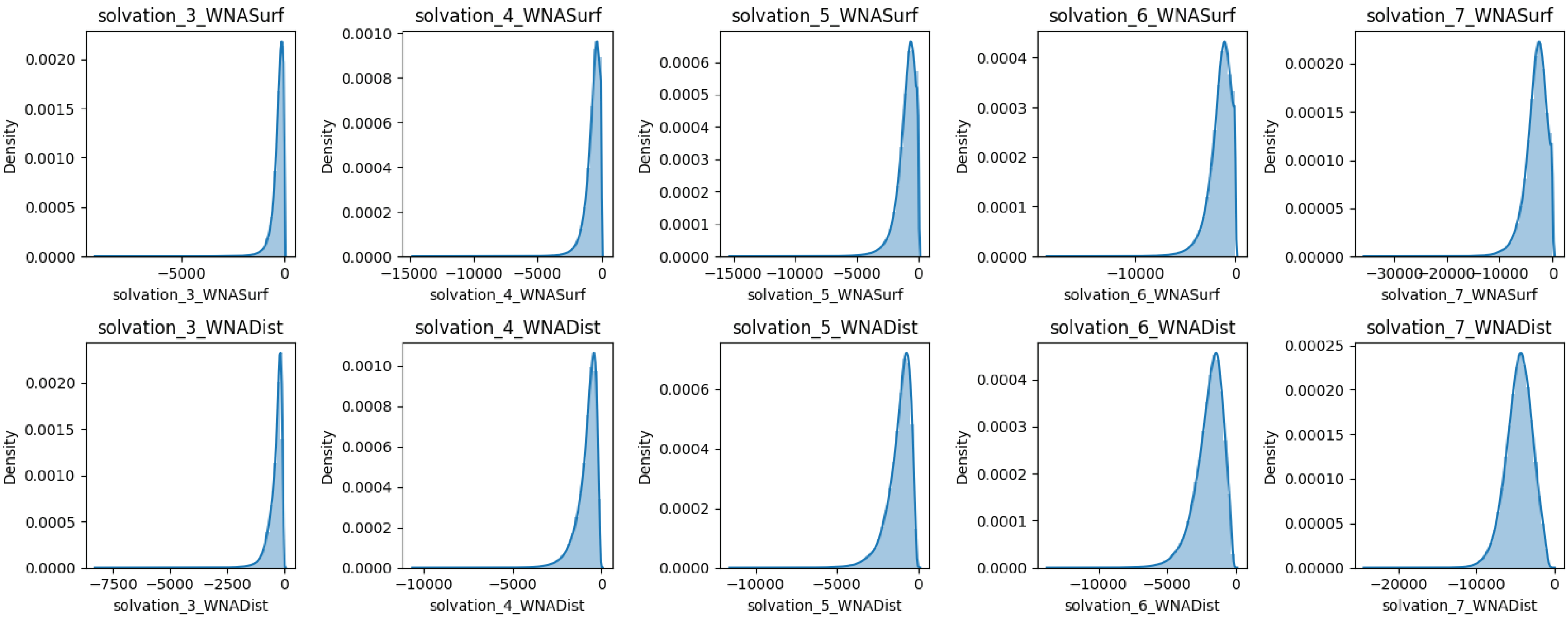

Curvature_WNA

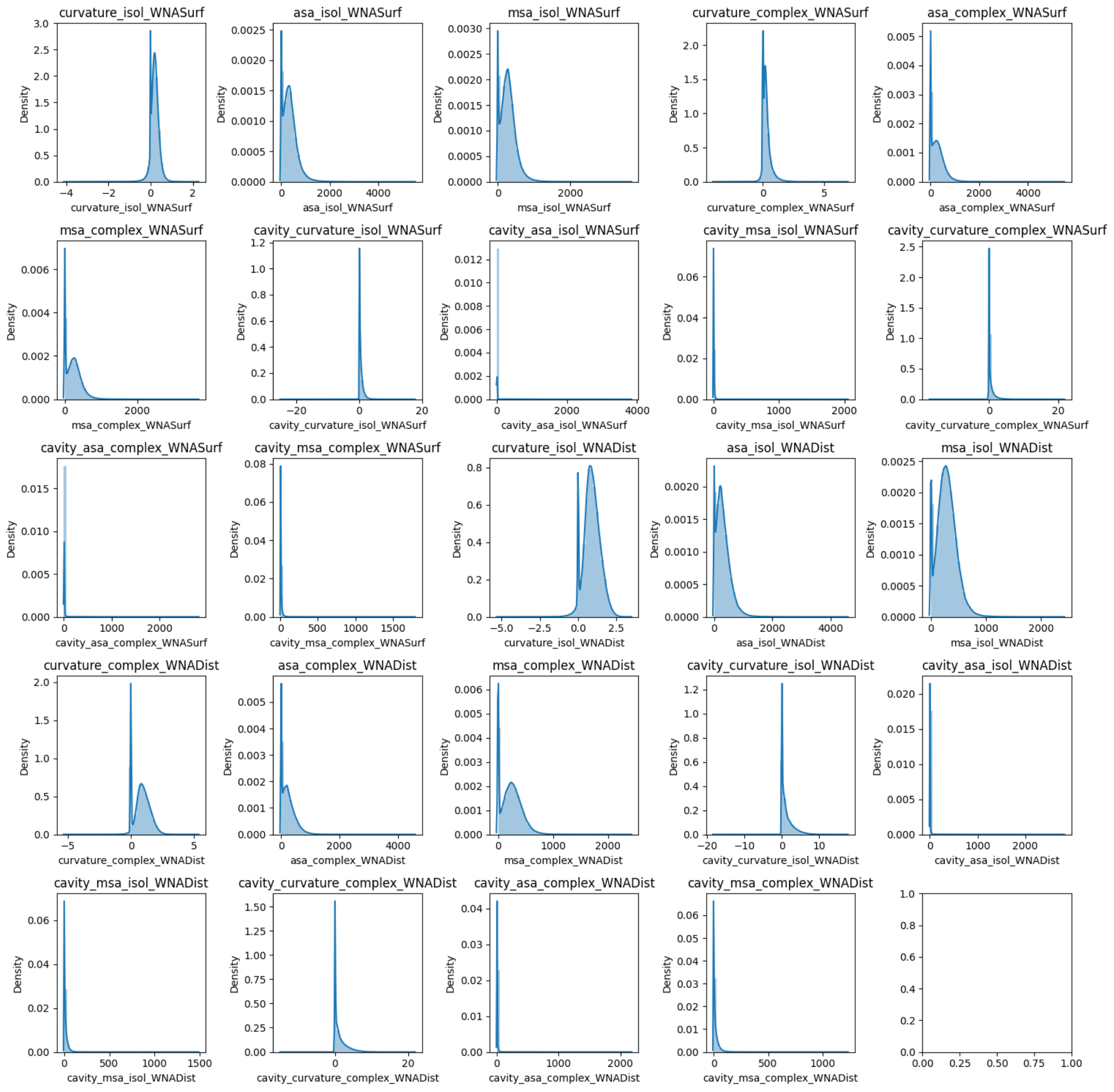

Space_Clash

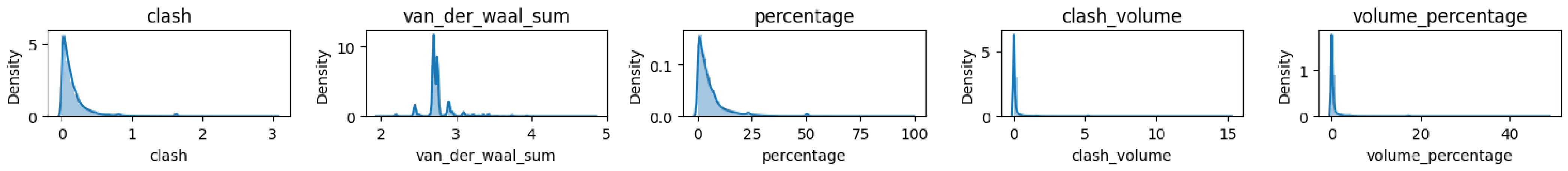

clash
Density
clash
van_der_waal_sum
Density
van_der_waal_sum
percentage
Density
percentage
clash_volume
Density
clash_volume
volume_percentage
Density
volume_percentage

Density_ Sponge

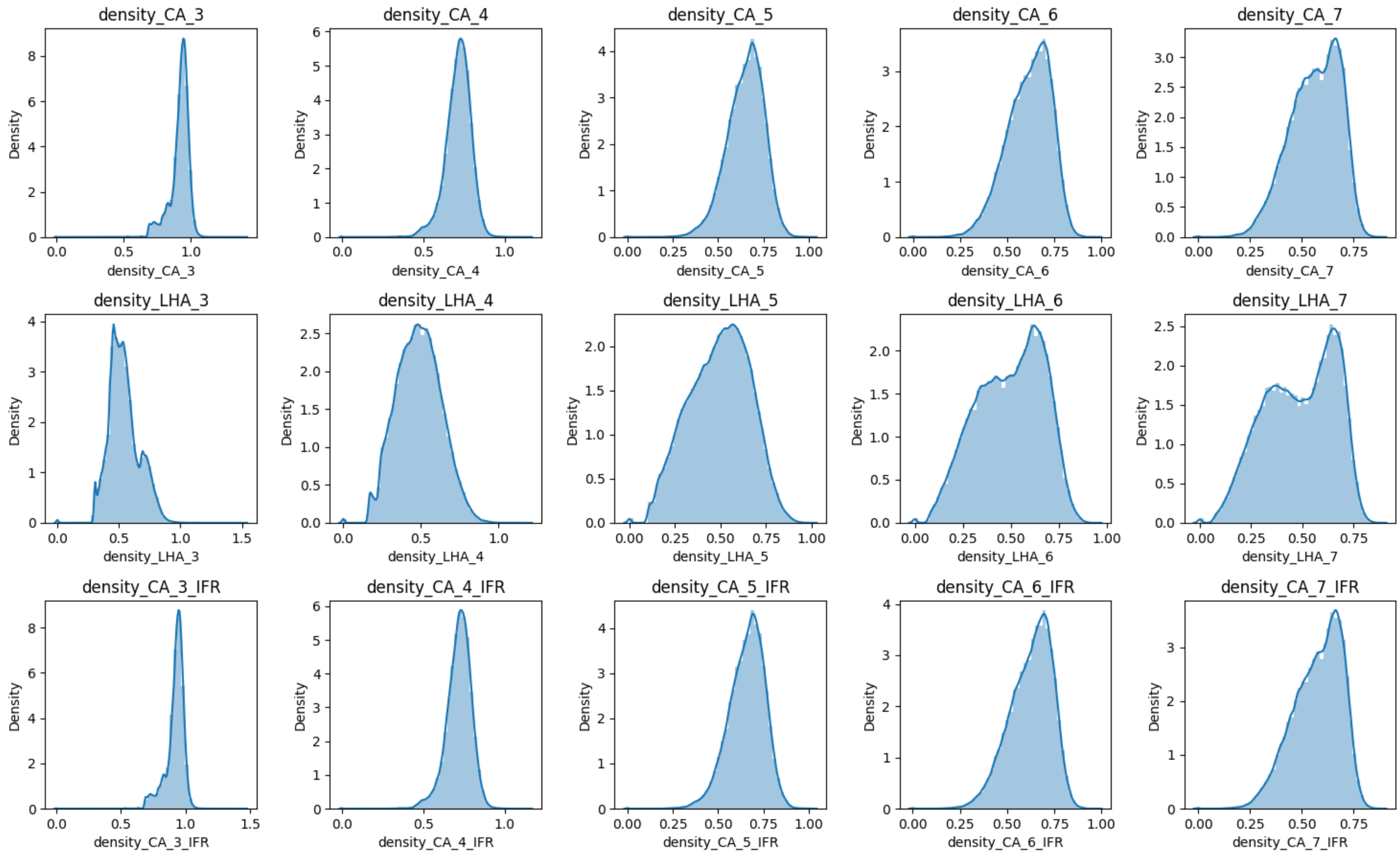

Density_ Sponge

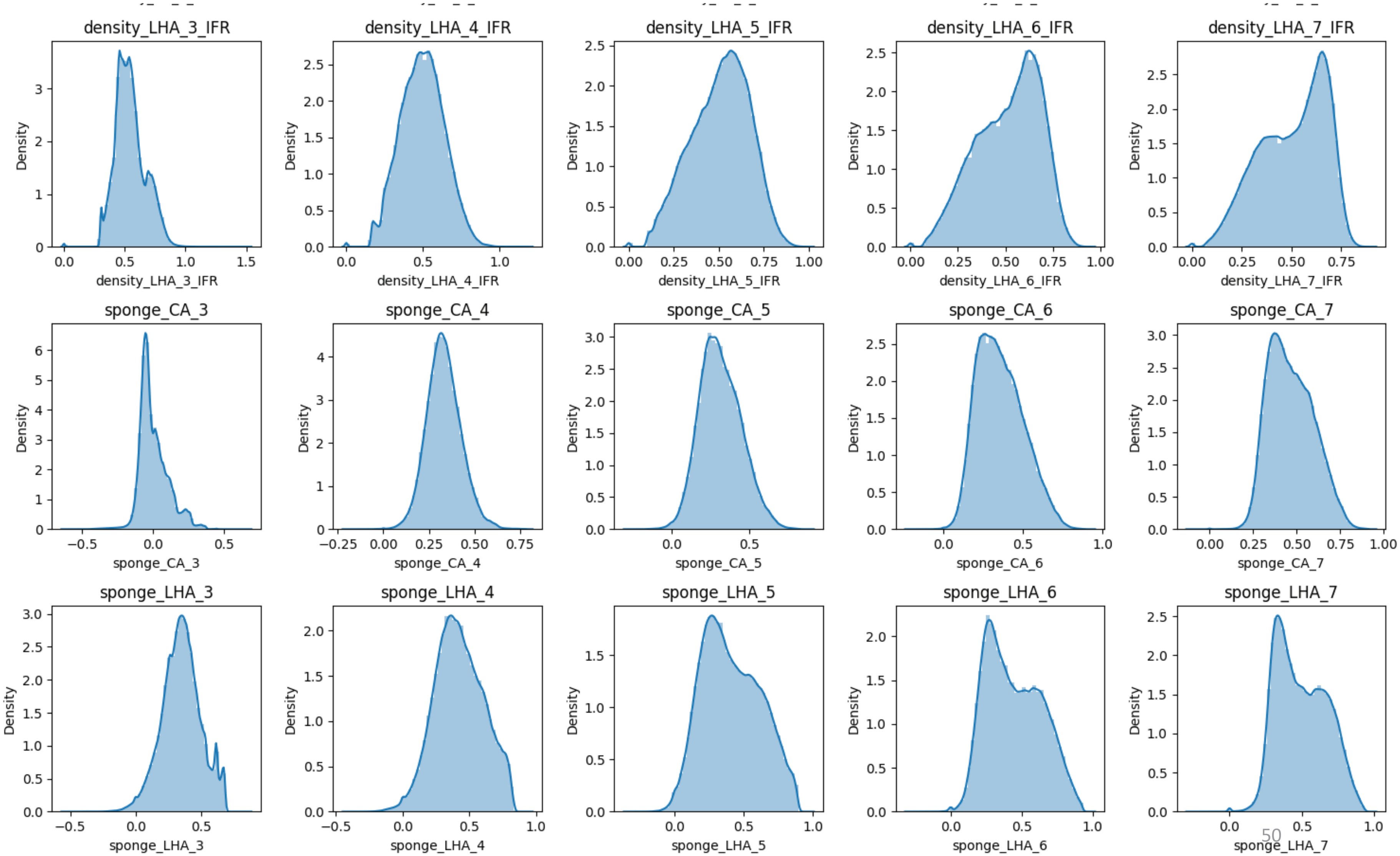

Density_ Sponge

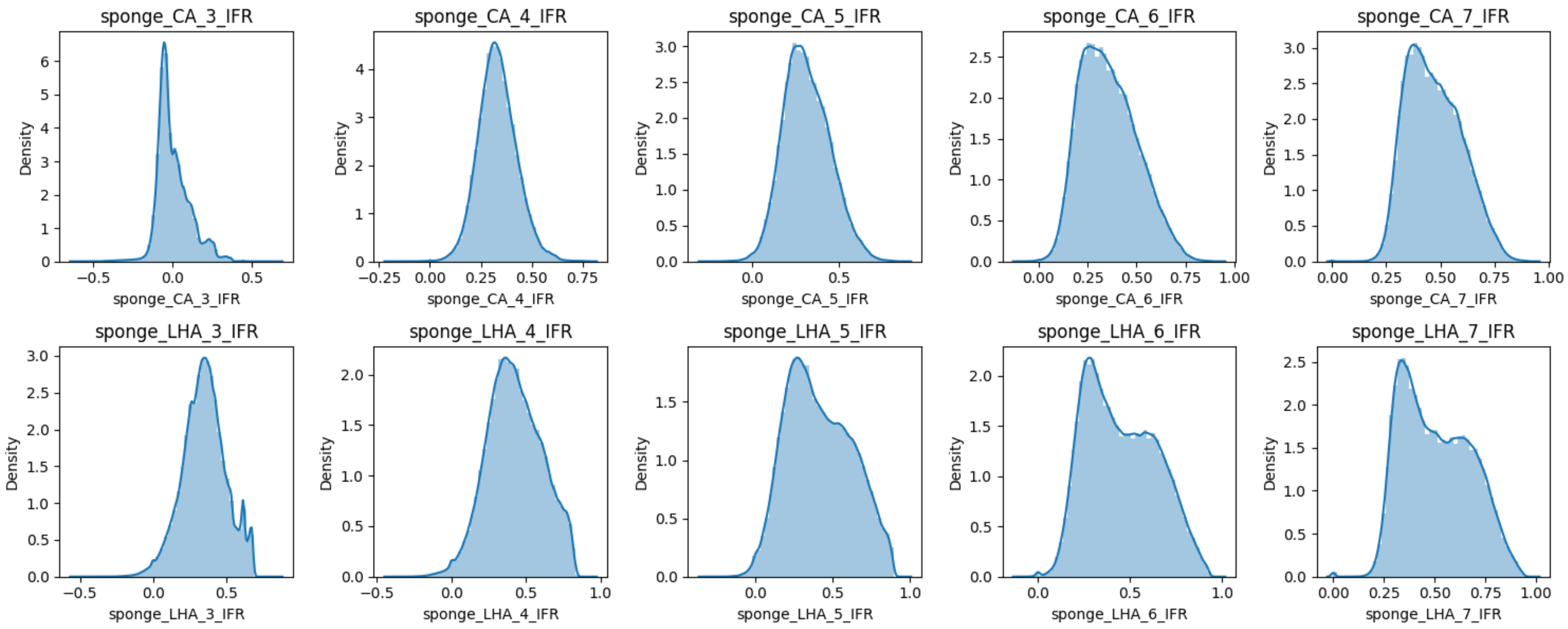

Correlation Matrix

Density_Sponge

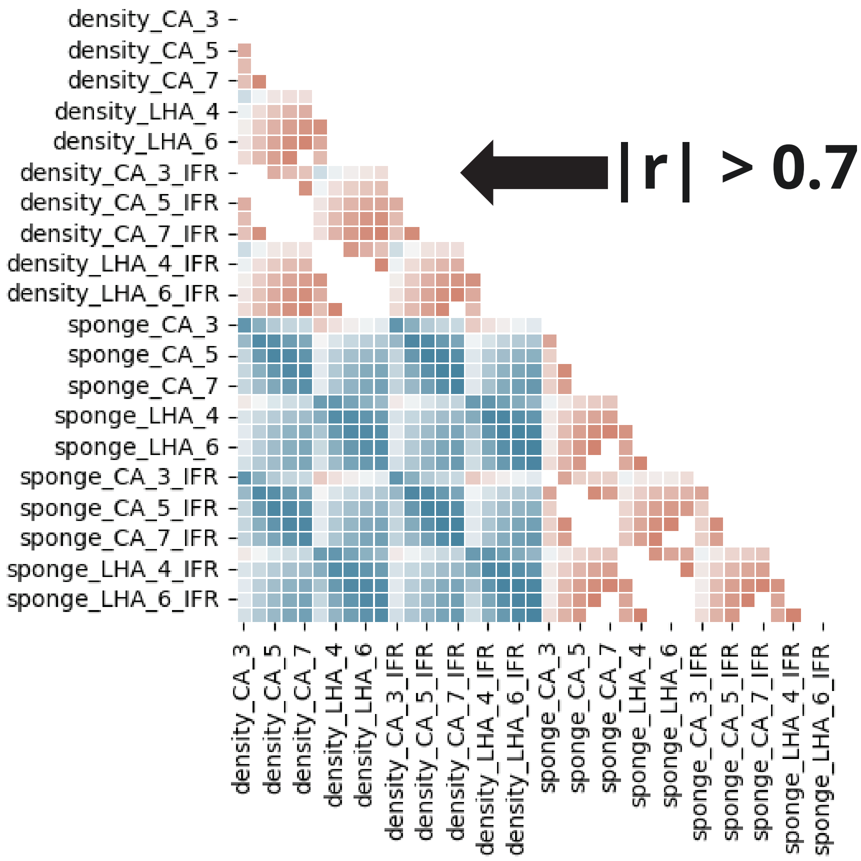

|r| > 0.7

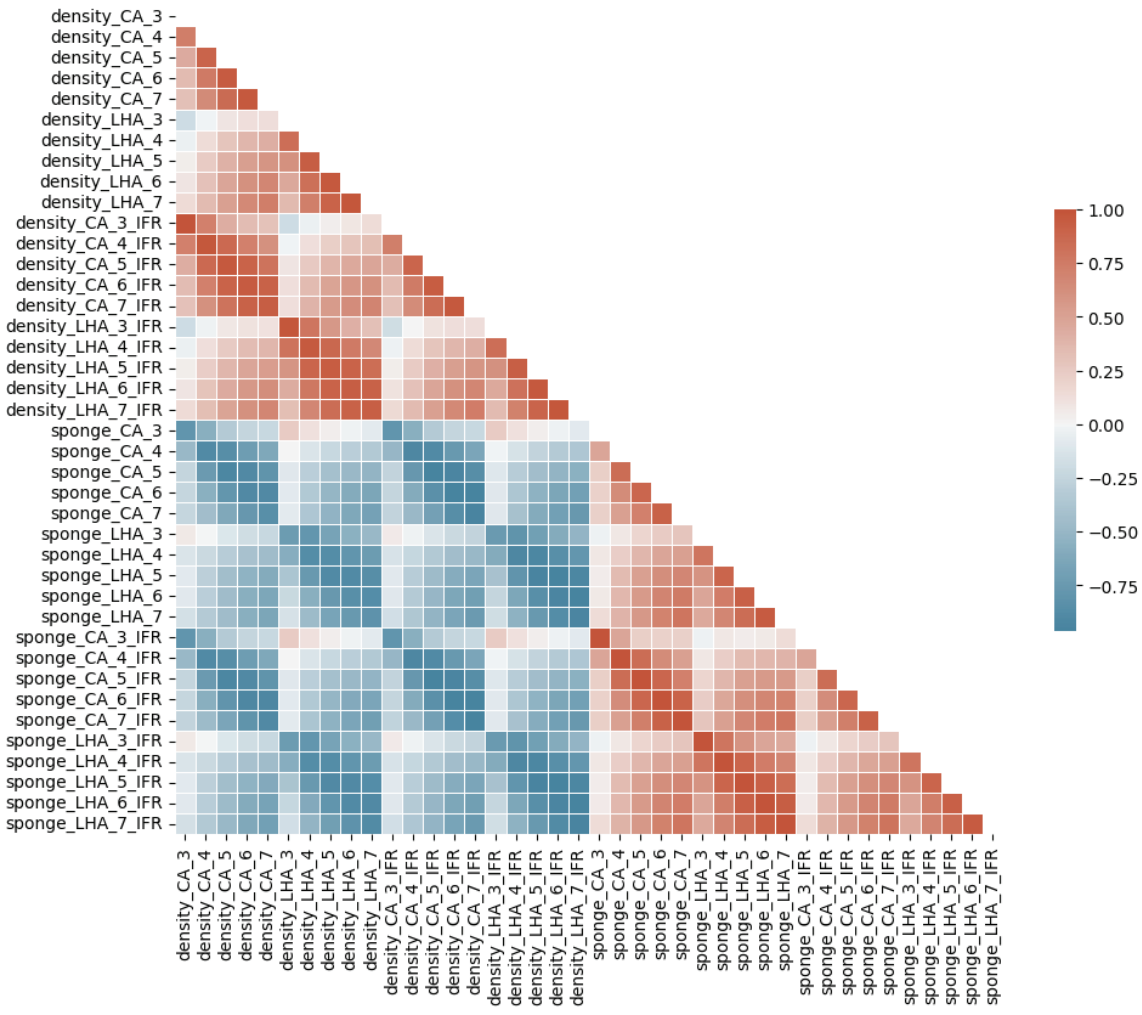

Sting_HSSP

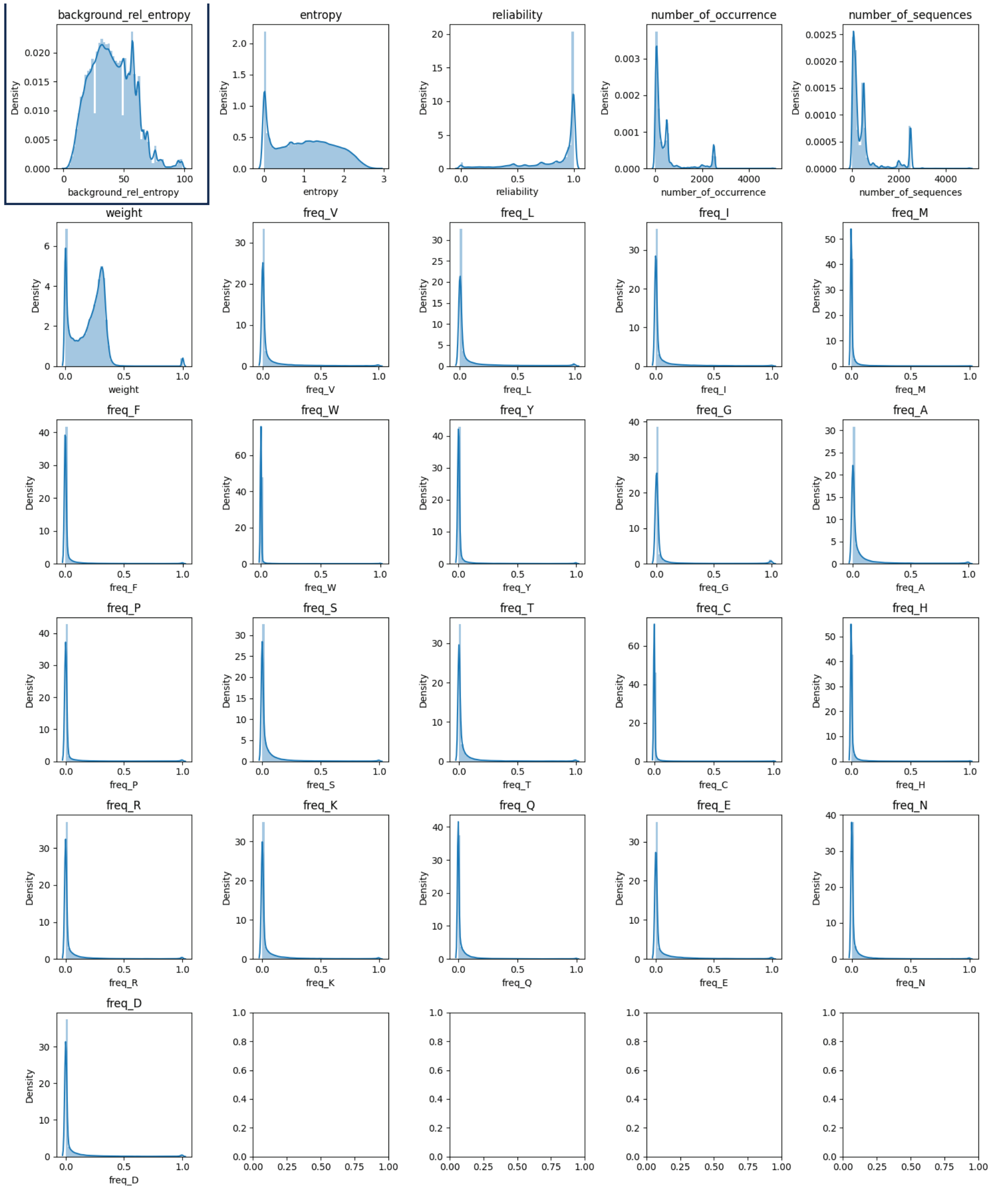
background_rel_entropy
entropy
reliability
number_of_occurrence
number_of_sequences
weight
freq_V
freq_L
freq_I
freq_M
freq_F
freq_W
freq_Y
freq_G
freq_A
freq_P
freq_S
freq_T
freq_C
freq_H
freq_R
freq_K
freq_Q
freq_E
freq_N
freq_D
Density

Stride

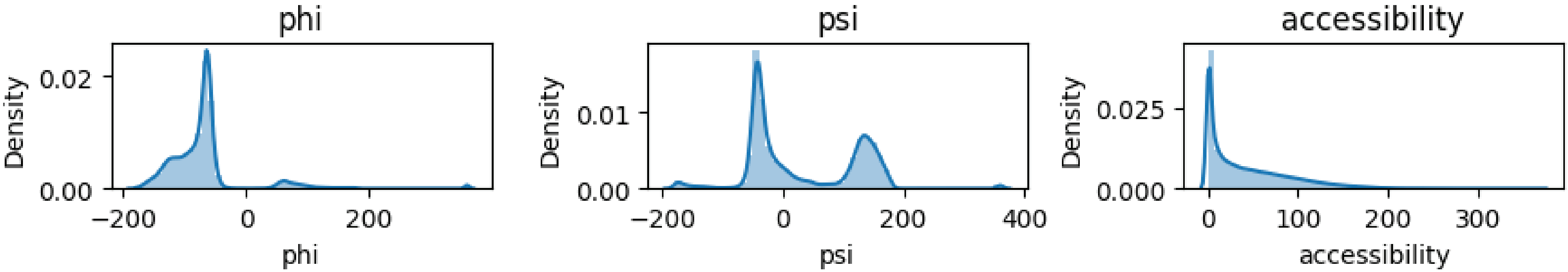

phi
Density
0.02
0.00
−200
0
200
phi
psi
Density
0.01
0.00
−200
0
200
400
psi
accessibility
Density
0.025
0.000
0
100
200
300
accessibility

Density_Sponge_SW

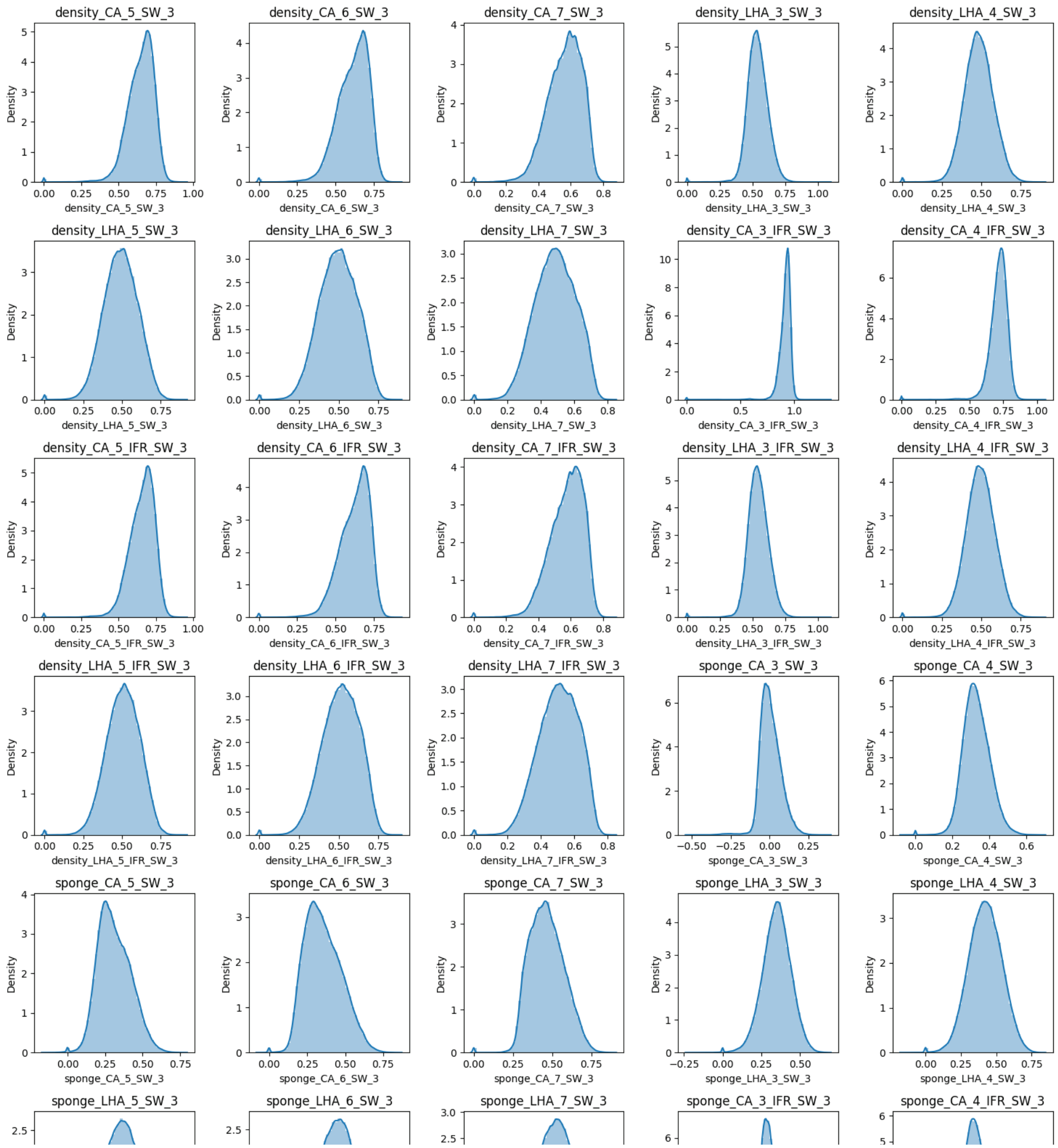

Density_Sponge_SW

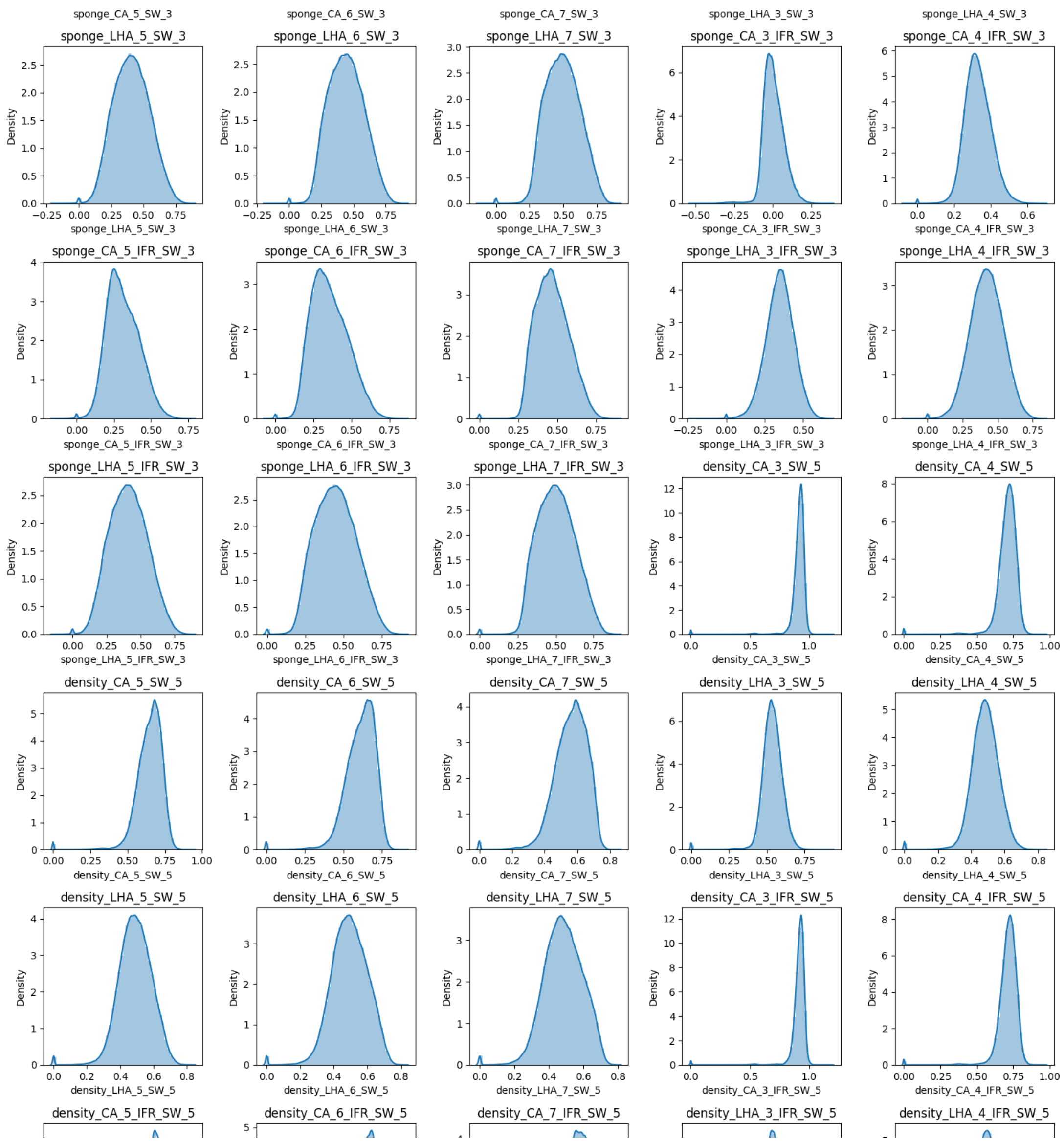

Density_Sponge_SW

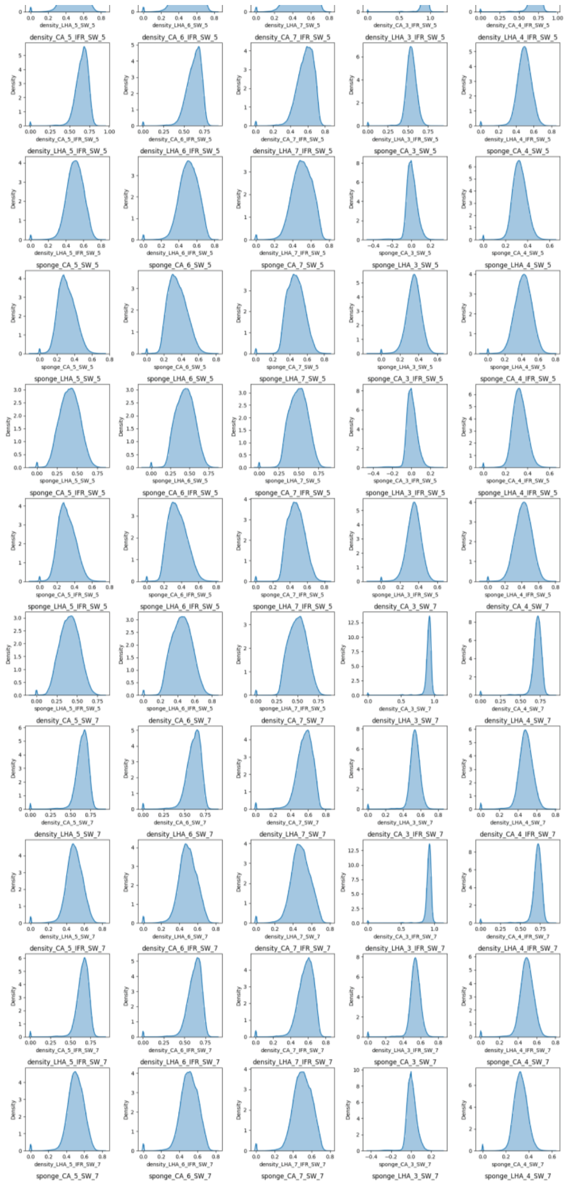

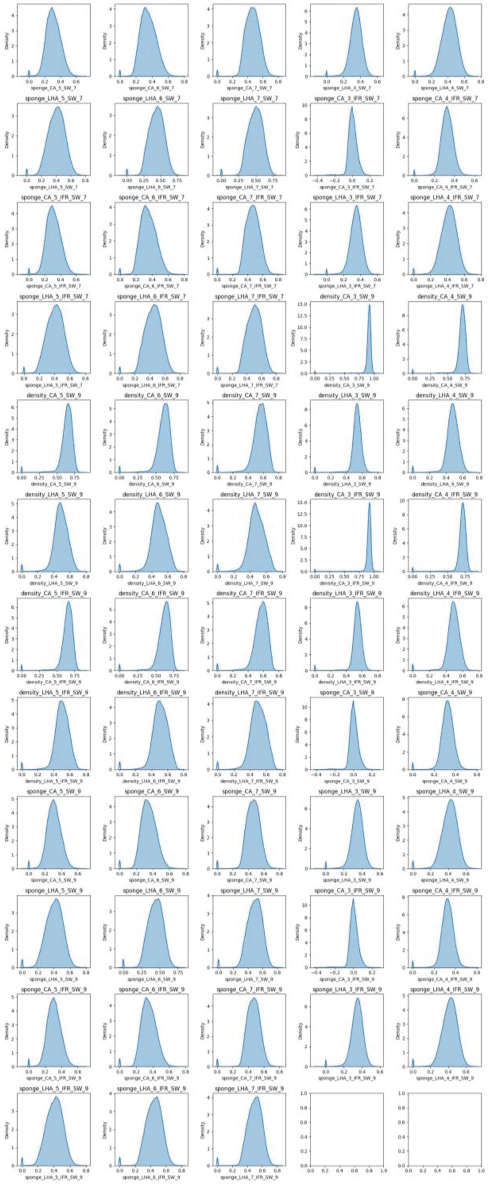

Density_Sponge_GN

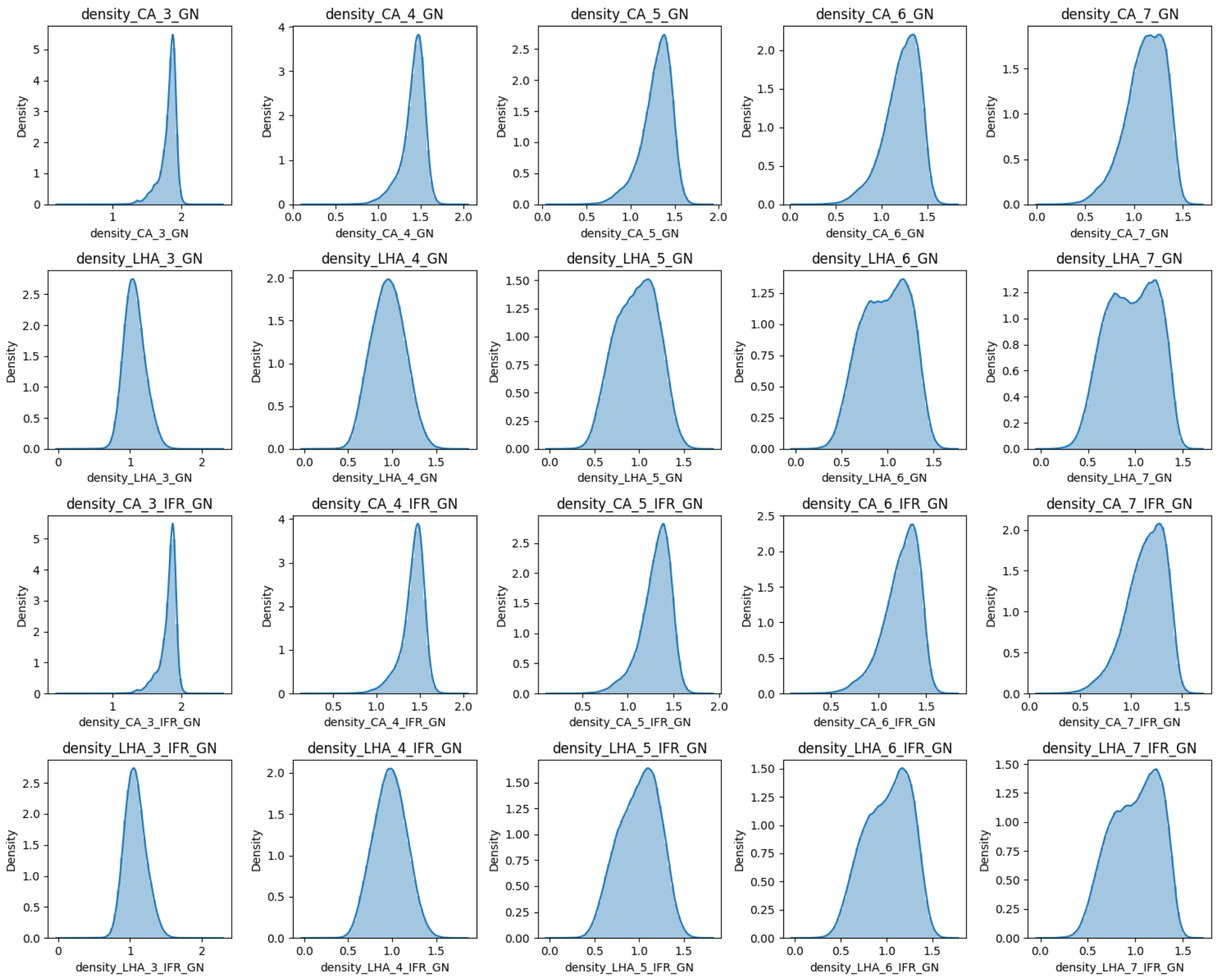

Density_Sponge_GN

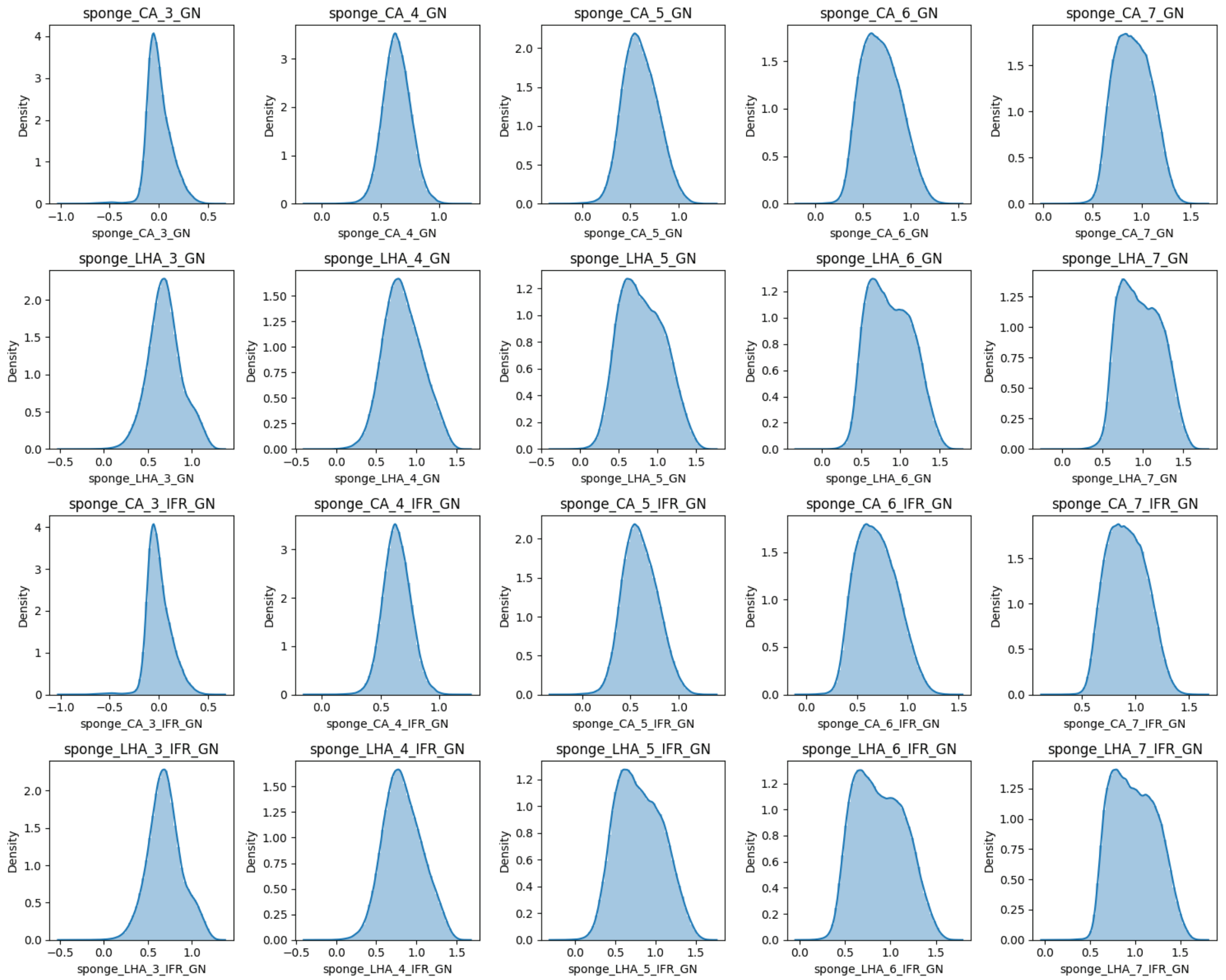

Correlation Matrix

Density_Sponge_GN

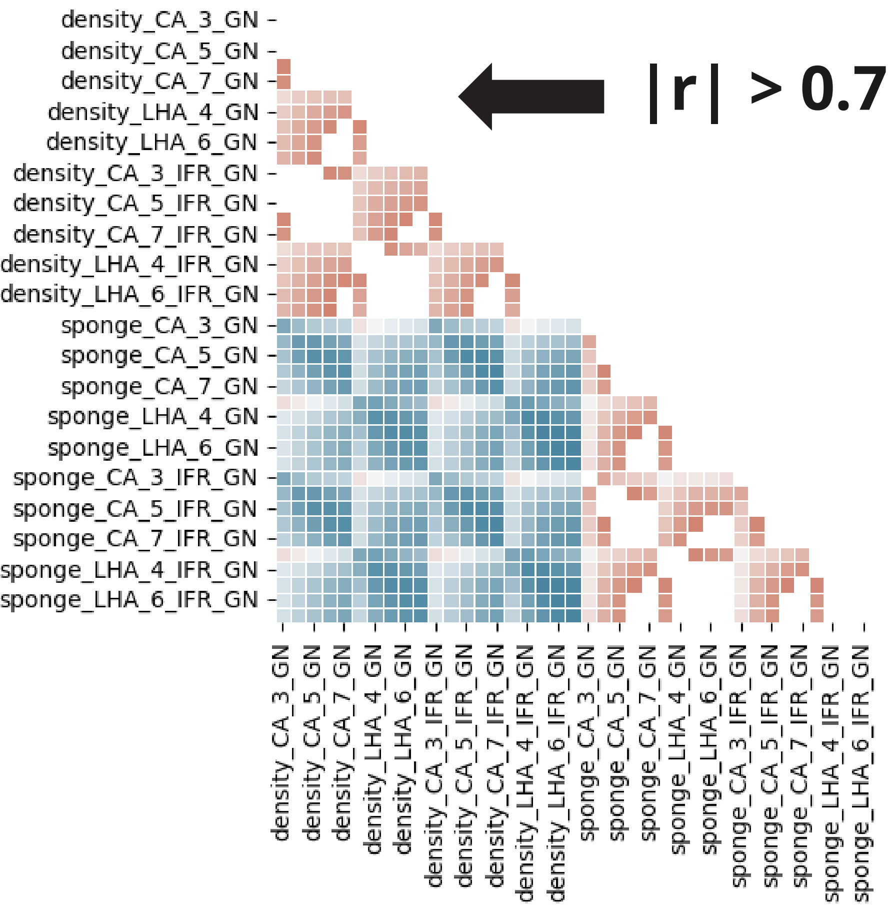

|r| > 0.7

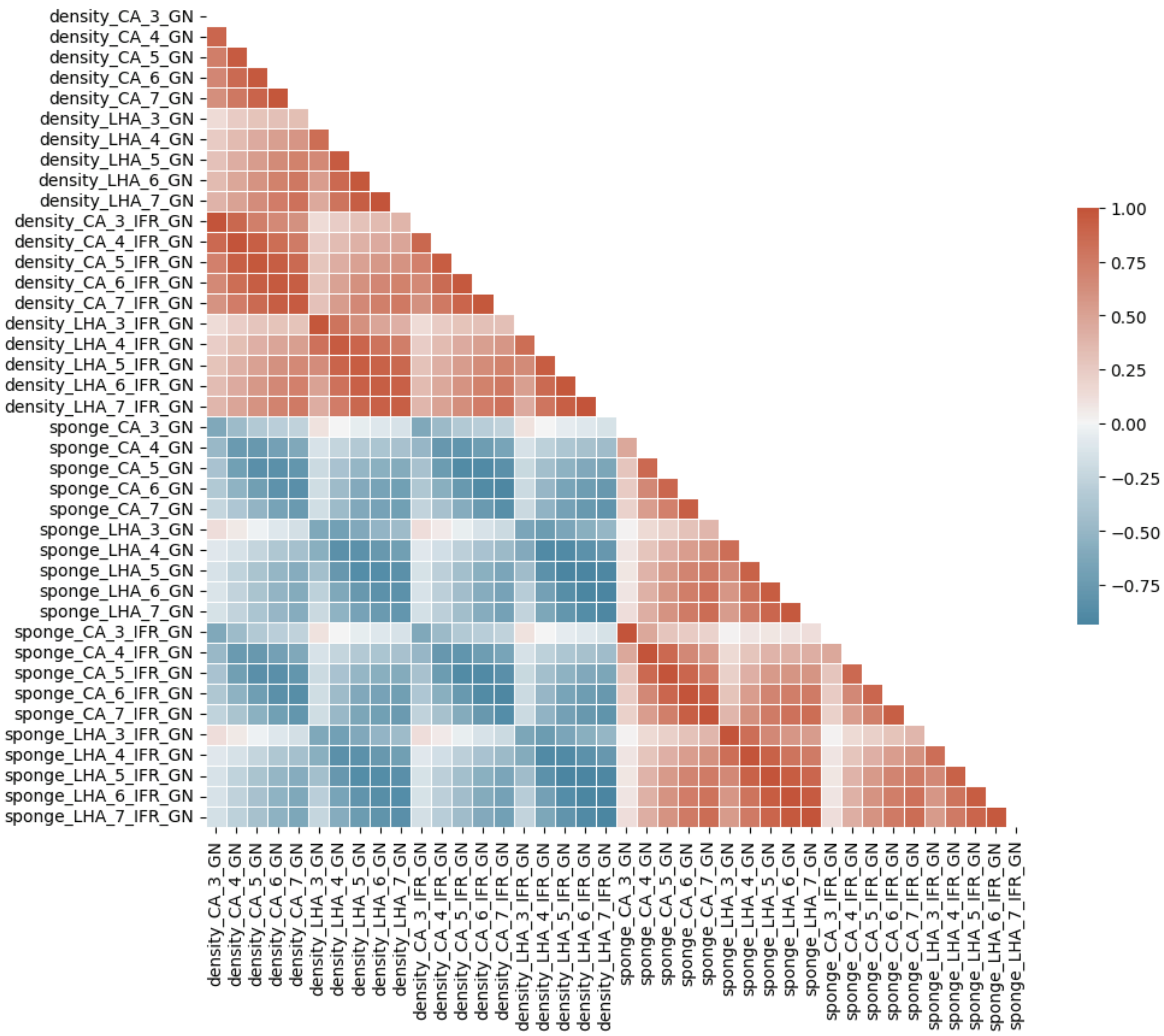

Weighted_Contact_Number

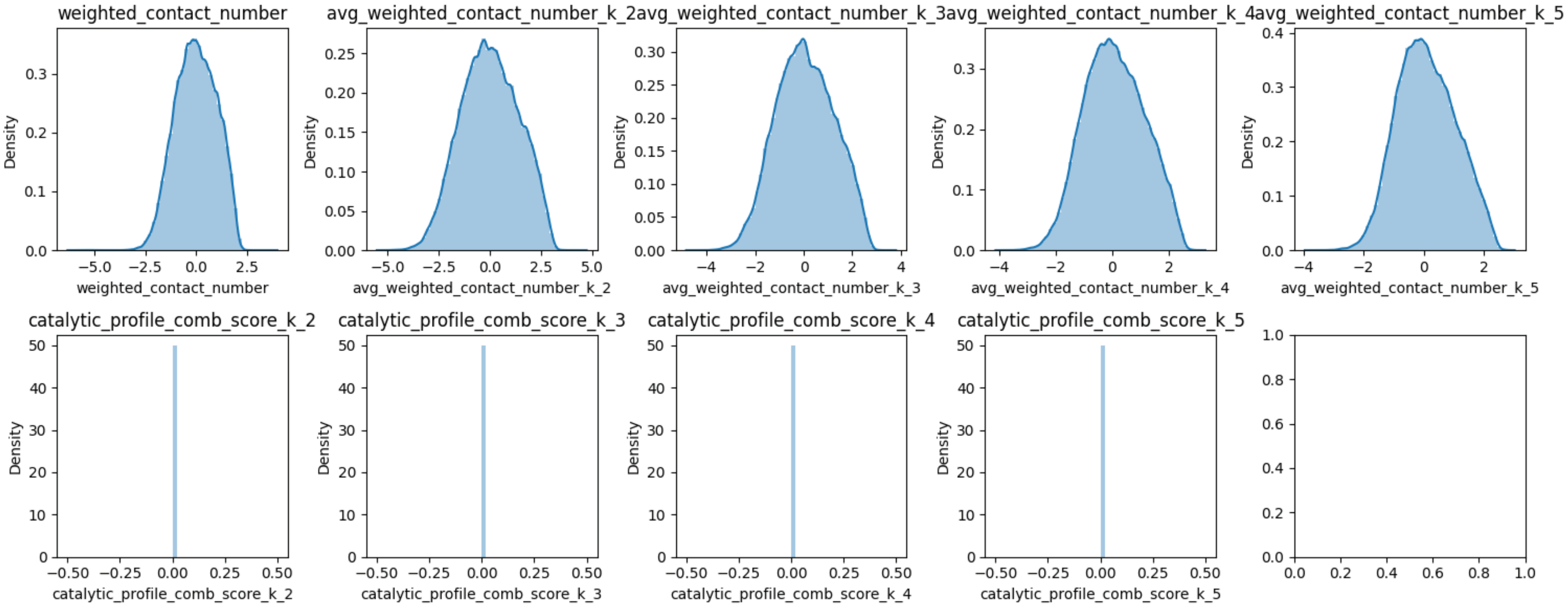

Distances

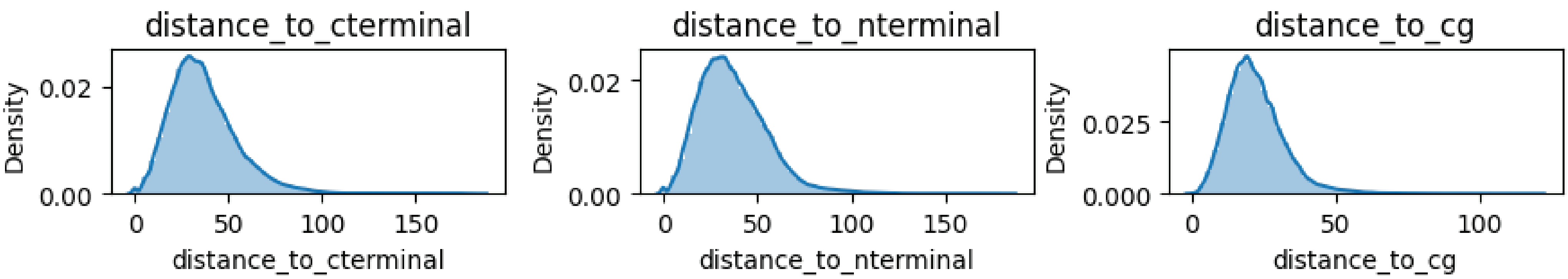
distance_to_cterminal
Density
0.02
0.00
0
50
100
150
distance_to_cterminal
distance_to_nterminal
Density
0.02
0.00
0
50
100
150
distance_to_nterminal
distance_to_cg
Density
0.025
0.000
0
50
100
distance_to_cg

Weighted_Contact_Number_WNA

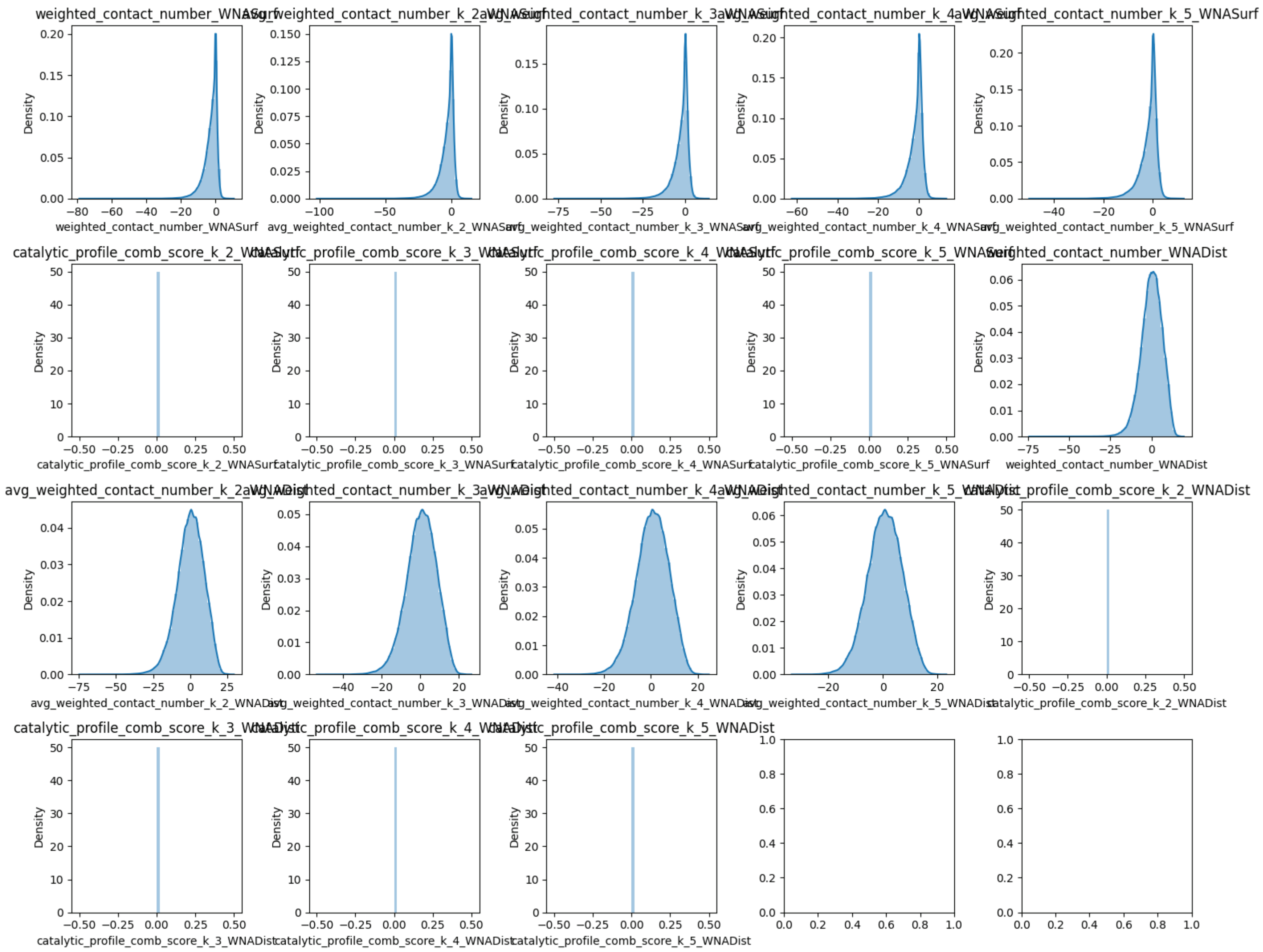

Hypothesis Verification

In statistical terms, our null hypothesis (H_0) is that the descriptor value distributions are the same for both classes, while our alternative hypothesis (H_1) is that the descriptor value distributions are not the same.

To test this hypothesis, we applied three statistical tests. The first test was the **Kolmogorov-Smirnov test (KS),** which is a non-parametric test used to compare the empirical distribution function of a sample to a reference probability distribution or to compare the empirical distribution functions of two samples. The KS statistic measures the maximum distance between the two empirical distribution functions.

The second test was the **Q-Q plot**, which is a graphical method for comparing two probability distributions by plotting their quantiles against each other. If the data have a normal distribution, then **Student's t-test** may be used.

Hypothesis Verification

1. Kolmogorov-Smirnov test (KS)

	Column	KS Statistic	p-value
0	density_CA_3	0.128242	1.110768e-136
1	density_CA_5	0.210097	0.000000e+00
2	density_CA_7	0.407871	0.000000e+00
3	density_LHA_4	0.212609	0.000000e+00
4	density_LHA_6	0.176447	3.308767e-259
5	density_CA_3_IFR	0.165315	2.171071e-227
6	density_CA_5_IFR	0.159295	4.550779e-211
7	density_CA_7_IFR	0.296390	0.000000e+00
8	density_LHA_4_IFR	0.179629	1.076353e-268
9	density_LHA_6_IFR	0.167219	1.111917e-232
10	sponge_CA_3	0.162763	2.146883e-220
11	sponge_CA_5	0.165524	5.737079e-228
12	sponge_CA_7	0.285503	0.000000e+00

The *p-value* is below this threshold of 0.05 for all descriptors used in the study, the null hypothesis (H_o) is rejected in favor of the alternative hypothesis (that the two groups (AFRs and FRs) come from different distributions)

1. Kolmogorov-Smirnov test (KS)

	Column	KS Statistic	p-value
13	sponge_LHA_4	0.137226	1.609762e-156
14	sponge_LHA_6	0.167940	1.059997e-234
15	sponge_CA_3_IFR	0.162763	2.146883e-220
16	sponge_CA_5_IFR	0.165648	2.598911e-228
17	sponge_CA_7_IFR	0.287837	0.000000e+00
18	sponge_LHA_4_IFR	0.137229	1.579567e-156
19	sponge_LHA_6_IFR	0.155896	3.964276e-202
20	hb_mm_energy_GN	0.157118	2.543096e-205
21	side_chain_average_angle_7	0.195795	1.362238e-319
22	neighbors_side_chain_angle_7	0.188539	3.479626e-296
23	background_rel_entropy	0.266057	0.000000e+00
24	avg_weighted_contact_numbe r_k_2	0.294597	0.000000e+00

Hypothesis Verification

2. Q-Q Plot

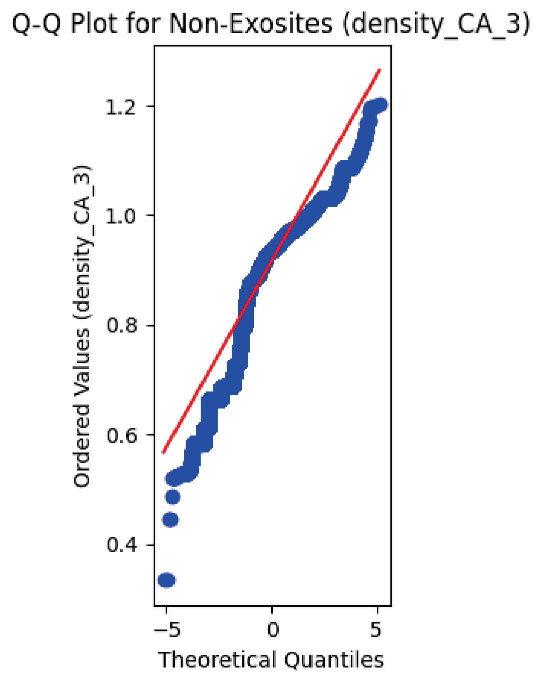

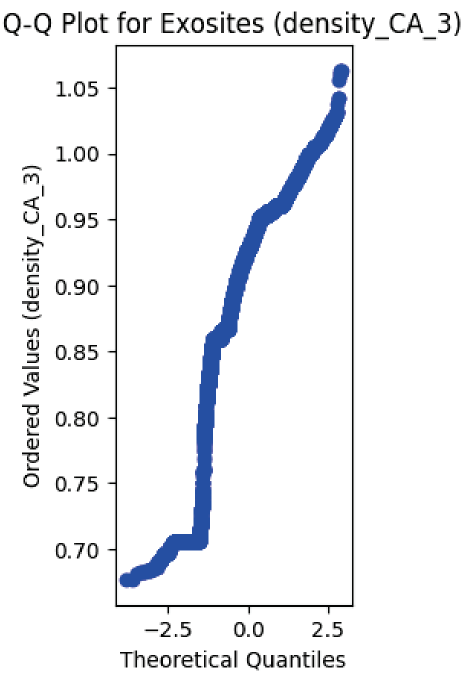

In this study, we used Q-Q plots to assess the normality of the distribution of several variables in our dataset. We split the data into two groups based on the target column and generated Q-Q plots for each group and each variable. The results showed that for most variables, the ordered values of the sample data closely followed the theoretical quantiles of the normal distribution, as indicated by the alignment of the blue points with the red line on the Q-Q plots. This suggests that the distribution of these variables is approximately normal in both groups. These findings have important implications for our analysis, as many statistical methods assume that the data follows a normal distribution.

Hypothesis Verification

2. Q-Q Plot

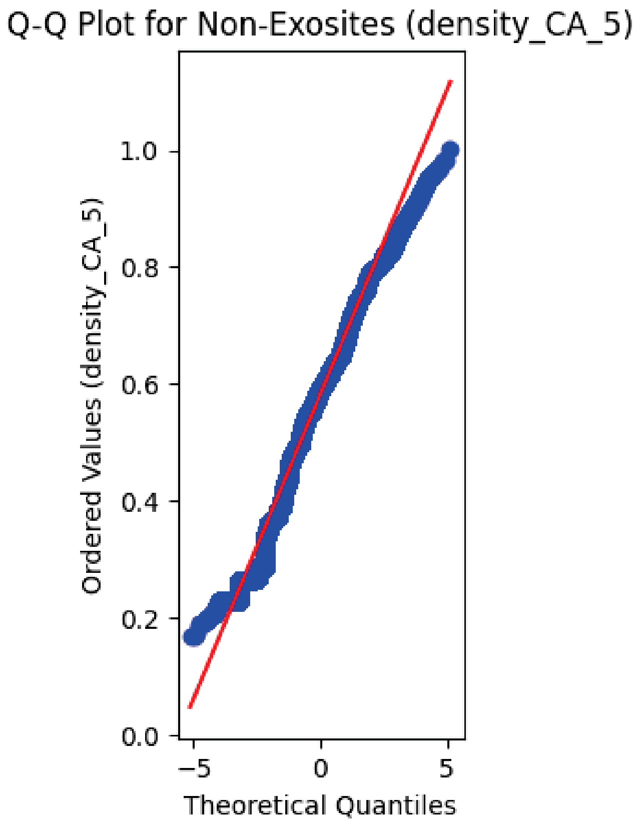

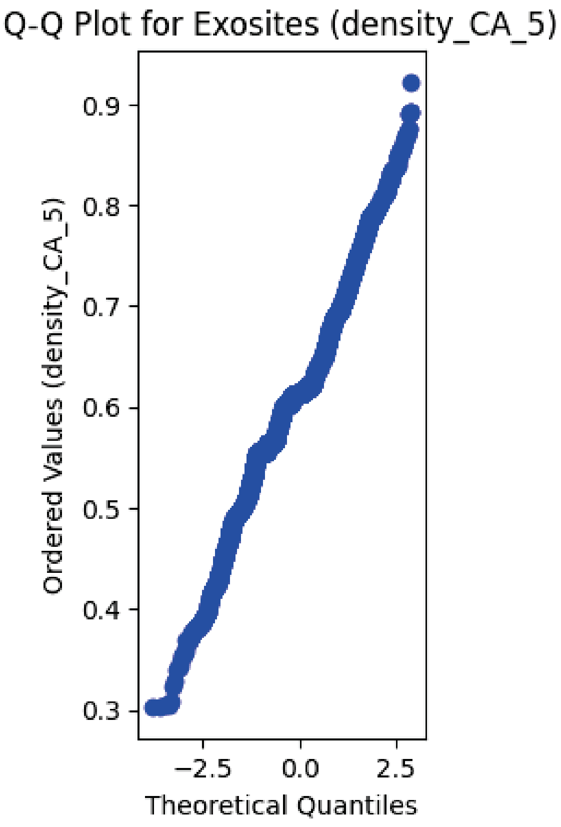

Hypothesis Verification

2. Q-Q Plot

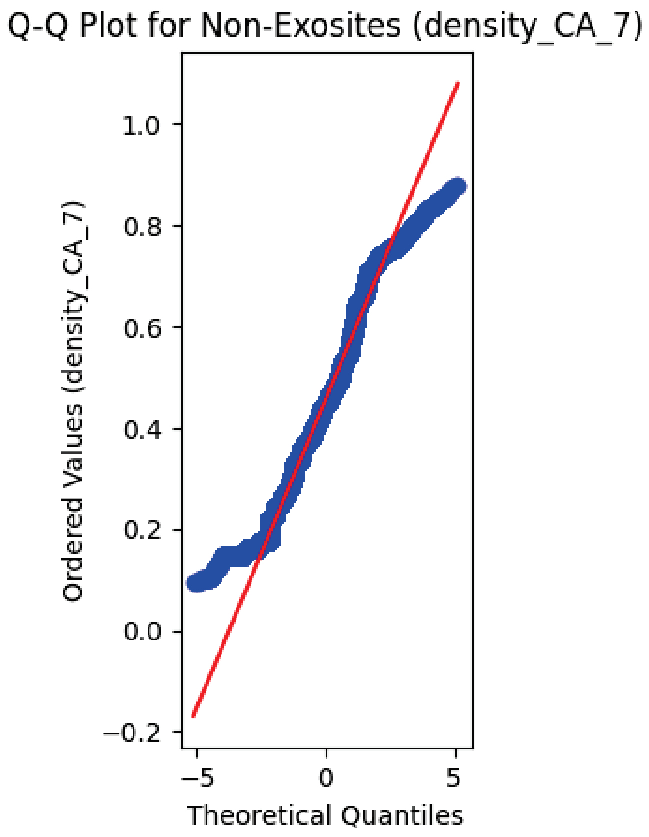

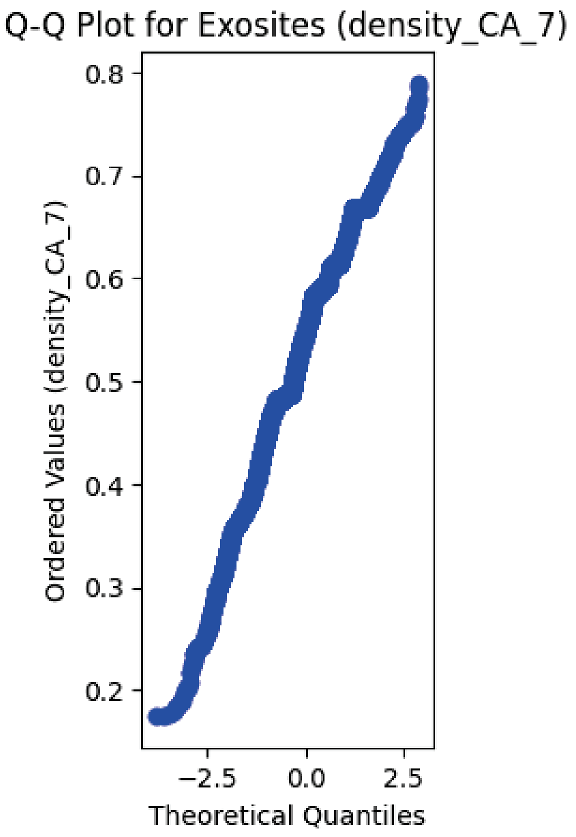

Hypothesis Verification

2. Q-Q Plot

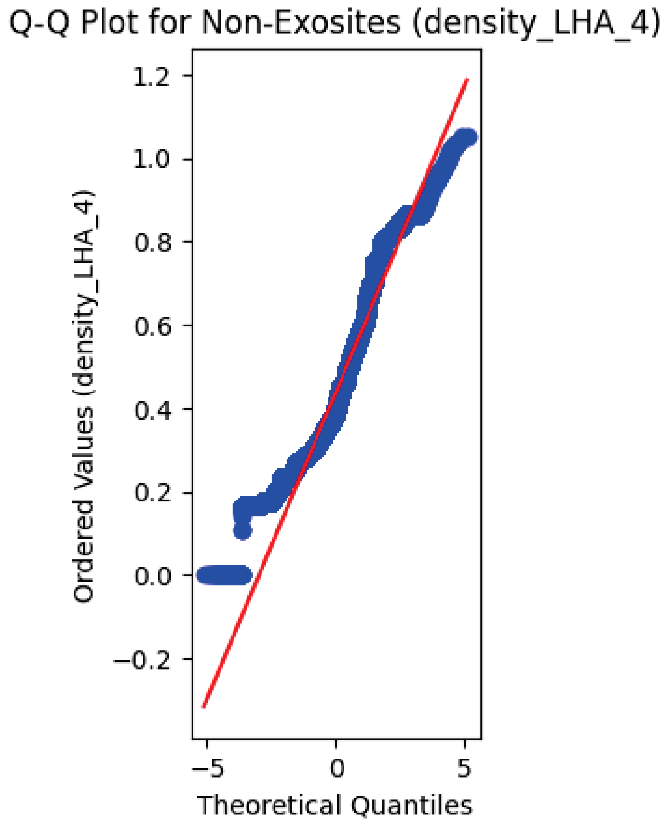

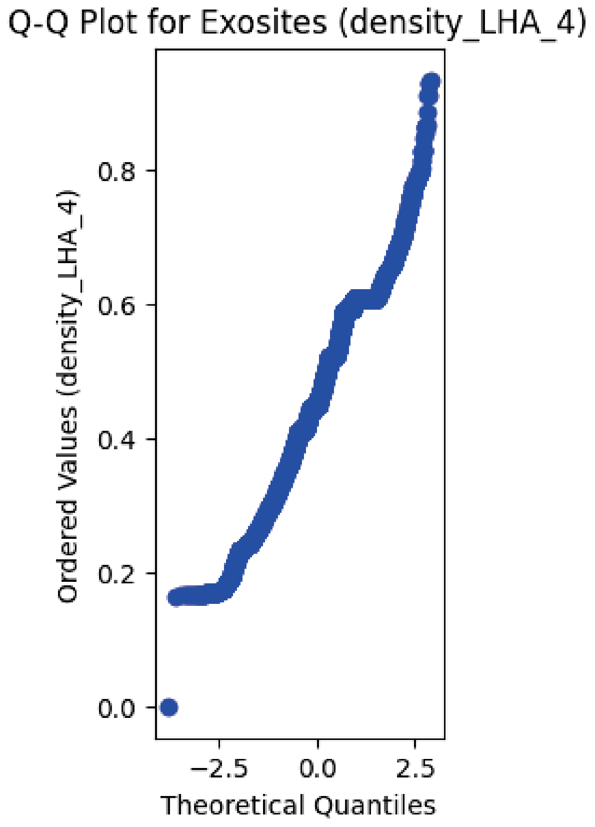

Hypothesis Verification

2. Q-Q Plot

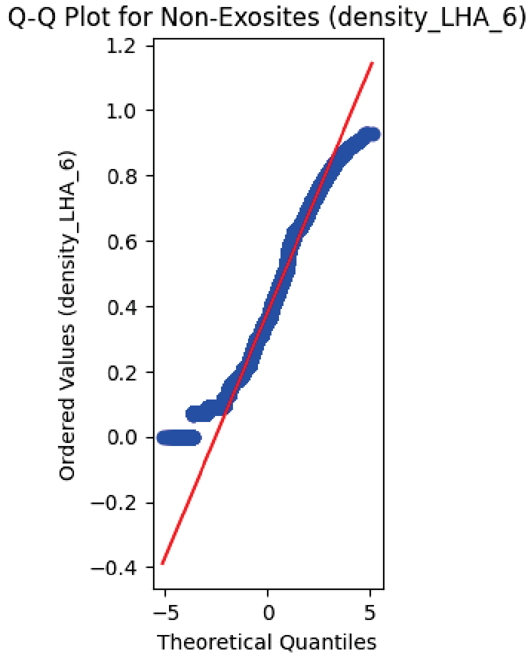

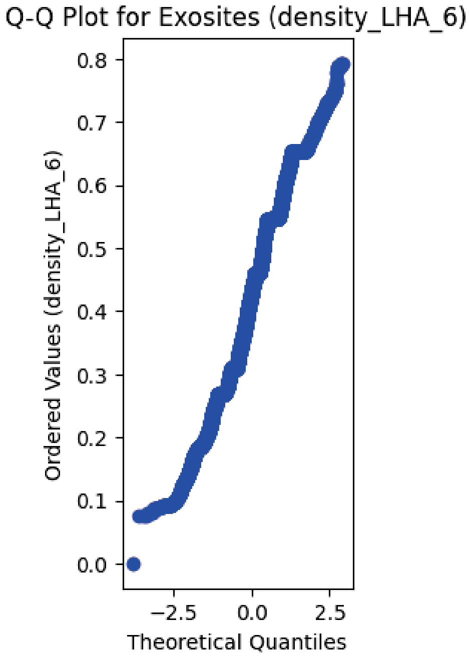

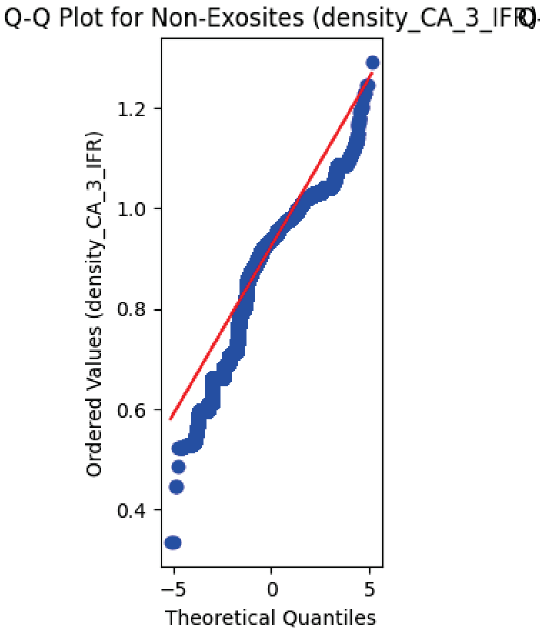

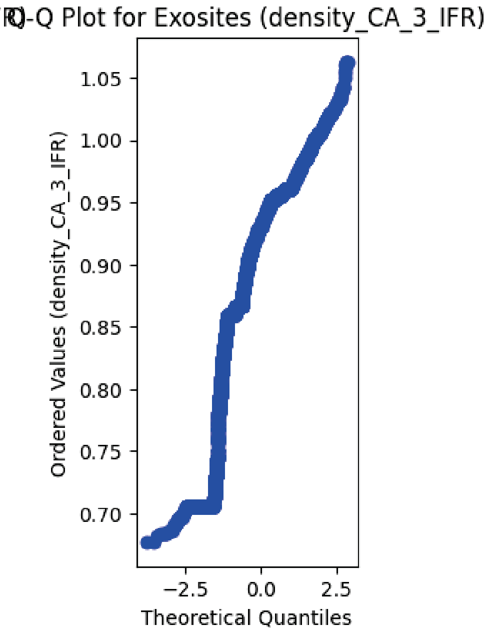

Hypothesis Verification

2. Q-Q Plot

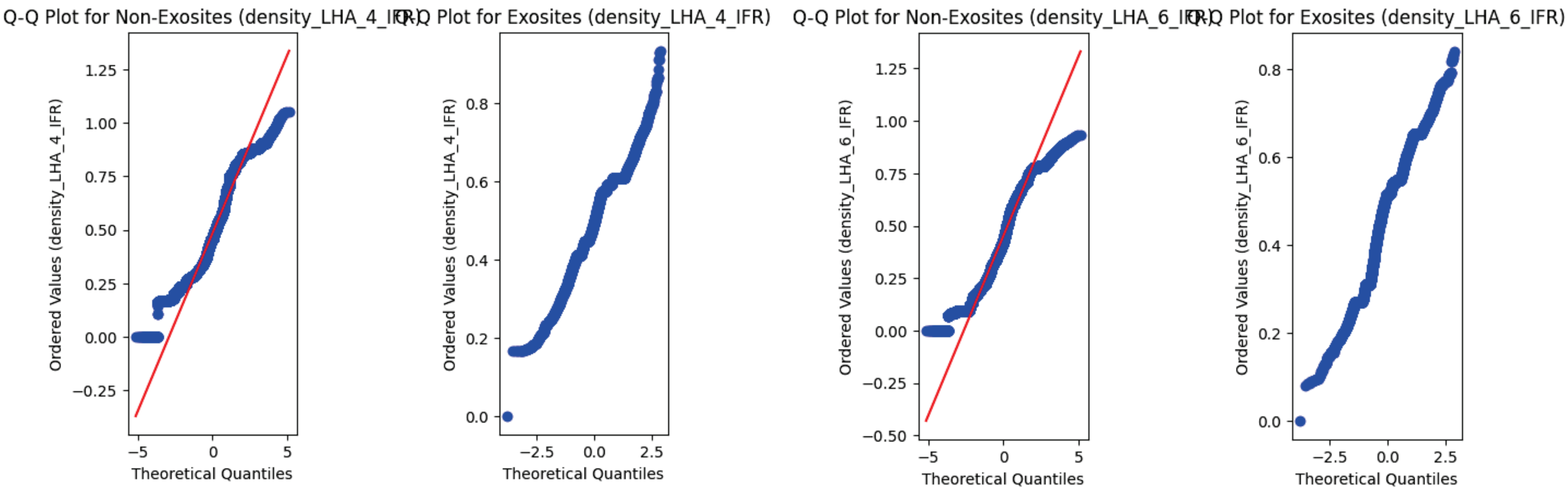

Hypothesis Verification

2. Q-Q Plot

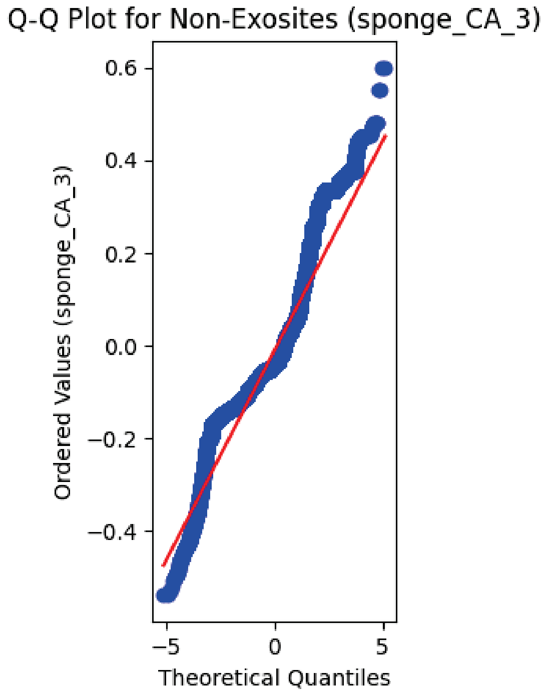

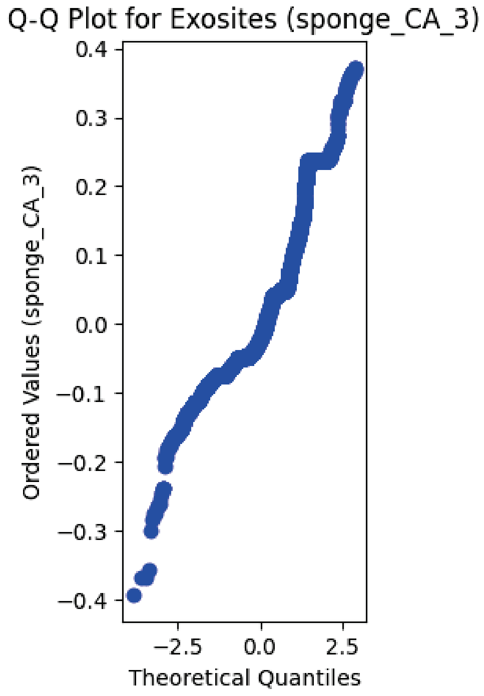

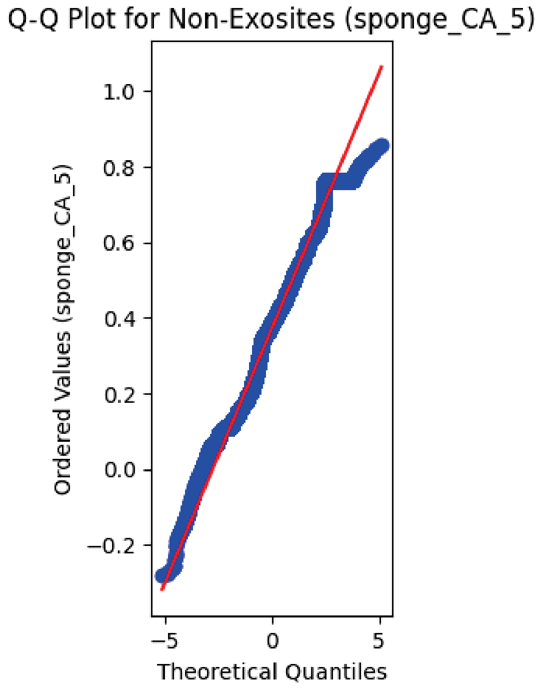

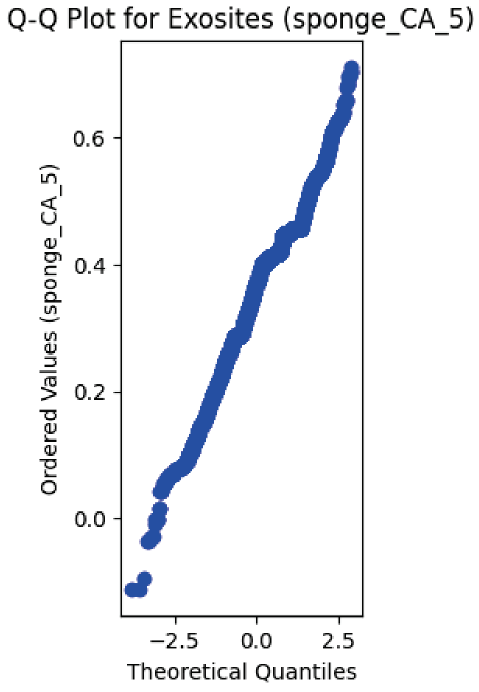

Hypothesis Verification

2. Q-Q Plot

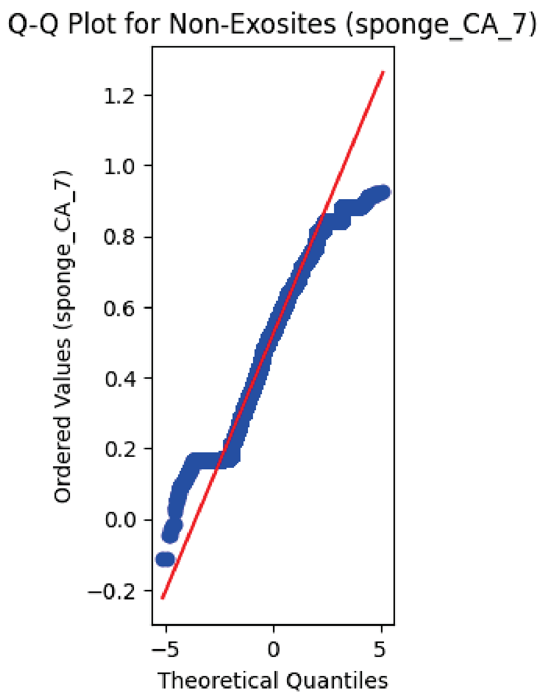

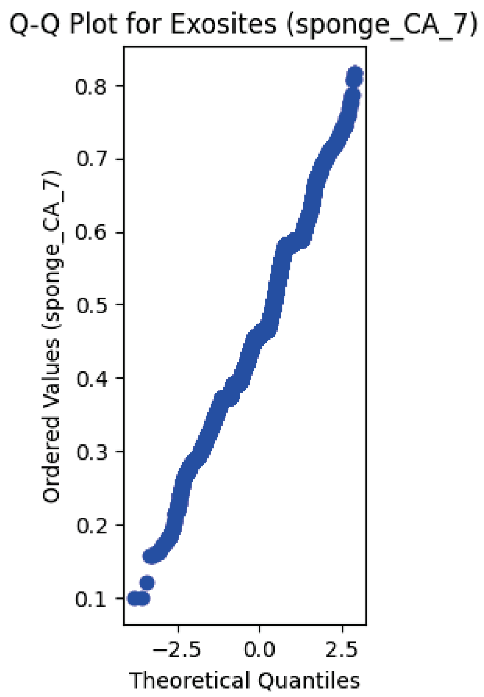

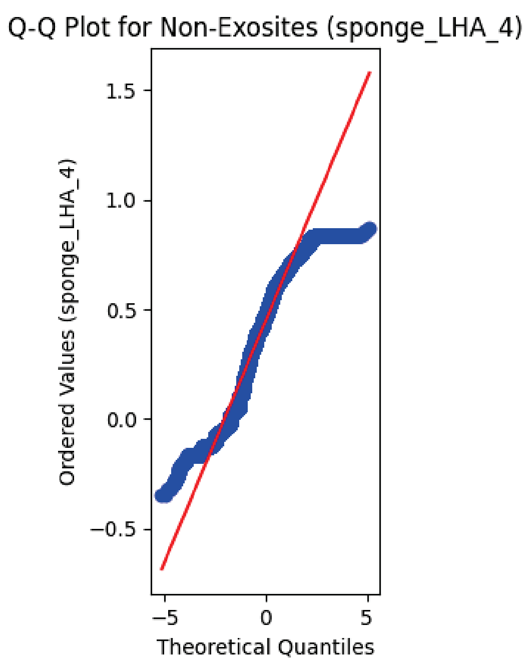

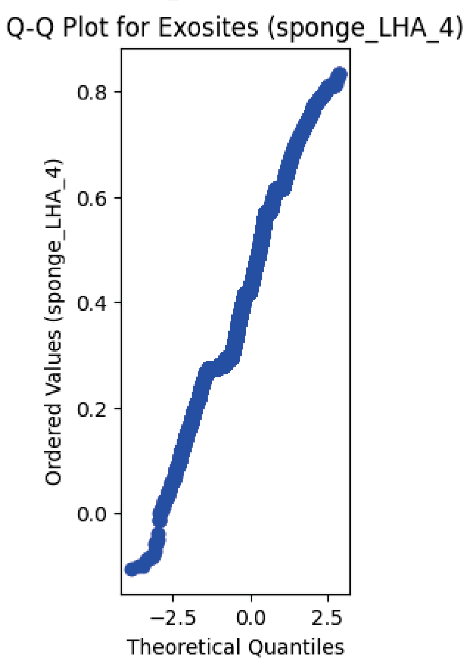

Hypothesis Verification

2. Q-Q Plot

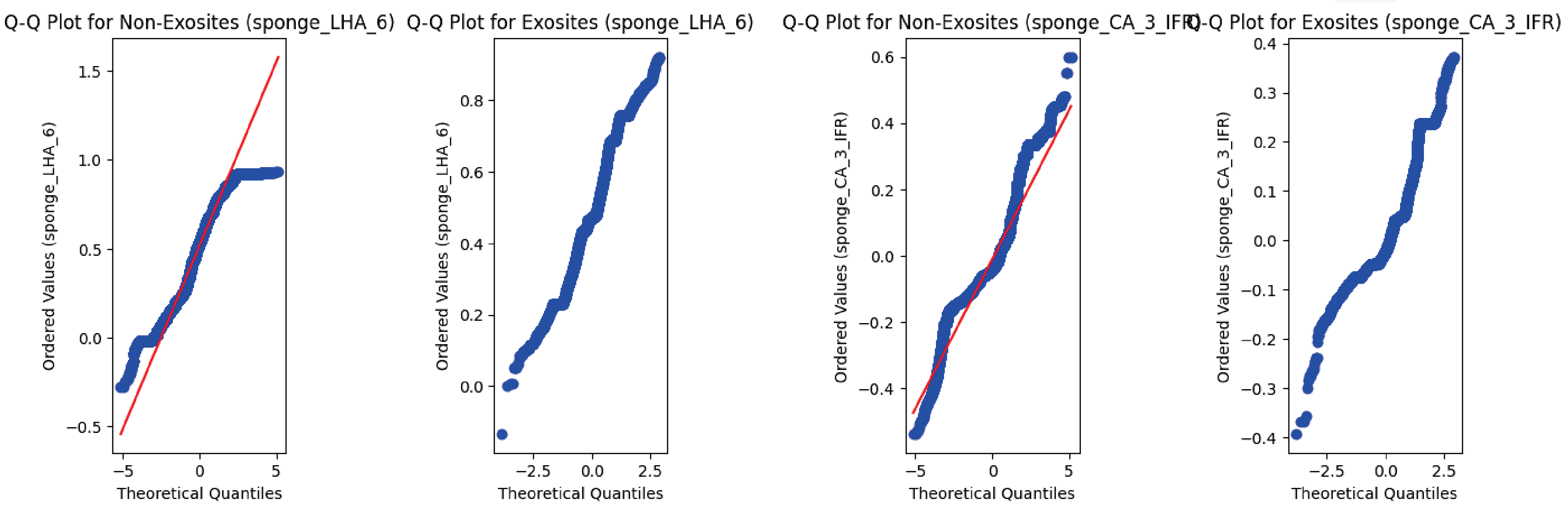

10. II) Hypothesis Verification

2. Q-Q Plot

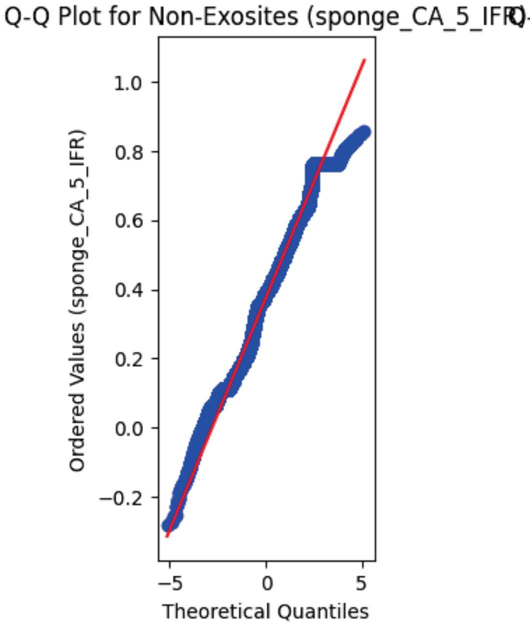

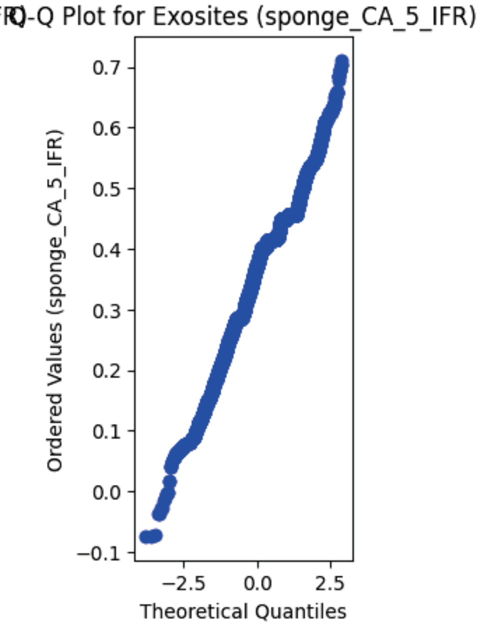

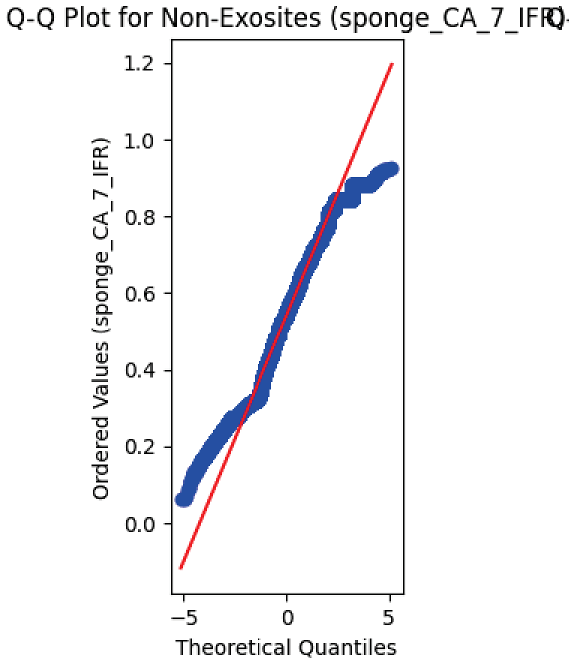

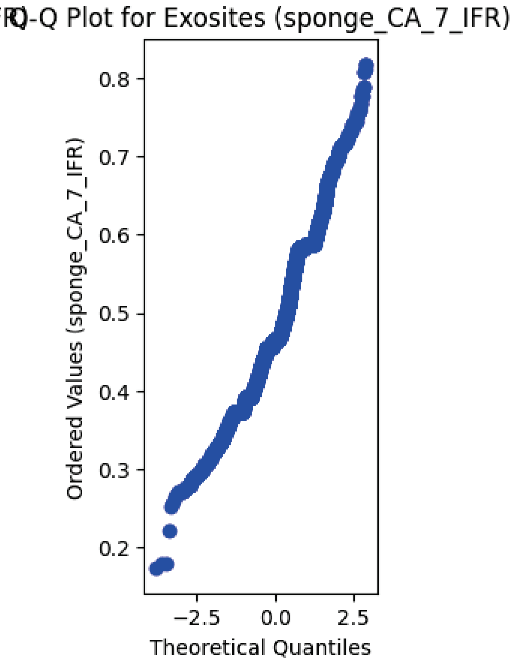

10. II) Hypothesis Verification

2. Q-Q Plot

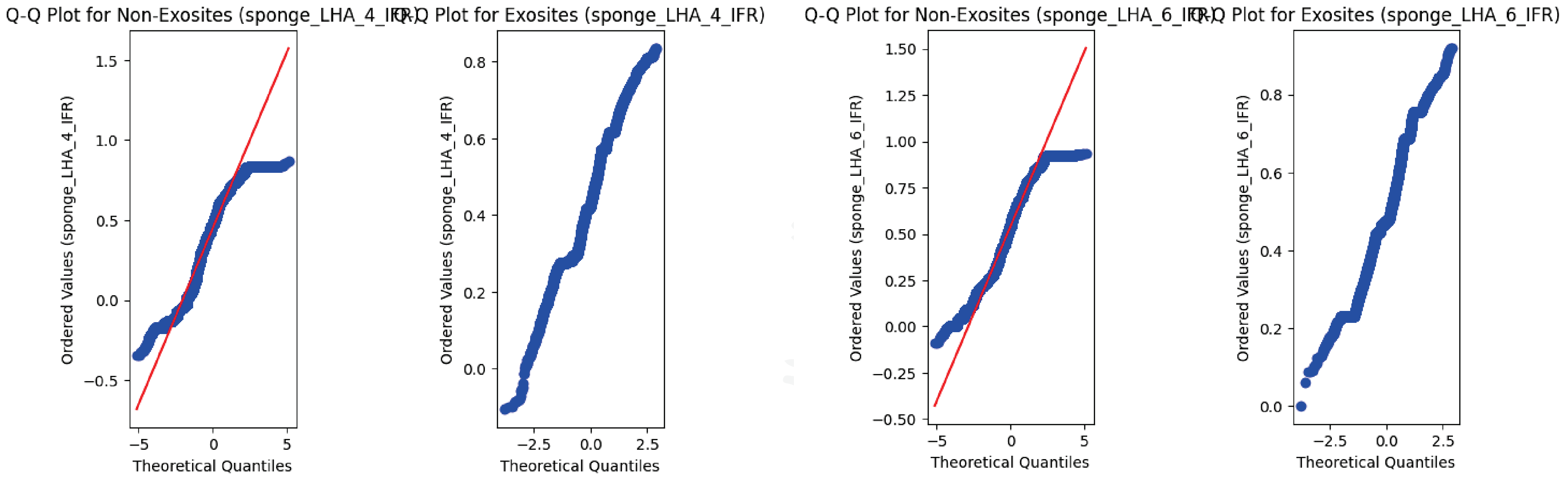

Hypothesis Verification

2. Q-Q Plot

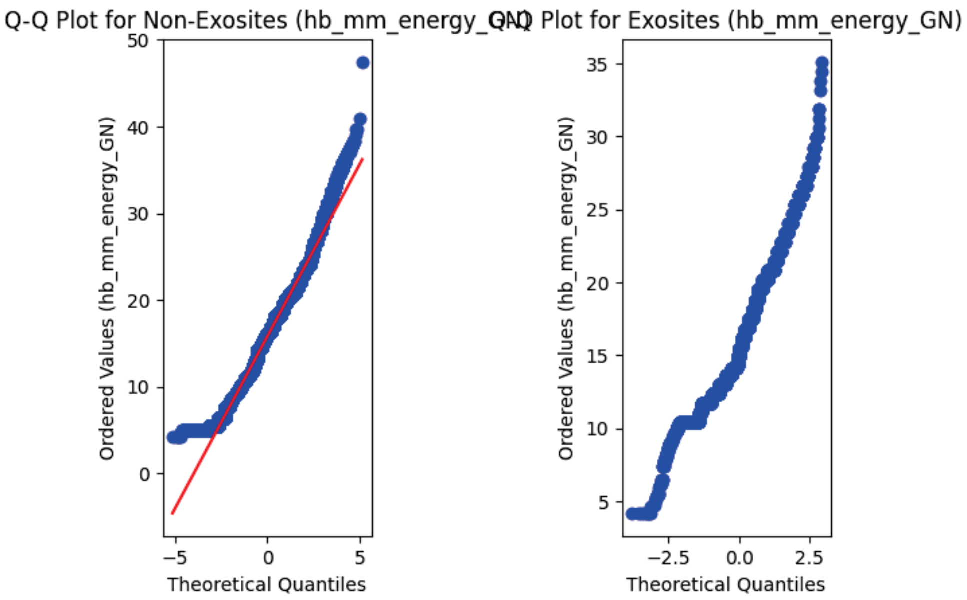

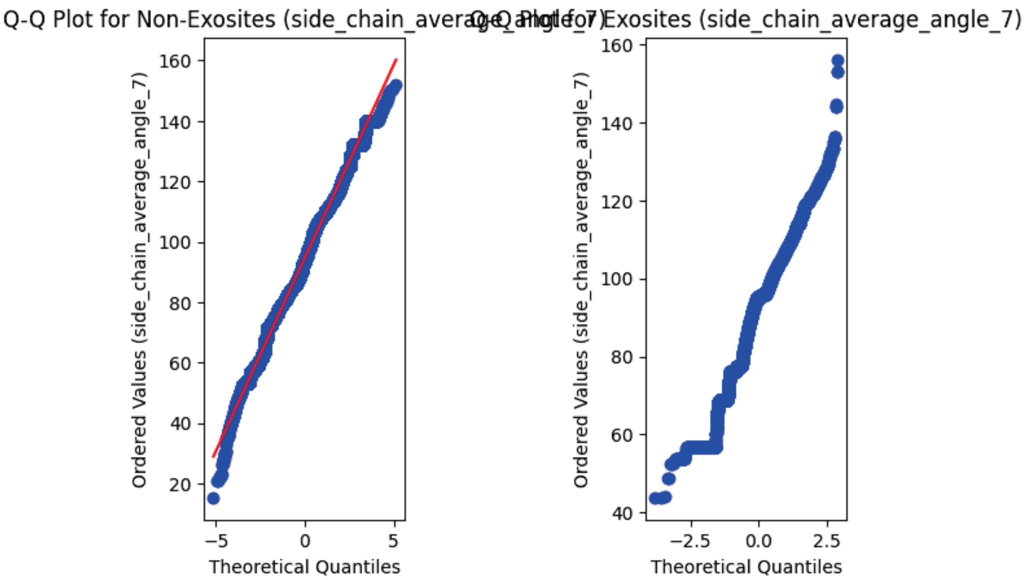

Hypothesis Verification

2. Q-Q Plot

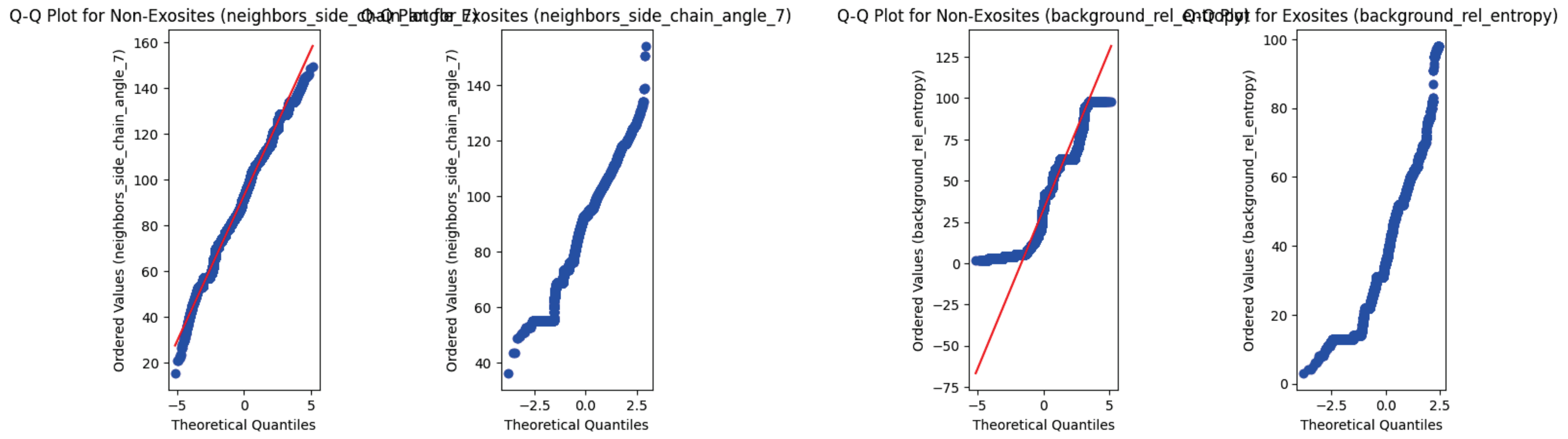

Hypothesis Verification

2. Q-Q Plot

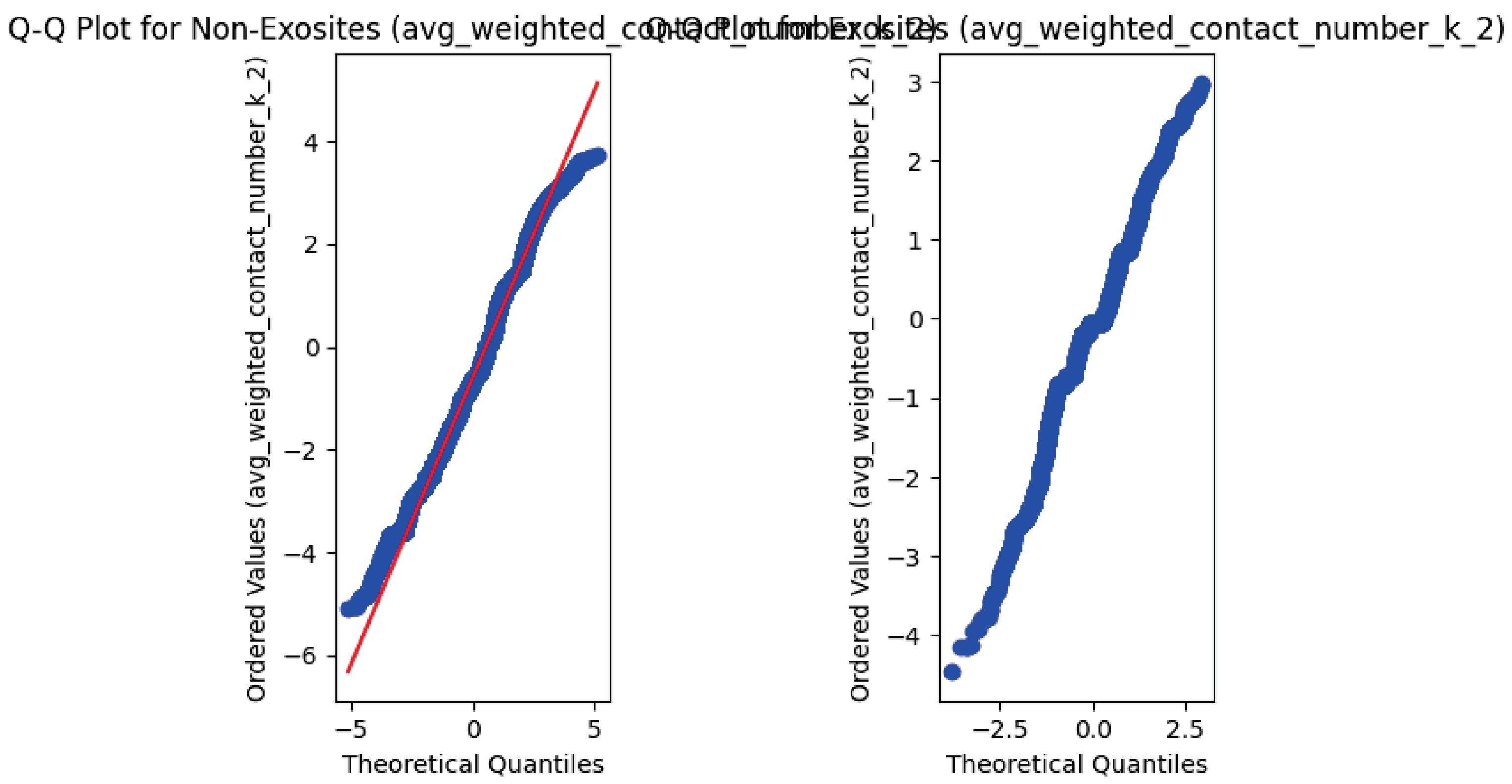

Hypothesis Verification

3. T-Test

Column: density_CA_3 t-statistic: 13.4425 p-value: 0.0000
Column: density_CA_5 t-statistic: -31.8862 p-value: 0.0000
Column: density_CA_7 t-statistic: -65.1099 p-value: 0.0000
Column: density_LHA_4 t-statistic: -15.1673 p-value: 0.0000
Column: density_LHA_6 t-statistic: -28.5012 p-value: 0.0000
Column: density_CA_3_IFR t-statistic: 20.9910 p-value: 0.0000
Column: density_CA_5_IFR t-statistic: -10.2439 p-value: 0.0000
Column: density_CA_7_IFR t-statistic: -39.8751 p-value: 0.0000
Column: density_LHA_4_IFR t-statistic: -4.0531 p-value: 0.0001
Column: density_LHA_6_IFR t-statistic: -13.0732 p-value: 0.0000
Column: sponge_CA_3 t-statistic: -21.5210 p-value: 0.0000
Column: sponge_CA_5 t-statistic: 16.1055 p-value: 0.0000
Column: sponge_CA_7 t-statistic: 32.2656 p-value: 0.0000
Column: sponge_LHA_4 t-statistic: -0.4440 p-value: 0.6571
Column: sponge_LHA_6 t-statistic: 14.6871 p-value: 0.0000
Column: sponge_CA_3_IFR t-statistic: -21.5210 p-value: 0.0000

We performed a t-test to compare the AFR and FRs groups using different descriptors from the STING database. The results showed significant differences between the two groups for several descriptors, including density_CA_3 (t(13.4425), $p < 0.0001$), density_CA_5 (t(-31.8862), $p < 0.0001$), and density_CA_7 (t(-65.1099), $p < 0.0001$). There were also significant differences for density_LHA_4 (t(-15.1673), $p < 0.0001$) and density_LHA_6 (t(-28.5012), $p < 0.0001$). These results suggest that there are significant differences in the distribution of these descriptors between the Exosite and Non-Exosite groups.

However, there was no significant difference for sponge_LHA_4 (t(-0.4440), $p = 0.6571$), s and sponge_LHA_4_IFR (t(-0.1621), $p = 0.8712$) in red.

All other descriptors shows significant differences.

Hypothesis Verification

3. T-Test

Column: sponge_CA_5_IFR t-statistic: 16.3968 p-value: 0.0000
Column: sponge_CA_7_IFR t-statistic: 44.6354 p-value: 0.0000
Column: sponge_LHA_4_IFR t-statistic: -0.1621 p-value: 0.8712
Column: sponge_LHA_6_IFR t-statistic: 19.2281 p-value: 0.0000
Column: hb_mm_energy_GN t-statistic: -3.1193 p-value: 0.0018
Column: side_chain_average_angle_7 t-statistic: 23.2658 p-value: 0.0000
Column: neighbors_side_chain_angle_7 t-statistic: 21.9113 p-value: 0.0000
Column: background_rel_entropy t-statistic: -26.3763 p-value: 0.0000
Column: avg_weighted_contact_number_k_2 t-statistic: -41.9366 p-value: 0.0000
