# Supplementary material for: Protein allosteric site identification using machine learning and per amino acid residue reported internal protein nanoenvironment descriptors
Source: Comput Struct Biotechnol J. 2024 Oct 23;23:3907–19. doi: 10.1016/j.csbj.2024.10.036 (PMC11570862; doi:10.1016/j.csbj.2024.10.036)
Supplement: Supplementary file 7 — Supplementary material [file mmc7.docx]

## **Supplementary**

## 1.1 **XGBoost Algorithm**

XGBoost, an acronym for eXtreme Gradient Boosting, leverages the gradient boosting framework to sequentially correct errors from previous models by adding new models. Its objective function, optimized during training, integrates a loss function *l* and a regularization term Ω, enhancing model performance and complexity control:

$$\text{Obj}\left( \theta\right)=\sum_{i=1}^{n} l\left( y_{i},\hat{y}_{i}^{\left( t \right)} \right)+\sum_{k=1}^{t} \Omega\left( f_{k} \right),$$

where $l$ represents the loss function measuring the deviation between predicted $\hat{y}_{i}^{\left( t \right)}$ and actual $y_{i}$ outputs, $\Omega$ denotes regularization, and $f_{k}$ is the $k$-th tree in the model ensemble.

### **1.2. LightGBM Algorithm**

LightGBM stands out for its efficiency and scalability, primarily due to its histogram-based learning algorithm, which significantly reduces memory requirements and accelerates training times. Its core principle is captured by:

$$\hat{y}_{i}^{\left( t \right)}=\hat{y}_{i}^{\left( t-1 \right)}+\eta f_{t}\left( x_{i} \right),$$

where $\hat{y}_{i}^{\left( t \right)}$ is the prediction for the $i$-th instance at iteration $t$, with $\eta$ representing the learning rate and $f_{t}$ the $t$-th tree’s contribution. LightGBM’s optimization targets a similar objective function as XGBoost but introduces innovative features like Gradient-based One-Side Sampling (GOSS) and Exclusive Feature Bundling (EFB) to boost efficiency and performance.

2. **ROC Analysis Across Amino Acids**

We conducted a performance assessment of binary classifiers developed for each of the 20 canonical amino acids utilizing the ROC-AUC metric as the principal measure of discriminative efficiency. Scores approaching unity indicate superior class differentiation capabilities (AFR versus FRs).

**Figure S1:** Receiver Operating Characteristic (ROC) curves for the 20 standard amino acids, demonstrating the model’s ability to distinguish AFR from FRs. Each curve represents an amino acid, with the area under the curve (AUC) indicating the predictive performance: higher AUC values suggest better discriminative ability. The curves are ordered by their F1 scores, showcasing a range of model efficacies across different biochemical contexts.

The classifiers for Methionine (MET) and Tryptophan (TRP) exhibited outstanding performance, with ROC-AUC values of 0.95 and 0.92, respectively, demonstrating remarkable proficiency in distinguishing between affirmative and negative cases for these amino acids. These results reflect the classifiers’ proficiency in optimizing the trade-off between recall (sensitivity) and precision (positive predictive value). Further scrutiny into the F1 Score components revealed variability in recall among the classifiers, with the Tryptophan classifier attaining the highest recall rate of 0.6800, signifying its exceptional ability to correctly identify true positive cases.

Additionally, a subset of classifiers, particularly those for Tryptophan, Methionine, and Aspartic Acid (ASP), achieved precision scores above 0.8, indicating a notable minimization of false positives.

# 3. Experimental-Curated AFRs from ASD2023 and ASBench Datasets

In ASD 2023[19], there are 3102 curated proteins with experimentally determined allosteric site residues listed. The database contains 18 columns, including *modulator_serial, modulator_alias, modulator_chain, modulator_class, modulator_feature, modulator_name, and modulator_resii,* which provide information about the modulators interacting with the protein. Of particular importance are *allosteric_pdb* (to work with proteins with annotated allosteric sites), *ref_title* (to identify different allosteric PDBs of the same protein), and *site overlap*, which indicates whether the allosteric PDBs overlap with the protein’s active site. We identified 1622 allosteric PDBs within the ASD lacking allosteric site residues, resulting in 1480 valid allosteric PDBs with allosteric site residues out of the initial 3102. After removing duplicates, we obtained a dataset of 763 unique proteins. In this context,” unique” means that we do not consider proteins that appear more than once with identical allosteric sites. Further filtering using the RCSB API to identify proteins with PDB resolution less than 3.0 Å and no overlap with active sites (as defined in the ASD database) reduced the dataset to 589 proteins.

4. **Performance Metrics Across Different Models**

In evaluating the performance of CatBoost, XGBoost, and LightGBM, we focused on F1 Score and MCC, which are particularly suited for handling imbalanced datasets. These metrics provided insights into how well each model balanced precision and recall in predicting AFRs. CatBoost consistently outperformed XGBoost and LightGBM across most residues. For example, CYS saw a notable improvement in CatBoost’s predictions, with an F1 Score increasing to 0.67 and an MCC of 0.67, compared to 0.40 and 0.49 in XGBoost, and 0.38 and 0.45 in LightGBM. Similar trends were observed for residues like TRP, MET, and ASP, where CatBoost demonstrated superior performance over the other models. While all three models performed reasonably well in predicting AFRs, CatBoost’s ability to extract important features from the internal protein nanoenvironment, allowed it to deliver more accurate predictions. Table S1 provides a detailed breakdown of performance metrics for each residue across the three models.

**Table S1**: Performance Metrics for Residues Across Different Models on the validation set

| **Residue** | **Model** | **F1 Score** | **MCC** | **Recall** | **Precision** |
| --- | --- | --- | --- | --- | --- |
| CYS | XGBoost | 0.40 | 0.49 | 0.38 | 0.42 |
|  | LightGBM | 0.38 | 0.45 | 0.36 | 0.41 |
|  | CatBoost | 0.67 | 0.67 | 0.55 | 0.77 |
| TRP | XGBoost | 0.78 | 0.77 | 0.72 | 0.85 |
|  | LightGBM | 0.76 | 0.75 | 0.70 | 0.83 |
|  | CatBoost | 0.81 | 0.81 | 0.74 | 0.88 |
| MET | XGBoost | 0.72 | 0.70 | 0.68 | 0.75 |
|  | LightGBM | 0.71 | 0.69 | 0.66 | 0.74 |
|  | CatBoost | 0.74 | 0.73 | 0.70 | 0.78 |
| ASP | XGBoost | 0.65 | 0.67 | 0.60 | 0.70 |
|  | LightGBM | 0.63 | 0.65 | 0.58 | 0.68 |
|  | CatBoost | 0.72 | 0.72 | 0.65 | 0.80 |
| HIS | XGBoost | 0.66 | 0.64 | 0.60 | 0.72 |
|  | LightGBM | 0.65 | 0.63 | 0.58 | 0.71 |
|  | CatBoost | 0.71 | 0.69 | 0.64 | 0.76 |
| THR | XGBoost | 0.68 | 0.67 | 0.62 | 0.75 |
|  | LightGBM | 0.66 | 0.65 | 0.60 | 0.73 |
|  | CatBoost | 0.71 | 0.71 | 0.63 | 0.80 |
| ARG | XGBoost | 0.60 | 0.62 | 0.54 | 0.67 |
|  | LightGBM | 0.58 | 0.60 | 0.52 | 0.65 |
|  | CatBoost | 0.69 | 0.67 | 0.63 | 0.75 |
| GLY | XGBoost | 0.62 | 0.60 | 0.56 | 0.69 |
|  | LightGBM | 0.61 | 0.59 | 0.55 | 0.67 |
|  | CatBoost | 0.67 | 0.66 | 0.59 | 0.78 |
| PHE | XGBoost | 0.61 | 0.60 | 0.55 | 0.68 |
|  | LightGBM | 0.60 | 0.59 | 0.54 | 0.67 |
|  | CatBoost | 0.67 | 0.66 | 0.59 | 0.77 |
| ASN | XGBoost | 0.62 | 0.61 | 0.56 | 0.69 |
|  | LightGBM | 0.60 | 0.59 | 0.54 | 0.67 |
|  | CatBoost | 0.67 | 0.66 | 0.57 | 0.80 |
| LYS | XGBoost | 0.61 | 0.60 | 0.55 | 0.68 |
|  | LightGBM | 0.60 | 0.59 | 0.54 | 0.67 |
|  | CatBoost | 0.65 | 0.64 | 0.58 | 0.75 |
| SER | XGBoost | 0.60 | 0.62 | 0.54 | 0.67 |
|  | LightGBM | 0.58 | 0.60 | 0.52 | 0.65 |
|  | CatBoost | 0.64 | 0.66 | 0.50 | 0.91 |
| TYR | XGBoost | 0.57 | 0.58 | 0.50 | 0.65 |
|  | LightGBM | 0.56 | 0.57 | 0.49 | 0.64 |
|  | CatBoost | 0.62 | 0.61 | 0.52 | 0.77 |
| PRO | XGBoost | 0.58 | 0.57 | 0.52 | 0.66 |
|  | LightGBM | 0.57 | 0.56 | 0.51 | 0.65 |
|  | CatBoost | 0.61 | 0.60 | 0.56 | 0.67 |
| VAL | XGBoost | 0.57 | 0.56 | 0.50 | 0.64 |
|  | LightGBM | 0.56 | 0.55 | 0.49 | 0.63 |
|  | CatBoost | 0.61 | 0.62 | 0.51 | 0.77 |
| ALA | XGBoost | 0.56 | 0.57 | 0.49 | 0.63 |
|  | LightGBM | 0.55 | 0.56 | 0.48 | 0.62 |
|  | CatBoost | 0.60 | 0.62 | 0.45 | 0.88 |
| GLU | XGBoost | 0.55 | 0.56 | 0.48 | 0.62 |
|  | LightGBM | 0.54 | 0.55 | 0.47 | 0.61 |
|  | CatBoost | 0.59 | 0.59 | 0.47 | 0.77 |
| ILE | XGBoost | 0.53 | 0.54 | 0.46 | 0.60 |
|  | LightGBM | 0.52 | 0.53 | 0.45 | 0.59 |
|  | CatBoost | 0.58 | 0.57 | 0.48 | 0.71 |
| LEU | XGBoost | 0.52 | 0.53 | 0.44 | 0.60 |
|  | LightGBM | 0.51 | 0.52 | 0.43 | 0.58 |
|  | CatBoost | 0.54 | 0.54 | 0.43 | 0.71 |
| GLN | XGBoost | 0.47 | 0.48 | 0.41 | 0.54 |
|  | LightGBM | 0.46 | 0.47 | 0.40 | 0.53 |
|  | CatBoost | 0.50 | 0.55 | 0.35 | 0.90 |

**Figure S2:** Performance Metrics of Predictive Models on AFR Identification: This figure juxtaposes the F1 scores and MCC for CatBoost (blue), LightGBM (green), and XGBoost (red) models, showcasing the predictive performance across a spectrum of amino acid residues. The dual metric comparison shows the models’ effectiveness in discerning AFRs, with good F1-Scores and MCC, highlighting the striking balance between precision, recall, and overall accuracy in the prediction.

**Table S2**: Complete DCC Values for All Proteins and Chains in the Test Set Across Different Models

| PDB_Code | Chain | STINGAllo | PASSER Ensemble | PASSER AutoML | PASSER Rank |
| --- | --- | --- | --- | --- | --- |
| 1XXA | B | 0.00 | 10.91 | 10.91 | 10.91 |
| 2ZFZ | F | 0.00 | 13.70 | 13.70 | 13.70 |
| 1XXA | F | 0.00 | 7.82 | 7.82 | 7.82 |
| 3ETG | A | 0.00 | 21.20 | 12.95 | 12.95 |
| 3ETG | D | 0.00 | N/A | 12.93 | 12.93 |
| 2V4Y | F | 0.00 | 16.49 | 16.49 | 16.19 |
| 2LDB | D | 0.00 | 3.41 | 3.41 | 3.41 |
| 2ZFZ | B | 0.00 | 9.14 | 9.14 | 13.59 |
| 3ETE | C | 0.37 | 3.82 | 3.82 | 3.82 |
| 1EFA | B | 0.41 | 3.27 | 3.27 | 3.27 |
| 4A2U | F | 0.43 | 1.17 | 1.17 | 1.17 |
| 2I7N | B | 0.60 | 15.83 | 15.83 | 15.83 |
| 3GCD | D | 0.60 | 4.94 | 4.94 | 13.47 |
| 3PTZ | D | 0.61 | 2.38 | 2.38 | 2.38 |
| 2W4I | E | 0.63 | 2.28 | 2.28 | 2.28 |
| 3NWY | E | 0.67 | 20.37 | 4.64 | 7.14 |
| 3R6S | C | 0.92 | 7.12 | 7.12 | 7.12 |
| 2Q5O | A | 0.92 | 0.00 | 21.09 | 0.00 |
| 3PG9 | B | 0.94 | 45.26 | 45.26 | 22.36 |
| 1XTU | D | 0.96 | N/A | 18.39 | 18.39 |
| 1XTU | C | 0.96 | 18.42 | 18.42 | 18.42 |
| 4MBS | A | 1.01 | 4.92 | 4.92 | 32.50 |
| 2I7P | C | 1.08 | 13.41 | 13.41 | 13.41 |
| 3IFA | C | 1.26 | 16.91 | 16.91 | 16.91 |
| 1G3L | C | 1.29 | N/A | 21.51 | 21.51 |
| 4KZT | Y | 1.34 | 19.97 | 19.97 | 19.97 |
| 3DBA | B | 1.35 | 1.06 | 1.06 | 1.06 |
| 4AVC | A | 1.45 | 6.46 | 34.25 | 34.25 |
| 1G3L | B | 1.46 | 23.92 | 3.10 | 3.10 |
| 1B4B | A | 1.47 | 11.86 | 11.86 | 11.86 |
| 1B4B | B | 1.47 | 15.46 | 12.62 | 15.46 |
| 3I54 | D | 1.50 | 4.79 | 4.79 | 4.79 |
| 2D60 | A | 1.53 | 19.35 | 19.35 | 19.14 |
| 3HQP | C | 1.55 | 39.17 | 39.17 | N/A |
| 1B4B | C | 1.57 | 15.88 | 20.53 | 15.88 |
| 1ZDS | B | 1.61 | 1.24 | N/A | 1.24 |
| 3R6S | A | 1.63 | 9.64 | 9.64 | 9.64 |
| 4JKT | A | 1.79 | 9.91 | 9.91 | 32.18 |
| 3NWY | F | 1.93 | 20.23 | 26.27 | 20.23 |
| 2HIM | A | 2.03 | 20.25 | 20.25 | 20.25 |
| 2PUV | D | 2.05 | 3.09 | 3.09 | 5.95 |
| 2D5Z | B | 2.07 | 16.89 | 10.24 | 10.24 |
| 1EFA | C | 2.26 | N/A | 2.20 | 2.20 |
| 1EM6 | B | 2.38 | 29.40 | 29.40 | 29.40 |
| 1G3L | A | 2.41 | 21.43 | 21.43 | 21.43 |
| 4ETZ | A | 2.42 | 1.91 | 1.91 | 1.91 |
| 3LSF | B | 2.44 | 16.17 | 12.57 | 8.99 |
| 4HYW | B | 2.50 | 0.45 | 0.45 | 0.45 |
| 2W4I | B | 3.03 | N/A | 0.97 | 0.97 |
| 3UO9 | B | 3.12 | 1.77 | 10.63 | 1.77 |
| 3S87 | A | 3.18 | 2.87 | 2.87 | 32.21 |
| 3MWB | B | 3.48 | 14.86 | 14.86 | 27.12 |
| 1F2U | A | 3.86 | 5.77 | 5.77 | 5.77 |
| 3IRH | B | 4.19 | 9.06 | 9.06 | 9.06 |
| 4NBN | A | 4.21 | 26.25 | 13.41 | 26.25 |
| 3N25 | D | 4.22 | 31.26 | 31.26 | 22.82 |
| 1QW7 | B | 4.28 | 12.53 | N/A | 12.53 |
| 2QMX | B | 5.35 | 12.57 | 14.02 | 14.02 |
| 3E3N | B | 5.38 | 14.32 | 14.32 | 14.32 |
| 2Q8M | B | 6.01 | 17.85 | 17.85 | 17.85 |
| 1I7S | A | 6.48 | 26.56 | 13.08 | 25.22 |
| 4BQH | A | 7.09 | 10.71 | 10.71 | 10.71 |
| 2RDE | A | 7.15 | 2.38 | 2.38 | 2.38 |
| 2NW8 | A | 7.53 | 27.49 | 27.49 | 27.49 |
| 1PCQ | A | 7.70 | 2.85 | 2.85 | 2.85 |
| 2HVW | B | 7.81 | 5.22 | 5.22 | 16.06 |
| 3QEL | C | 8.04 | 11.12 | 11.12 | 39.92 |
| 2IEG | A | 8.19 | 27.78 | 27.78 | 4.73 |
| 1F2U | D | 9.43 | 27.78 | 27.78 | 27.78 |
| 1H9G | A | 9.56 | 33.59 | 33.59 | 38.15 |
| 2NW8 | B | 10.46 | 27.38 | 27.38 | 27.38 |
| 1LVW | C | 10.95 | 29.13 | 29.13 | 15.32 |
| 2D5X | B | 11.71 | 14.86 | 11.50 | 11.04 |
| 2WU1 | A | 11.82 | 24.82 | 24.82 | 24.82 |
| 3KGF | B | 11.83 | 25.66 | 25.66 | 21.34 |
| 3UO9 | D | 14.20 | 13.74 | 32.94 | 32.94 |
| 4KFB | B | 15.10 | 34.30 | 23.05 | 27.30 |
| 3BZ7 | A | 15.74 | 19.04 | 19.04 | 19.04 |
| 3VQ8 | B | 15.93 | 15.37 | 11.51 | 11.51 |
| 2YLO | A | 18.14 | N/A | N/A | N/A |
| 3L3V | A | 19.93 | 26.39 | 26.39 | 11.39 |
| 1MP3 | A | 20.17 | 29.14 | 1.34 | 1.34 |
| 4G1N | A | 20.87 | N/A | 1.51 | 1.51 |
| 4LRL | D | 21.65 | 26.90 | 35.57 | 23.94 |
| 4G1N | D | 22.34 | 2.79 | 2.79 | 2.79 |
| 2ATS | A | 24.90 | 14.23 | 14.23 | 14.23 |
| 2BU6 | A | 26.32 | 2.34 | 2.34 | 2.34 |
| 3FUD | A | 31.96 | 37.19 | 37.19 | 37.19 |
| 1F2U | C | N/A | 5.34 | 5.34 | 5.34 |
| 2VD3 | A | N/A | 15.51 | 33.68 | 8.21 |
| 1CE8 | A | N/A | 14.90 | 30.29 | 14.91 |
| 4KFB | A | N/A | 13.61 | 13.61 | 13.61 |
| 11BG | A | N/A | 20.17 | 20.17 | 6.41 |
| 4EJ8 | A | N/A | 25.79 | 1.88 | 1.88 |
| 1H78 | A | N/A | 32.51 | 32.51 | 32.51 |
| 4I1R | A | N/A | 0.53 | 0.53 | 0.53 |
| 3F6G | A | N/A | 18.83 | 18.83 | 18.83 |
